# Cretaceous environmental changes led to high extinction rates in a hyperdiverse beetle family

**DOI:** 10.1186/s12862-014-0220-1

**Published:** 2014-10-21

**Authors:** Gael J Kergoat, Patrice Bouchard, Anne-Laure Clamens, Jessica L Abbate, Hervé Jourdan, Roula Jabbour-Zahab, Gwenaelle Genson, Laurent Soldati, Fabien L Condamine

**Affiliations:** INRA - UMR 1062 CBGP (INRA, IRD, CIRAD, Montpellier SupAgro), Campus de Baillarguet, 34988 Montferrier-sur-Lez, France; Canadian National Collection of Insects, Arachnids and Nematodes, Agriculture and Agri-Food Canada, 960 Carling Avenue, Ottawa, ON K1A 0C6 Canada; CNRS - UMR 5175 CEFE (CNRS, Université Montpellier 2), 1919 Route de Mende, 34293 Montpellier, France; IRD, UMR 237 IMBE (IRD, Aix-Marseille Université, CNRS, Université d’Avignon et des pays de Vaucluse), Centre IRD de Nouméa, 98848 Nouméa, Nouvelle-Calédonie France; Department of Biological and Environmental Sciences, University of Gothenburg, Box 461, SE-405 30 Göteborg, Sweden

**Keywords:** Cretaceous-palaeogene mass extinction, Cretaceous terrestrial revolution, Dating analyses, Diversification analyses, Environmental changes, Palaeoclimates, Tenebrionidae

## Abstract

**Background:**

As attested by the fossil record, Cretaceous environmental changes have significantly impacted the diversification dynamics of several groups of organisms. A major biome turnover that occurred during this period was the rise of angiosperms starting ca. 125 million years ago. Though there is evidence that the latter promoted the diversification of phytophagous insects, the response of other insect groups to Cretaceous environmental changes is still largely unknown. To gain novel insights on this issue, we assess the diversification dynamics of a hyperdiverse family of detritivorous beetles (Tenebrionidae) using molecular dating and diversification analyses.

**Results:**

Age estimates reveal an origin after the Triassic-Jurassic mass extinction (older than previously thought), followed by the diversification of major lineages during Pangaean and Gondwanan breakups. Dating analyses indicate that arid-adapted species diversified early, while most of the lineages that are adapted to more humid conditions diversified much later. Contrary to other insect groups, we found no support for a positive shift in diversification rates during the Cretaceous; instead there is evidence for an 8.5-fold increase in extinction rates that was not compensated by a joint increase in speciation rates.

**Conclusions:**

We hypothesize that this pattern is better explained by the concomitant reduction of arid environments starting in the mid-Cretaceous, which likely negatively impacted the diversification of arid-adapted species that were predominant at that time.

**Electronic supplementary material:**

The online version of this article (doi:10.1186/s12862-014-0220-1) contains supplementary material, which is available to authorized users.

## Background

In the fossil record of marine and terrestrial taxa, the Cretaceous Period (145–66 Million years ago (Ma)) is usually considered a time of major reorganization and modernization of ecosystems characterized by the extinction of groups that were formerly dominant and the appearance and subsequent diversification of new groups [1,2]. The climate during this period is often considered a textbook example of a greenhouse world [3,4] because of the high average global temperatures that lasted until about 70 Ma. Still, this period had its share of both warming and cooling events, and was notably characterized by an extensive tropical ecosystem [5] and an important reduction of arid zones that started about 110–120 Ma [6,7]. Of particular interest, a period called the Cretaceous terrestrial revolution (KTR; also referred as the angiosperm revolution [1,8]), saw the explosive radiation of angiosperms which rose from 0 to 80% of floral composition between 125–90 Ma [9]. The KTR likely provided new ecological and evolutionary opportunities for insects [10-13] and for several vertebrate clades that underwent major diversification during this period [14,15]. Although it remains challenging to investigate the diversification dynamics of species-rich groups in deep time, ancient clades represent important avenues to assess the impacts of past environmental changes such as those we now face (e.g. see Roelants et al. [16]). However, the picture from the fossil record is incomplete and biased for most species-rich groups such as insects [17]. Thankfully, recent developments of cutting-edge methods that allow estimation of variation in diversification rates among lineages [18,19] provide new tools for assessing the diversification dynamics of groups with a poor fossil record. However, very few studies have addressed the effects of Cretaceous environmental changes, in particular the KTR and the Cretaceous-Palaeogene (K-Pg) mass extinction, using these new methods (but see Lloyd et al. [1] and Meredith et al. [14]). In birds and mammals an increase of diversification rates was revealed, which can be accounted for by high speciation and low extinction rates [9,14,15]. In contrast, the diversification rates in dinosaurs remained constant through the KTR [1].

In insects, the explosive radiation of angiosperms is thought to have provided diversification opportunities for pollinators, leaf-mining flies, as well as butterflies and moths [17]. Though numerous dated phylogenies are available, little information can be gleaned on the diversification dynamics of most major groups or families during the Mesozoic. The most notable exceptions are a study on Lepidoptera [13], which highlighted three increases in diversification rates during the Cretaceous; several studies on ants [11,20] that revealed several diversification bursts about 100 Ma; and a study on carpenter bees [21], which provided support for a K-Pg mass extinction event. In Coleoptera, Hunt et al. [22] also postulated that the diversification of Coleoptera is better explained by the persistence of old lineages (see also Wang et al. [23]) that diversified in multiple niches, rather than high diversification rates or the effect of the Cretaceous rise of angiosperms. That said, at the time this study was conducted, methods that explicitly incorporate birth-death models allowing diversification rates to vary were not widely available, requiring authors to rely instead on sister-clade comparisons.

To provide novel insights on insect diversification dynamics, and especially on insect groups that do not feed on live plant tissues, we investigated how Cretaceous changes impacted the diversification of a hyperdiverse beetle family, the Tenebrionidae (darkling beetles). This family currently encompasses ca. 20,000 described species worldwide, and ranks seventh in terms of species richness within the Coleoptera [24]. Adults and larvae are mostly saprophagous, feeding on decaying vegetation, but some species are also mycetophagous and predatory larvae are known in a few tribes [25]. Most of the extant tenebrionid species are either distributed in arid/semiarid regions (e.g., members of the large subfamily Pimeliinae) or in subtropical/tropical forested regions [25,26]. The habitat preference is so marked in this family that shifts in their abundance and range have been used as indicators of climate change [27]. The tenebrionid fossil record highlights an age as old as the Middle Jurassic for the family [28], and indicates that Mesozoic Pangaean and Gondwanan breakups may be reflected in the disjunct distribution patterns observed in several groups such as the Pimeliinae or the Adeliini [25,29,30]. However, due to its relative scarcity, the fossil record alone is not sufficient to provide an accurate temporal framework for the family.

To gain a better understanding of the age of specific divergence events, we conducted molecular dating analyses using 21 calibration points combined with conservative priors and calibration settings. We then used the resulting timeframe to conduct diversification analyses to investigate the early tempo and mode of diversification of the family in relation to Cretaceous environmental changes. Postulating that past environmental changes may have contributed to the diversification dynamics of Tenebrionidae, as more than half of extant species are adapted to arid/semi-arid conditions, we also assessed whether the rise of angiosperms also coincided with changes in the diversification dynamics of clades that encompass forest-dwelling groups.

## Results and discussion

### Mesozoic origin and diversification of a species-rich beetle family

**Figure 1:**
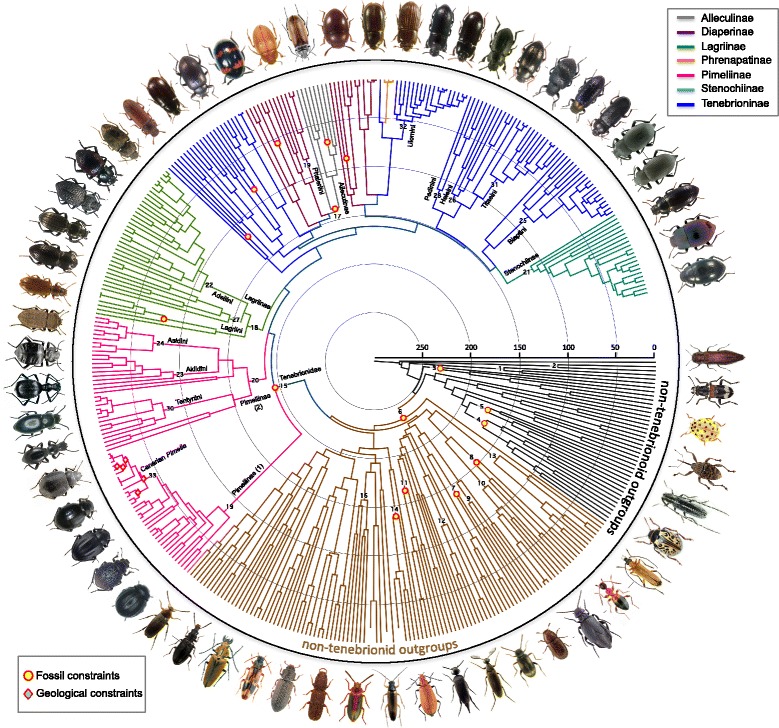
**Circular chronogram corresponding to the best-fit calibration procedure implemented with BEAST (‘Yule crown’).** Contrasting colours are used to highlight major lineages in the tree. The placement of fossil (for crown nodes) and geological constraints is illustrated with yellow circles and green diamonds, respectively. Numbers on nodes correspond to those used in Table 1.

Dating analyses unequivocally support an ancient origin for Tenebrionidae at the end of the Early Jurassic (ca. 180 Ma, Figure 1; Table 1), which significantly predates (about 30 Myrs) previous molecular estimates of tenebrionid ages [22,31]. This earlier date of origin has several consequences on our understanding of tenebrionid origin and evolution against the backdrop of past environmental changes. The first lineages to diversify were a major clade of the paraphyletic subfamily Pimeliinae (ca. 9,000 species; median age of 144.29 Ma, 95% HPD: 116.69-171.77 Ma for the best-fit calibration procedure) and the Lagriinae (ca. 2,000 species; median age of 147.1 Ma, 95% HPD: 132.14-149.53 Ma). For these two groups, extant distribution patterns are consistent with potential vicariance events, as advocated for Adeliini (ca. 400 species; median age of 95.13 Ma, 95% HPD: 82.69-113.95 Ma), which occur today in Australia, Chile, New Caledonia and New Zealand [29,30]. For Pimeliinae, our results support the hypothesis of Matthews et al. [25], who postulated that the highly disjunct extant distributions of several pimeliine tribes are better explained by an ancient origin and Gondwanan fragmentation. The diversification of these two subfamilies was followed by other subfamilies during the KTR (125 to 80 Ma), such as Alleculinae (median age of 126.83 Ma, 95% HPD: 120.0-130.81 Ma), Stenochiinae (median age of 100.22 Ma, 95% HPD: 88.76-109.9 Ma) and several lineages of Diaperinae and Tenebrioninae.

**Table 1:** **Age estimates obtained with a crown calibration scenario using either a BD or a Yule model of speciation**

**Node**	**Clade**	**Min_F**	**Model**	**Node ages**	**95% HPD**
				**Median**	
1	Buprestoidea		BD	131.96	95.58 - 159.96
			Yule	154.40	118.88 - 176.46
2	Byrrhoidea		BD	130.75	57.81 - 234.13
			Yule	124.13	71.31 - 186.65
3	Cleroidea	(166.0)	BD	217.88	185.73 - 235.21
			Yule	219.66	191.95 - 236.60
4	Chrysomeloidea	(152.0)	BD	156.30	152.04 - 177.51
			Yule	155.71	152.00 - 168.20
5	Curculionoidea	(152.0)	BD	164.18	152.03 - 186.74
			Yule	164.71	152.01 - 177.19
6	Tenebrionoidea	(157.0)	BD	218.46	210.27 - 228.34
			Yule	220.89	210.68 - 229.83
7	Aderidae	(120.0)	BD	128.49	120.00 - 142.65
			Yule	130.72	120.02 - 142.83
8	Anthicidae	(120.0)	BD	138.33	120.01 - 162.72
			Yule	136.28	120.00 - 158.92
9	Ciidae		BD	144.13	101.99 - 182.42
			Yule	138.99	93.72 - 182.24
10	Meloidae		BD	122.49	108.72 - 136.86
			Yule	127.77	112.40 - 147.30
11	Mordellidae	(152.0)	BD	152.60	152.03 - 160.35
			Yule	152.74	152.00 - 157.72
12	Mycetophagidae		BD	107.80	90.40 - 129.19
			Yule	104.30	84.94 - 139.08
13	Oedemeridae		BD	116.97	95.37 - 146.98
			Yule	137.42	101.22 - 175.36
14	Pyrochroidae	(126.0)	BD	127.29	126.00 - 135.38
			Yule	127.60	126.00 - 135.56
15	Tenebrionidae	(152.0)	BD	178.04	168.40 - 188.71
			Yule	180.05	169.56 - 191.62
16	Zopheridae		BD	145.85	122.15 - 175.71
			Yule	144.65	120.95 - 164.16
17	Alleculinae	(126.0)	BD	126.82	126.00 - 130.86
			Yule	126.83	126.00 - 130.81
18	Lagriinae		BD	148.03	138.98 - 154.64
			Yule	147.10	132.14 - 149.53
19	Pimeliinae (1)		BD	73.42	59.88 - 97.34
			Yule	72.44	61.63 - 84.12
20	Pimeliinae (2)		BD	146.45	117.91 - 168.19
			Yule	144.29	116.69 - 171.77
21	Stenochiinae		BD	97.85	85.25 - 108.91
			Yule	100.22	88.76 - 109.90
22	Adeliini		BD	91.33	80.72 - 105.69
			Yule	95.13	82.69 - 113.95
23	Akiidini		BD	80.97	59.60 - 101.78
			Yule	82.49	52.03 - 107.92
24	Asidini		BD	62.80	47.94 - 74.39
			Yule	55.66	42.62 - 79.75
25	Blaptini		BD	68.26	53.99 - 79.22
			Yule	64.61	50.34 - 75.20
26	Heleini		BD	94.46	81.94 - 104.39
			Yule	94.53	87.38 - 107.20
27	Lagriini		BD	116.13	102.87 - 128.11
			Yule	119.08	112.07 - 128.01
28	Pedinini		BD	99.93	77.40 - 117.74
			Yule	102.64	81.84 - 115.80
29	Phaleriini		BD	70.64	52.11 - 89.13
			Yule	78.42	55.97 - 94.31
30	Tentyriini		BD	55.05	45.09 - 67.74
			Yule	59.33	37.83 - 63.89
31	Titaeini		BD	58.99	49.29 - 67.79
			Yule	59.51	49.07 - 69.47
32	Ulomini		BD	39.40	34.66 - 43.14
			Yule	40.97	35.58 - 46.27
33	Canarian *Pimelia*		BD	22.57	19.48 - 24.00
			Yule	22.85	20.01 - 24.00

For all calibration schemes the median ages and 95% HPD are reported. Minimum ages for nodes associated with a fossil constraint (Min_F) are also figured.

**Figure 2:**
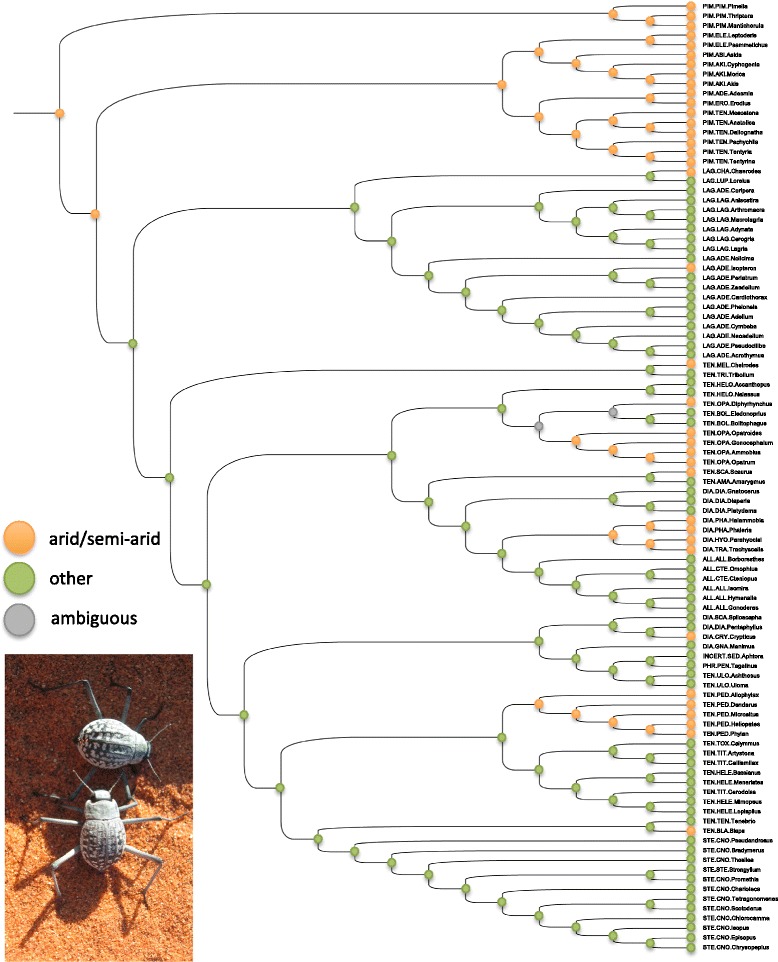
**Maximum likelihood character trait optimization of habitat preferences.** Ancestral character states that are significantly supported are highlighted using either an orange label (for “arid/semi-arid” habitat preferences) or a green label (for “other” habitat preferences). Grey labels indicate ancestral characters for which the difference in log-likelihood is lower than 2.0. For each genus, information on systematics is provided using abbreviated subfamilial and tribal names. A picture of a typical xerophilic species (*Onymacris rugatipennis*) is also included.

Contrary to the arid-dwelling Pimeliinae, the other subfamilies are currently more diverse in tropical environments (see Matthews et al. [25] for details). Thus if we consider that phylogenetic biome conservatism holds in many groups [32], this suggests that distinct tenebrionid lineages were already adapted to arid or tropical environments before the end of the KTR. Results from character optimization analyses support this hypothesis and suggest that the common ancestor of tenebrionid beetles was adapted to arid environments (Figure 2). Arid environments were extremely widespread in the Early Jurassic [33,34]; though they progressively receded with the break-up of the Pangaea [6,7], they never completely disappeared, which could explain the persistence of distinct lineages of pimeliine beetles across eastern and western hemispheres [25]. Interestingly, pimeliine beetles are currently nearly absent from Australia, where aridification began in the Miocene and deserts only appeared very recently [35,36]. As underlined by Matthews et al. [25], this suggests that the group was strictly adapted to arid environments from the beginning, and unable to colonize the Australian continent before its isolation in the middle of the Cenozoic. The only exceptions are the few representatives (nine species) of two plesiotypic tribes (Cnemeplatiini and Vacronini) that were possibly present as coastal sand dune inhabitants before separation from Gondwana [37]. Other groups such as the rain-forest specialists Adeliini [29] originated about 96 Ma (median age of 95.13 Ma, 95% HPD: 82.69-113.95 Ma), possibly in the warm temperate forests that were widespread in the southern hemisphere at that time [4]. This is also the case for other tropical groups in the subfamily Tenebrioninae (tribes Heleini and Titaeini), whose most recent common ancestor diversified at the beginning of the Late Cretaceous, around 100 Ma (median age of 99.83 Ma, 95% HPD: 91.76-111.76 Ma). In some of these groups the phylogenetic biome conservatism is not irreversible, highlighted by the results of character optimizations indicating that several lineages (e.g. Blaptini in Tenebrioninae) became secondarily adapted to arid environments. These secondary shifts likely occurred multiple times during the evolutionary history of darkling beetles, as several subfamilies encompass xerophilic tribes (two tribes out of nine in Lagriinae; 11 tribes out of 29 in Tenebrioninae and five tribes out of 11 in Diaperinae).

### Impacts of cretaceous environmental changes on tenebrionid diversification dynamics

**Figure 3:**
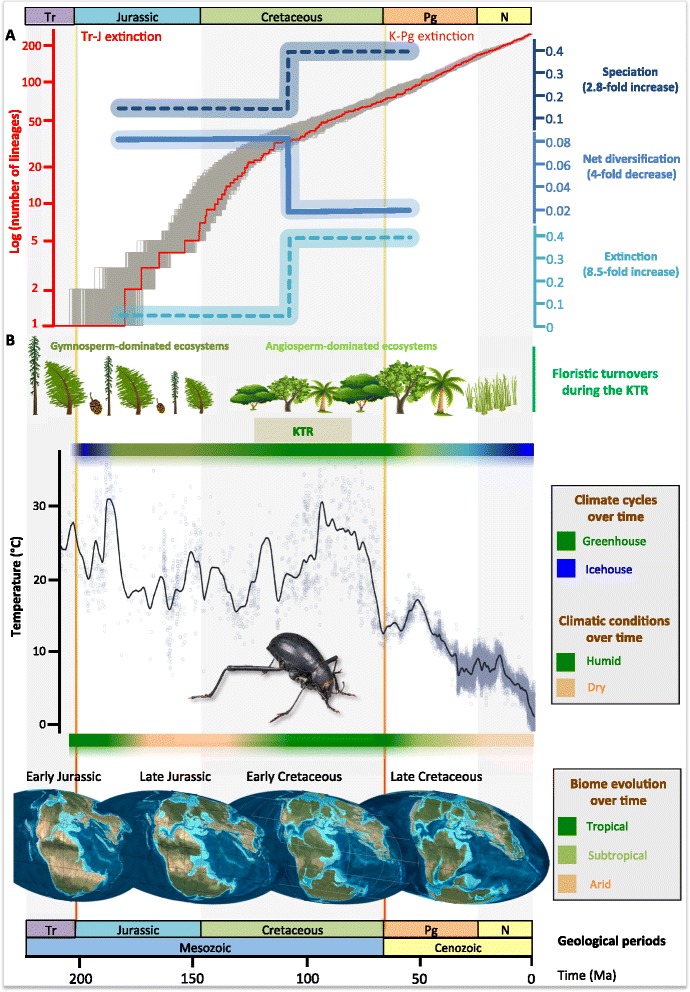
**Diversification dynamics of darkling beetles. A,** Pattern of diversification rates through time of tenebrionids. The lineage-through-time plot is reconstructed with 1,000 random post-burn-in trees (the LTT based on the median values is also figured in red). The results of diversification analyses (net diversification, speciation and extinction rates) are portrayed and indicated the shift during the Cretaceous terrestrial revolution (KTR). **B**, Overview of the possible correlates of diversification for tenebrionids on a global scale. A synthetic view is provided for: (i) the floristic turnovers during the KTR [4-6]; (ii) climate cycles over times; (iii) climatic conditions over time; (iv) changes in global average temperatures (data compiled from various sources [38-40]); and (v) biome evolution over time (paleomaps redrawn from Blakey [41]). A typical arid-adapted tenebrionid (*Onymacris unguicularis*) is also included for illustration purpose.

The lineages-through-time plots suggested a rapid initial diversification, followed by a slowdown in the accumulation of lineages around 100–103 Ma (Figure 3A, Additional file 1: Figure S1). Similar to Meredith et al. [14], we used birth-death models, implemented in the *TreePar* approach, to analyse the time-calibrated trees for the time period spanning 50 Ma to the origin of the group (ca. 180 Ma). We found that a diversification model with varying rates through time was supported by both likelihood ratio tests (*P* <0.001) and corrected Akaike information criteria compared to a constant birth-death model (Table 2). The model with one shift time (two-rate diversification model) fit the trees better (Table 2). Similar trends were also obtained using randomly sampled trees from the posterior trees of the Bayesian dating analysis with the best-fit calibration procedure (Additional file 2: Tables S1-S2).

**Table 2:** **Results of the main TreePar analyses for BD and Yule crown calibration scenarios**

**Trees: BD crown calibration scenario**					
Model descriptions	BD constant	**BD constant 1 shift**	BD constant 2 shifts	BD constant 3 shifts	BD constant 4 shifts
Parameters	2	**5**	8	11	14
-logL	1052,408	**1041,914**	1039,170	1035,864	1033,688
AICc	2108,840	**2093,948**	2094,628	2094,255	2096,215
r1	0,024	**0,021**	0,020	0,019	0,020
τ1	0,922	**0,938**	0,944	0,949	0,946
shift time #1	-	**100**	100	100	100
r2	-	**0,085**	0,121	0,126	0,122
τ2	-	**0,178**	0,000	0,032	0,000
shift time #2	-	**-**	121	121	121
r3	-	**-**	0,065	0,000	0,000
τ3	-	**-**	0,000	0,096	0,263
shift time #3	-	**-**	-	126	126
r4	-	**-**	-	0,075	0,2910
τ4	-	**-**	-	0,062	0,093
shift time #4	-	**-**	-	-	127
r5	-	**-**	-	-	0,068
τ5	-	**-**	-	-	0,013
P (LRT)	null model	**0,0001**	0.1394	0.0598	0.058
**Trees: Yule crown calibration scenario**					
Model descriptions	BD constant	**BD constant 1 shift**	BD constant 2 shifts	BD constant 3 shifts	BD constant 4 shifts
Parameters	2	**5**	8	11	14
-logL	1051,096	**1042,438**	1038,801	1036,949	1035,654
AICc	2106,216	**2094,996**	2093,996	2096,424	2100,146
r1	0,025	**0,022**	0,026	0,025	0,028
τ1	0,920	**0,935**	0,922	0,927	0,910
shift time #1	-	**103**	57	57	57
r2	-	**0,085**	0,038	0,033	0,034
τ2	-	**0,305**	0,749	0,825	0,775
shift time #2	-	**-**	103	103	103
r3	-	**-**	0,070	0,083	0,036
τ3	-	**-**	0,0000	0,000	0,000
shift time #3	-	**-**	-	148	113
r4	-	**-**	-	0,033	0,082
τ4	-	**-**	-	0,0000	0,343
shift time #4	-	**-**	-	-	148
r5	-	**-**	-	-	0,042
τ5	-	**-**	-	-	0,000
P (LRT)	null model	**0,0006**	0.0637	0.0891	0.1385

Both analyses support the same model of diversification (BD constant 1 shift), which is underlined by bold characters.

The period when diversification (=speciation - extinction) and turnovers (=extinction/speciation) changed was inferred to have been the boundary between the Early and Late Cretaceous in the middle of the KTR (shift time at ca. 103 Ma for the best-fit calibration procedure; Figure 3a, Table 1). We found that from the origin of the family to about 103 Ma, the net diversification rates of tenebrionid beetles (mean of 0.085 lineages Myr^−1^) were more elevated than the average net diversification rates for all coleopterans estimated in Hunt et al. [22] (ranging from 0.046 to 0.068 lineages Myr^−1^). Turnovers were not elevated before the shift time (0.305 and 0.178 for the “Yule crown” and “BD crown”, respectively). When calculating speciation and extinction using the diversification rate and turnover ratio, the speciation rate was 0.122 events/lineages Myr^−1^ and the extinction rate was 0.037 events/lineages Myr^−1^ before the shift time (Figure 3A).

After the shift time (103 Ma), turnover rates reached high levels (0.935) while net diversification rates dropped drastically (0.021 lineages/Myr). This important slowdown of net diversification rates (4-fold decrease) resulted from a marked increase of extinction rates (8.5-fold increase, from 0.037 to 0.316 events/lineages Myr^−1^), which was not compensated by a concurrent increase in speciation rates (2.8-fold increase, from 0.122 to 0.338 events/lineages Myr^−1^). Thus, we found no support for a positive upturn in diversification rates during the KTR, contrary to the patterns observed for the diversification of Lepidoptera [13], ants [11,20], mammals [14], and birds [15]. This result suggests that the changes in floral communities concomitant with the radiation of Angiosperms [2] did not significantly foster the diversification of darkling beetles, a pattern in agreement with those observed in several other groups such as dinosaurs [1] and flies [34]. It is worth highlighting that the decrease of net diversification rates we revealed cannot be attributed to the incompleteness of our sampling, because we would not have otherwise been able to capture the simultaneous increase of speciation rates at that time.

To better understand the potential contribution of major abiotic and biotic factors, we provide an overview of the possible correlates of diversification for tenebrionids on a global scale (Figure 3B). Our results allow us to rule out any role of the K-Pg event on the significant increase in extinction rates, as it postdates the shift of diversification rates by about 38 Myr. On the contrary, we postulate that the drastic reduction of arid habitats that started at the end of the Early Cretaceous specifically impacted the diversification of arid-specialist darkling beetles. In particular the subfamily Pimeliinae, currently the single most speciose tenebrionid subfamily, is very conspicuous in desert ecosystems and is found sister to all other tenebrionids, which likely indicates that pimeliines were also predominant during the long-lasting arid episode that persisted between the Late Jurassic and the Early Cretaceous. Therefore the shift toward humid conditions ca. 110–120 Ma and the contractions of desert ecosystems (Figure 3B) probably precipitated the extinction of multiple pimeliine lineages, leaving behind relict lineages with completely disjunct distributions. At least five instances of the latter are known [25] for tribes Caenocrypticini (Namib desert in southern Africa and coastal deserts of Peru and Chile), Cossyphodini (Africa, India and South America), Evaniosomini (Namib desert and South America), Elenophorini (western Mediterranean and southern South America) and Vacronini (North American and central Australian). It is also important to underline that the vast majority of Pimeliinae are flightless [25]; reduced dispersal abilities may have increased their sensitivity to the overall reduction of available habitats at a broader scale [42], limiting their ability to track climate change [43]. While rapid habitat loss may have accelerated the extinction rate of this family dominated at the time by arid-adapted species, one could also imagine that their low vagility may have promoted the speciation of geographically isolated relictual populations through reduced migration between populations at a more local scale [44].

We may hypothesize that high environmental temperatures during the Jurassic and Cretaceous increased rates of biological processes, shortening generation time and thus possibly increasing evolutionary speed [45], leading to relatively high speciation rates. This role of temperature as a key driver of biodiversity is widely acknowledged for large-scale studies in both marine and terrestrial groups [9,46-48]. As tenebrionid beetles are more adapted to arid or tropical regions [25,26,30], they may have benefited overall from the favourable warm climatic conditions that persisted during most of the KTR. After the inferred shift time, the concomitant increase in speciation rate can be attributed to the tropical-adapted lineages. These groups probably took advantage of the expansion of warm tropical and subtropical-forested biomes between the Early and the Late Cretaceous [4], which likely provided them with new ecological opportunities. This hypothesis is also supported by the fact that major tropical groups started their diversification shortly before (Alleculinae) or after (Lagriinae: Adeliini; Stenochiinae; Tenebrioninae: Heleini, Titaeini) the inferred time of environmental shift.

## Conclusions

Overall the global diversification pattern that was inferred for tenebrionid beetles is quite intricate (slowdown of diversification despite an increase of speciation rates) and illustrates the importance of integrating knowledge of the biology of taxa whenever possible. Though we cannot exclude a possible role of the KTR (for the tropical-adapted species), we postulate that the reduction of arid environments that started 110–120 Ma is a key factor that explains the slowdown in diversification dynamics for the family.

## Methods

### Dataset

We used a molecular dataset of eight gene fragments (four mitochondrial and four nuclear) encompassing 404 species (from the study of Kergoat et al. [49]; see also Additional file 3: Table S3). This dataset was first introduced in a study that has mostly focused on discussing tenebrionid relationships [49]. Within the family Tenebrionidae this dataset comprises 250 species (150 of which were collected and sequenced by the authors) belonging to 98 distinct genera. The sampled subfamilies (seven out of nine) represent more than 99% of the tenebrionid species diversity. The sampled tribes (37 tribes out of 96) also encompass most (ca. 77%) of the tenebrionid generic diversity (see Additional file 4: Table S4). For calibration purposes, this dataset includes representatives of several tenebrionid genera (*Bolitophagus*, *Gonocephalum*, *Isomira*, *Lorelus*, *Platydema*, *Pentaphyllus* and *Tribolium*), for which several unambiguous fossil representatives are known [28]. One hundred fifty four species (from 42 beetle families) are also used as outgroups. The rationale here is to sample representatives of deep lineages in order to maximize the number of available calibration points for molecular dating analyses.

### Dating analyses

For all dating analyses, we used a Bayesian relaxed-clock (BRC) approach as implemented in BEAST v1.7.5 [50]. Constraints on clade ages were enforced using sixteen fossil calibrations and five geological calibrations. Fossil constraints were defined following Parham et al. [51] and Sauquet et al. [52] and used to specify minimum ages at nodes (see Additional file 5 for detailed information on each fossil). For all fossil constraints we used the youngest age of the geological stage (using the latest geological time scale from Gradstein et al. [53]) or the youngest age known for a specific amber deposit as minimum age for the calibration to avoid any overestimation of fossil age [52,54]. For geological calibrations, we relied on taxa endemic from particular islands of the Canary archipelago, which are commonly used for molecular dating analyses [54,55]. Because the geological history of these volcanic islands is well documented [56], it allows the specification of maximum age constraints for each island. We used conservative estimates for all islands, namely: 20 Ma for Fuerteventura; 15.5 Ma for Lanzarote; 16 Ma for Gran Canaria; 12 Ma for La Gomera; 11.6 Ma for Tenerife; 2 Ma for La Palma and 1.1 Ma for El Hierro [56]. Given that the magmatic event represented by the Canary Islands started 24 Ma, we set a maximum age of 24.0 Myr (corresponding to the age of the two oldest islands) for the node leading to the clade encompassing the 13 *Pimelia* endemic to the Canary Islands. A maximum age of 16.0 Myr was set for the clade including the three subspecies of *P. sparsa* that are only found in Gran Canaria. A maximum age of 11.6 Myr was set for the clade encompassing *P. canariensis*, *P. radula ascendens*, *P. r. radula* and *P. r. granulata*, which are all endemic to Tenerife. A maximum age constraint of 12.0 Myr was set for the clade encompassing *P. laevigata laevigata* (endemic to La Palma), *P. l. validipes* (endemic to La Gomera) and *P. l. costipennis* (endemic to El Hierro). Finally a maximum age constraint of 1.1 Myr was set for the clade encompassing *P. l. validipes* (endemic to La Gomera) and *P. l. costipennis* (endemic to El Hierro). Finally, to optimize the exploration of parameter space and avoid overestimation of root age we specify a conservative maximum age of 265 million years (Myr) for the root, which significantly predates any known fossil occurrence for the clade that encompasses the sampled superfamilies [17].

BEAST analyses were implemented with partitioned relaxed-clock models (one uncorrelated lognormal clock per gene). In order to limit the numbers of parameter to estimate, we used a guide tree that corresponds to the best maximum likelihood tree inferred with the same dataset in the study of Kergoat et al. [49]. Fossil constraints were either placed on crown or stem nodes. Fossil constraints put on stems are generally more conservative, especially if the sampling of the group is not enough to provide a relevant representation of the clade diversity [52,57]. On the other hand, fossil constraints put on stems can also result in severe biases and underestimate the age of a group if the sister clade is too distantly related [58,59]. Since our taxon sampling is far from being complete and given the difficulty to assign a calibration to a precise node, we adopt a conservative way in which all node calibrations were enforced using uniform distributions (hard-bound constraints [49,60,61]). To account for the fact that our trees describe inter-specific relationships, we also perform distinct analyses with two distinct tree speciation priors: birth-death (BD) and Yule.

For each of the four distinct calibration procedures, two distinct runs were carried out with 50 million generations and trees sampled every 5,000 generations (10,000 trees were sampled for each run). BEAST .xml files were also modified to implement the path-sampling procedure [62], which allows an unbiased approximation of the marginal likelihood of runs [63]. The latter is critical to properly infer the Bayes factors that are used to sort among competing calibration procedures [63]. We used a conservative burn-in-period of 12.5 million generations per run. Post burn-in trees from the two distinct runs (7,500 trees for each run) were further combined using the LogCombiner module of BEAST. Convergence of runs was assessed graphically under Tracer v1.5 [64] and by examining the effective sample size (ESS) of parameters. Bayes factors [65] were then estimated using the log files of the four distinct calibration procedures, using scripts detailed in Baele et al. [63]. Convergence was indicated by ESS of parameters >200 for the post burn-in trees. Dating analyses based on stem calibrations recovered several age estimates that were not consistent with the fossil record (see Additional file 6: Table S5), and were thus discarded. Out of the two crown calibrations, the “Yule crown” calibration procedure was recovered as the best-fit calibration procedure by the Bayes factor comparison (Additional file 7: Table S6). A median age of 180.05 Ma (95% HPD: 169.56-191.62; “Yule crown”) was estimated for the Tenebrionidae (178.04 Ma for the “BD crown”)(see also Additional file 8: Figure S2, Additional file 9: Figure S3).

### Diversification analyses

Diversification analyses were conducted on chronograms resulting from BRC calibration procedures. Analyses were realized under the R environment software implementing the *TreePar* [18] package. To visualize the tempo and mode of diversification of the group, we first reconstructed lineages-through-time plots. We then assessed whether diversification rates remained constant during the early evolutionary history of darkling beetles. Similar to the way Meredith et al. [14] examined the diversification rates of mammals and the impact of the K-Pg extinction event, we focused on the time period between the estimated origin of the family and 50 Ma (early Eocene). We did not examine the tempo or mode of diversification of the group after this date because our chronograms included only a small fraction of the standing diversity of crown-group tenebrionids that were likely extant in the Cenozoic. We are aware of potential biases in diversification rate analyses that use incomplete phylogenies [66,67]. However, our aim was not to reveal a precise diversification pattern but instead to get a broad sense of the early diversification dynamics. Several previous studies on diversification rates for highly incomplete phylogenies have revealed interesting evolutionary patterns despite methodological limitations of their trait-dependent analyses (e.g., Goldberg et al. [68] and Hugall & Stuart-Fox [69]), which are even more sensitive to the missing lineages than are those used in the current study [67].

The *TreePar* package was used to assess speciation and extinction rates through time. This method relaxes the assumption of constant rates by allowing rates to change at specific points in time [18]. Such a model allows for the detection of rapid changes in speciation and extinction rates due to environmental factors like the K-Pg mass extinction. We employed the ‘*bd.shifts.optim*’ function that allows for estimating discrete changes in speciation and extinction rates and mass extinction events in under-sampled phylogenies [18]. Going backward in time, it estimates the maximum likelihood speciation and extinction rates together with the rate shift times *t* = (t_1_,t_2_,…,t_n_) in a phylogeny. At each time *t*, the rates are allowed to change and the species may undergo a mass extinction event. *TreePar* analyses were run with the following settings: start =50, end = crown age estimated by dating analyses, grid =1 Myr, four possible shift times were tested, and posdiv = FALSE to allow the diversification rate to be negative (*i.e.* allows for periods of declining diversity). As our taxon sampling did not include all lineages between the origin of the group and 50 Ma, we adjusted the sampling fraction accordingly. This value was computed using the inferred diversity at 50 Ma divided by the number of lineages we had in the phylogeny. We first estimated a whole diversification rate for Tenebrionidae using the method of moments of Magallón & Sanderson [70] with three relative extinction rates (ε = 0/0.5/0.9) and the current total diversity of the group (20,000 species). We then calculated the diversity at 50 Ma using the following formula:

$$ diversit{y}_{50\mathrm{Ma}}=\frac{20000}{50\kern0.5em \times \kern0.5em  inferred\kern0.5em  diversification\kern0.5em  rate} $$

We investigated the robustness of our results with the maximum clade credibility tree. We took into account the effect of dating uncertainties by repeating the diversification analyses on 500 and 1,000 random chronograms sampled from the Bayesian posterior distributions. The latter allowed us to estimate the mean of all free parameters and their standard errors. Given the diversification rate (=speciation – extinction) and turnover (=extinction/speciation), we inferred the speciation and extinction rate as follows:

$$ speciation\kern0.5em  rate=\frac{diversification\kern0.5em  rate}{1- turnover} $$

and

$$ extinction\kern0.5em  rate=\frac{diversification\kern0.5em  rate\kern0.5em \times \kern0.5em  turnover}{1- turnover} $$

Using the method of moments and three relative extinction rates (ε = 0⁄0.5⁄0.9), the tenebrionid diversification rates were estimated at 0.0529⁄0.0513⁄0.0434 lineages Myr^−1^. Given the current diversity and the diversification rates, we inferred the diversity at 50 Ma around 7556/7800/9218 species (see formula above). Knowing the number of sampled lineages, we deduced the following sampling fraction 0.0265⁄0.0256⁄0.0217 for our phylogenies at 50 Ma. Thus, we conservatively set the sampling fraction to 0.03 in the *TreePar* analyses.

### Character trait optimizations

Character trait optimization analyses were conducted to investigate the phylogenetic biome conservatism of the sampled tenebrionid lineages. We used as a guide tree the best tree inferred under maximum likelihood from the study of Kergoat et al. [49]. This tree was further modified by removing all outgroup taxa and retaining only one species (randomly chosen among specimens with fewest missing data) per tenebrionid genus. Habitat preferences were subsequently coded for the corresponding 98 genera (Additional file 10: Table S7), using a two-state categorization (“arid/semi-arid” and “other”). Ancestral character state estimations were further carried out under maximum likelihood using a one-parameter Markov k-state model with symmetrical rates [71], as implemented in Mesquite 2.75 [72]. The support of one state over another (at a given node) was considered as significant if the difference between their log-likelihoods was greater than or equal to 2.0 [73].

### Limitations of the taxon sampling on the analyses

Conclusions about the temporal nature of diversification are dependent upon the quality of the data [74]. Thus there are important methodological points that may affect analyses and results presented here.

An important methodological issue might be the estimates of divergence times, which are highly suspected to introduce error into diversification analyses or distorted conclusions [74-76]. For instance, too few fossils, failure to use an appropriate prior distribution, or poor calibration choices can lead to inaccurate and illusory dating results (e.g. [61]). These problems may be important as all the post-tree analyses rely on the branching times of the group. Here we have assessed the divergence times of tenebrionids using a rigorous analytic pipeline with BRC analyses, all unambiguous described fossils plus external calibrations; we also repeated the analyses to check likelihood congruence and to cross validate our results. Our dating analyses provided similar divergence times for the phylogeny, and diversification rate analyses were congruent.

Incomplete taxon sampling is a potentially severe problem that is difficult to address. First, missing lineages can lead to inaccurate phylogenetic reconstructions, and anomalous branch lengths can, in turn, bias dating analyses. In our study, we tried to recover the densest taxon sampling that restricts these potential effects, and we have taken into account missing lineages in our analyses and interpretation. Second, missing taxon sampling can be a problem for selecting the best-fit model of diversification. Spurious results of diversification rates can be inferred with under-sampled phylogenies [66]. Rabosky [76] explained that the main issue is not the quality of the taxon sampling, but rather whether a rate is informative if diversification is diversity-dependent. Although it is difficult to detect and incorporate diversity-dependence in the diversification analyses at our level, it could be nonetheless interesting to investigate whether subclades are closer to their diversity limits. A study on the genus *Blaps* (Tenebrionidae: Blaptini) found no effect of diversity-dependence on its diversification [54].

In addition, there are inherent limitations for reconstructing ancestral character states, and thus for inferring when a biome shift occurred. This might be less of a problem here, as the evolution of biome association is very clear and includes few shifts. As taxonomic sampling becomes more extensive in phylogenetic studies of tenebrionids, there will be opportunities for more thorough comparisons of diversification rate variation and biome evolution.

## Additional files

Additional file 1: Figure S1. Lineages-through-time plots for the four calibration scenarios used in this study. Additional file 2: Table S1. TreePar diversification analyses: means and standard errors based on the analysis of 500 posterior trees. **Table S2.** TreePar diversification analyses: means and standard errors based on the analysis of 1000 posterior trees. Additional file 3: Table S3. Taxon sampling and GenBank accession numbers. Additional file 4: Table S4. Number of genera per tribe. Sampled tribes are highlighted using bold characters. Additional file 5:
**Additional information on fossil taxa and justifications on ages of geological formations and amber deposits.**
Additional file 6: Table S5. Comparison of age estimates (under either a BD or a Yule model of speciation) between crown calibration and stem calibration scenarios. For all calibration schemes the median ages and 95% HPD are reported. Bold italic fonts are used to highlight age estimates that are contradicted by known fossil evidences (for the stem calibration scenario). Additional file 7: Table S6. Bayes factor scores (2 ln BF) from Comparisons of alternative calibration procedures of BRC analyses. Additional file 8: Figure S2. Timetree corresponding to the results of the best--fit calibration procedure implemented with BEAST (‘Yule crown’). Horizontal bars on nodes correspond to the 95% higher posterior probabilities (HPD) of age estimates. Additional file 9: Figure S3. Timetree corresponding to the results of the ‘BD crown’ calibration procedure implemented with BEAST.Horizontal bars on Nodes correspond to the 95% higher posterior probabilities (HPD) of age estimates. Additional file 10: Table S7. Biology/habitat of each of the 98 sampled tenebrionid genera.
