# Supplementary material for: Cretaceous environmental changes led to high extinction rates in a hyperdiverse beetle family
Source: BMC Evol Biol. 2014 Oct 21;14:220. doi: 10.1186/s12862-014-0220-1 (PMC4210489; doi:10.1186/s12862-014-0220-1)
Supplement: Additional file 1: Figure S1. — Lineages-through-time plots for the four calibration scenarios used in this study. [file 12862_2014_220_MOESM1_ESM.pdf]

Additional figure S1

Lineages-through-time plots for the four calibration scenarios used in this study.

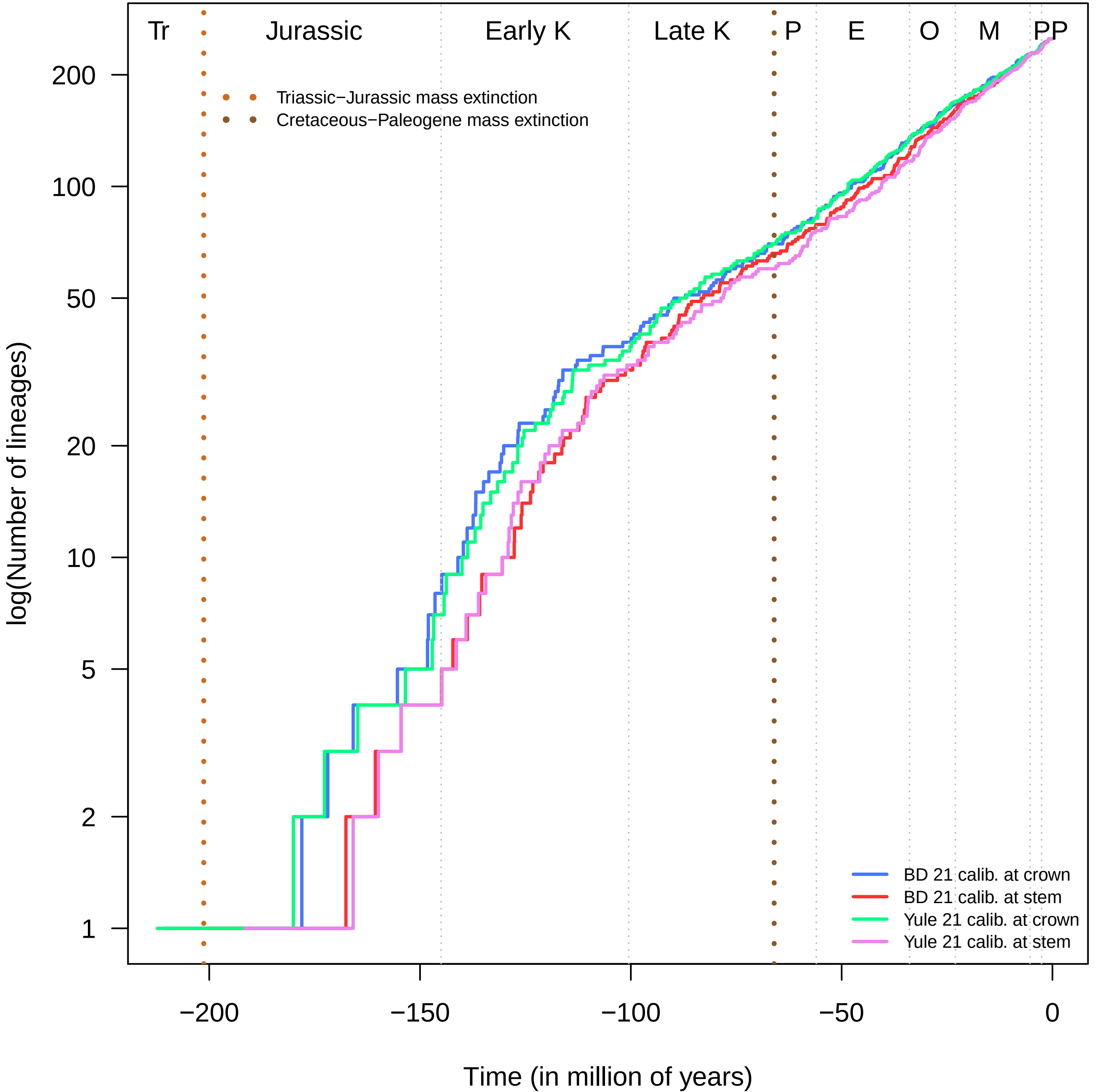

Tr
Jurassic
Early K
Late K
P
E
O
M
PP
Triassic−Jurassic mass extinction
Cretaceous−Paleogene mass extinction
BD 21 calib. at crown
BD 21 calib. at stem
Yule 21 calib. at crown
Yule 21 calib. at stem
200
100
50
20
10
5
2
1
log(Number of lineages)
−200
−150
−100
−50
0
Time (in million of years)
