# Supplementary material for: Cretaceous environmental changes led to high extinction rates in a hyperdiverse beetle family
Source: BMC Evol Biol. 2014 Oct 21;14:220. doi: 10.1186/s12862-014-0220-1 (PMC4210489; doi:10.1186/s12862-014-0220-1)
Supplement: Additional file 2: Table S1. — TreePar diversification analyses: means and standard errors based on the analysis of 500 posterior trees. Table S2. TreePar diversification analyses: means and standard errors based on the analysis of 1000 posterior trees. [file 12862_2014_220_MOESM2_ESM.pdf]

Additional Table S1

TreePar diversification analyses: means and standard errors based on the analysis of 500 posterior trees. The best-fit model - as determined by the corrected Akaike information criterion (AICc) and Likelihood ratio tests (LRT) - is the model with one inferred shift time ('1ShiftTime').

Means (500 trees)

Model	-logL	n param.	AICc	*P* (LRT)	DivRate1	Turnover1	ShiftTime1	DivRate2	Turnover2	ShiftTime2	DivRate3	Turnover3	ShiftTime3	DivRate4	Turnover4	ShiftTime4	DivRate5	Turnover5
NoShiftTime	1055,796	2	2115,641	-	0,0232	0,9181	-	-	-	-	-	-	-	-	-	-	-	-
**1ShiftTime**	**1047,161**	**5**	**2104,568**	**<0,0001**	**0,0200**	**0,9412**	**109,71**	**0,0399**	**0,7741**	**-**	**-**	**-**	**-**	**-**	**-**	**-**	**-**	**-**
2ShiftTimes	1044,857	8	2106,311	0,2028	0,0211	0,9365	94,00	0,0391	0,7638	128,77	0,0019	0,8170	-	-	-	-	-	-
3ShiftTimes	1042,865	11	2108,84	0,1979	0,0213	0,9356	88,73	0,0435	0,7312	109,71	0,0342	0,7275	136,56	-0,0336	0,8674	-	-	-
4ShiftTimes	1041,178	14	2112,143	0,2152	0,0213	0,9356	85,48	0,0333	0,7265	103,72	0,0404	0,7677	120,87	0,0360	0,7578	142,85	-0,0539	0,9013

Standard errors (500 trees)

Model	-logL	n param.	AICc	*P* (LRT)	DivRate1	Turnover1	ShiftTime1	DivRate2	Turnover2	ShiftTime2	DivRate3	Turnover3	ShiftTime3	DivRate4	Turnover4	ShiftTime4	DivRate5	Turnover5
NoShiftTime	0,4782	2	-	-	0,0001	0,0042	-	-	-	-	-	-	-	-	-	-	-	-
**1ShiftTime**	**0,2971**	**5**	**-**	**-**	**0,0001**	**0,0005**	**1,11**	**0,0008**	**0,0123**	**-**	**-**	**-**	**-**	**-**	**-**	**-**	**-**	**-**
2ShiftTimes	0,2954	8	-	-	0,0002	0,0007	1,18	0,0053	0,0126	0,97	0,0090	0,0183	-	-	-	-	-	-
3ShiftTimes	0,2983	11	-	-	0,0002	0,0007	1,18	0,0076	0,0147	1,21	0,0069	0,0168	0,94	0,0130	0,0155	-	-	-
4ShiftTimes	0,3016	14	-	-	0,0002	0,0007	1,16	0,0111	0,0159	1,21	0,0082	0,0180	1,18	0,0076	0,0173	0,94	0,0149	0,0137

Additional Table S2

TreePar diversification analyses: means and standard errors based on the analysis of 1000 posterior trees.

Means (1000 trees)

Model	-logL	n param.	AICc	*P* (LRT)	DivRate1	Turnover1	ShiftTime1	DivRate2	Turnover2
NoShiftTime	1055,177	2	2114,403	-	0,02302	0,92494	-	-	-
**1ShiftTime**	**1047,112**	**5**	**2104,47**	**0,00107**	**0,02004**	**0,94119**	**108,89**	**0,04002**	**0,77719**

Standard errors (1000 trees)

Model	logL	n param.	AICc	*P* (LRT)	DivRate1	Turnover1	ShiftTime1	DivRate2	Turnover2
NoShiftTime	0,252811	2	-	-	5,6E-05	0,00156	-	-	-
**1ShiftTime**	**0,203018**	5	-	-	**7,9E-05**	**0,00035**	**0,79126**	**0,00054**	**0,00833**
