# Supplementary material for: Cretaceous environmental changes led to high extinction rates in a hyperdiverse beetle family
Source: BMC Evol Biol. 2014 Oct 21;14:220. doi: 10.1186/s12862-014-0220-1 (PMC4210489; doi:10.1186/s12862-014-0220-1)
Supplement: Additional file 3: Table S3. — Taxon sampling and GenBank accession numbers. [file 12862_2014_220_MOESM3_ESM.pdf]

Additional Table S3

Taxon sampling and GenBank accession numbers.

Systematics		GenBank accession numbers								
Species	Voucher No.	12S	16S	Cyt b	COI	28S D2D3	28S D4D5	Wg	18S	18S*
**Superfamily BUPRESTOIDEA Leach, 1815**										
**Family BUPRESTIDAE Leach, 1815**										
Genus *Agrilus* Curtis										
Agrilus sinuatus (Olivier, 1790)	**BMNH 679189**	AJ965462	AJ8627310	missing	AJ862795	missing	missing	missing	AF451934	- n/a -
Genus *Anthaxia* Eschscholtz										
Anthaxia hungarica (Scopoli, 1772)	**UPOL 00M24**	missing	DQ198623	missing	DQ198545	missing	DQ198702	missing	DQ100484	- n/a -
Genus *Trachys* Fabricius										
Trachys minutus (Linnaeus, 1758)	**BMNH 679281**	AJ965467	missing	missing	AJ862797	missing	DQ198704	missing	AF451936	- n/a -
**Superfamily BYRRHOIDEA Latreille, 1804**										
**Family DRYOPIDAE Billberg, 1820**										
Genus *Dryops* Olivier										
Dryops costae (Heyden, 1891)	**FC_B14**	missing	EF209436	missing	EF209556	missing	missing	missing	EF209496	- n/a -
Genus *Pomatinus* Sturm										
Pomatinus substriatus (Müller, 1806)	**multiple ind.**	missing	DQ198626	DQ266513	DQ198549	missing	DQ198708	missing	AF451924	- n/a -
**Superfamily CHRYSOMELOIDEA Latreille, 1802**										
**Family CERAMBYCIDAE Latreille, 1802**										
Genus *Agapanthia* Serville										
Agapanthia cardui (Linnaeus, 1767)	**BMNH 704355**	missing	AJ841405	missing	AM283243	missing	AJ841656	missing	AJ841527	- n/a -
Genus *Saperda* Fabricius										
Saperda tridentata Olivier, 1795	**BMNH 704357**	missing	AJ841408	missing	AM283246	missing	AJ841659	missing	AJ841530	- n/a -
Genus *Tetraopes* Schoenherr										
Tetraopes tetraopthalmus (Forster, 1771)	**multiple ind.**	missing	missing	missing	AF267478	FJ000429	missing	missing	FJ000505	- n/a -
Genus *Titanus* Audinet-Serville										
Titanus giganteus (Linnaeus, 1771)	**none**	missing	AJ841414	missing	missing	missing	AJ841668	missing	AJ841539	- n/a -
**Family CHRYSOMELIDAE Latreille, 1802**										

Genus *Calligrapha* Chevrolat										
Calligrapha multipunctata (Say, 1824)	**BMNH 704370**	missing	AJ841303	missing	AM283119	missing	missing	missing	AJ841419	- n/a -
Genus *Chrysolina* Motschulsky										
Chrysolina hyperici (Forster, 1771)	**BMNH 679269**	missing	AF097090	missing	DQ222025	missing	missing	missing	AY748121	- n/a -
Genus *Diabrotica* Dejean										
Diabrotica undecimpunctata Mannerheim, 1843	**BMNH 704464**	missing	AJ781555	missing	AM283202	missing	missing	missing	AJ781618	- n/a -
Genus *Gonioctena* Motschulsky										
Gonioctena olivacea (Forster, 1771)	**multiple ind.**	missing	AJ841310	missing	AY904888	missing	missing	missing	AJ622061	- n/a -
Genus *Pyrrhalta* Joannis										
Pyrrhalta viburni (Paykull, 1799)	**BMNH 704474**	missing	AJ841378	missing	AM283212	missing	missing	missing	AJ841497	- n/a -
Genus *Timarcha* Latreille										
Timarcha tenebricosa (Fabricius, 1775)	**multiple ind.**	missing	AJ231158	missing	AJ171412	FJ000452	missing	missing	FJ000528	- n/a -
**Superfamily CLEROIDEA Latreille, 1802**										
**Family CLERIDAE Latreille, 1802**										
Genus *Clerus* Geoffroy										
Clerus mutillarius Fabricius, 1775	**UPOL 001124**	missing	EF508043	missing	EF508056	missing	missing	missing	EF209691	- n/a -
**Family MELYRIDAE Leach, 1815**										
Genus *Aplocnemus* Stephens										
Aplocnemus sp.	**UPOL 001073**	missing	EF508037	missing	EF508050	missing	missing	missing	EF209702	- n/a -
Genus *Carphurus* Erichson										
Carphurus sp.	**UPOL 001116**	missing	EF508040	missing	EF508053	missing	missing	missing	EF209731	- n/a -
Genus *Falsomelyris* Pic										
Falsomelyris granulata (Fabricius, 1792)	**UPOL 001077**	missing	EF508038	missing	EF508051	missing	missing	missing	EF209700	- n/a -
**Family PRIONOCERIDAE Lacordaire, 1857**										
Genus *Idgia* Laporte										
Idgia sp.	**UPOL ZL0103**	missing	EF490157	missing	EF490157	missing	FJ903952	missing	EF209685	- n/a -
**Family TROGOSSITIDAE Latreille, 1802**										
Genus *Temnoscheila* Westwood										
Temnoscheila japonica Reitter, 1875	**UPOL 001118**	missing	EF508041	missing	EF508054	missing	missing	missing	EF209679	- n/a -
**Superfamily CUCUJOIDEA Latreille, 1802**										
**Family ALEXIIDAE Imhoff, 1856**										
Genus *Sphaerosoma* Samouelle										
Sphaerosoma quercus Samouelle, 1819	**BMNH 675565**	missing	EF512337	missing	DQ155803	missing	missing	missing	EF362957	- n/a -
**Family BIPHYLLIDAE LeConte, 1861**										

Genus *Biphyllus* Dejean										
Biphyllus lunatus (Fabricius, 1792)	**BMNH 679141**	missing	DQ202525	missing	missing	missing	DQ202625	missing	AY748139	- n/a -
**Family Bothrideridae Erichson, 1845**										
Genus *Xylariophilus* Pal & Lawrence										
Xylariophilus sp.	**UPOL ZL0152**	missing	EF490158	missing	EF490188	missing	FJ903953	missing	EF209827	- n/a -
**Family Byturidae Gistel, 1848**										
Genus *Byturus* Latreille										
Byturus ochraceus (Scriba, 1790)	**UPOL 002071**	missing	missing	missing	missing	missing	missing	missing	EF209816	- n/a -
**Family Cerolynidae Billberg, 1820**										
Genus *Philothermus* Aubé										
Philothermus sp.	**UPOL 002267**	missing	missing	missing	missing	missing	missing	missing	EF209834	- n/a -
**Family Coccinellidae Latreille, 1807**										
Genus *Coccinella* Linnaeus										
Coccinella septempunctata Linnaeus, 1758	**BMNH 679320**	missing	missing	missing	missing	missing	missing	missing	AY748147	- n/a -
Genus *Psyllobora* Dejean										
Psyllobora vigintiduopunctata (Linnaeus, 1758)	**UPOL 002134**	missing	missing	missing	missing	missing	missing	missing	EF209854	- n/a -
**Family Cucujidae Latreille, 1802**										
Genus *Cucujus* Fabricius										
Cucujus mniszechi Grouvelle, 1874	**UPOL 002084**	missing	missing	missing	missing	missing	missing	missing	EF209775	- n/a -
**Family Endomychidae Leach, 1815**										
Genus *Mycetina* Mulsant										
Mycetina sp.	**UPOL 002083**	missing	missing	missing	missing	missing	missing	missing	EF209845	- n/a -
**Family Erotylidae Latreille, 1802**										
Genus *Tetraphala* Chevrolat										
Tetraphala sp.	**UPOL 002092**	missing	missing	missing	missing	missing	missing	missing	EF209803	- n/a -
**Family Helotidae Chapuis, 1876**										
Genus *Helota* MacLeay										
Helota gemmata Gorham, 1874	**UPOL 002090**	missing	missing	missing	missing	missing	missing	missing	EF209758	- n/a -
**Family Passandridae Blanchard, 1845**										
Genus *Hectarthrum* Newman										
Hectarthrum sp.	**UPOL 002089**	missing	missing	missing	missing	missing	missing	missing	EF209773	- n/a -

**Family SILVANIDAE Kirby, 1837**										
Genus *Dendrophagus* Schoenherr										
Dendrophagus sp.	**UPOL ZL0213**	missing	missing	missing	missing	missing	missing	missing	EF209768	- n/a -
**Superfamily CURCULIONOIDEA Latreille, 1802**										
**Family ANTHRIBIDAE Billberg, 1820**										
Genus *Urodontus* Louw										
Urodontus mesemoides (Louw, 1993)	**BToLDDM0067**	missing	missing	missing	FJ867818	FJ867725	FJ867725	missing	FJ867802	- n/a -
**Family BRENTIDAE Billberg, 1820**										
Genus *Cylas* Latreille										
Cylas formicarius (Fabricius, 1798)	**multiple ind.**	missing	missing	missing	FJ867849	FJ867676	FJ86767	missing	AF515703	- n/a -
**Family CURCULIONIDAE Latreille, 1802**										
Genus *Curculio* Linnaeus										
Curculio glandium Marsham, 1802	**multiple ind.**	missing	AJ495529	AY327749	AY327713	missing	missing	missing	AJ850003	- n/a -
Genus *Tanysphyrus* Germar										
Tanysphyrus lemnae (Fabricius, 1792)	**BMNH 676138**	missing	missing	missing	DQ155948	missing	missing	missing	AJ850023	- n/a -
**Family NEMONYCHIDAE Bedel, 1882**										
Genus *Cimberis* Gozis										
Cimberis pilosa (LeConte, 1876)	**BToLDDM0147**	missing	missing	missing	FJ867848	FJ867673	FJ867673	missing	FJ867746	- n/a -
Genus *Rhynchitomacerinus* Kuschel										
Rhynchitomacerinus kuscheli (Voss, 1952)	**multiple ind.**	missing	missing	missing	FJ867805	FJ867708	FJ867708	missing	AJ850034	- n/a -
**Superfamily ELATEROIDEA Leach, 1815**										
**Family ELATERIDAE Leach, 1815**										
Genus *Agriotes* Eschscholtz										
Agriotes obscurus (Linnaeus, 1758)	**multiple ind.**	missing	DQ198644	missing	HM542030	missing	DQ198734	missing	HQ333805	- n/a -
**Superfamily TENEBRIONOIDEA Latreille, 1802**										
**Family ADERIDAE Csiki, 1909**										
Genus *Aderus* Stephens										
Aderus sp.	**UPOL ZL0197**	missing	missing	missing	FJ904066	missing	FJ903935	missing	EF210021	- n/a -
Genus *Phytobaenus* R.F. Sahlberg										
Phytobaenus amabilis R.F. Sahlberg, 1834	**BMNH 679279**	missing	DQ202594	missing	DQ221961	missing	DQ202659	missing	AY748187	- n/a -
**Family ANTHICIDAE Latreille, 1819**										

Genus *Anthelephila* Hope										
Anthelephila pedestris (Rossi, 1790)	**UPOL ZL0051**	missing	FJ903703	missing	FJ903984	missing	FJ903835	missing	EF210016	- n/a -
Genus *Anthicomorphus* Lewis										
Anthicomorphus suturalis Lewis, 1895	**UPOL ZL0141**	missing	FJ903749	missing	FJ904034	missing	FJ903899	missing	EF210019	- n/a -
Anthicidae Gen. sp. 1	**UPOL ZL0060**	missing	FJ903710	missing	FJ903991	missing	FJ903842	missing	EF210013	- n/a -
Anthicidae Gen. sp. 2	**UPOL ZL0063**	missing	FJ903712	missing	FJ903993	missing	FJ903844	missing	EF210015	- n/a -
Anthicidae Gen. sp. 3	**UPOL ZL0076**	missing	missing	missing	FJ904001	missing	FJ903854	missing	EF210014	- n/a -
**Family Boridae Thomson, 1859**										
Genus *Boros* Herbst										
Boros schneideri (Panzer, 1795)	**UPOL ZL0094**	missing	EF490134	missing	EF490163	missing	FJ903868	missing	EF209989	- n/a -
**Family Ciidae Leach, 1819**										
Genus *Cis* Latreille										
Cis boleti (Scopoli, 1763)	**UPOL ZL0182**	missing	FJ903769	missing	FJ904059	missing	FJ903027	missing	EF209886	- n/a -
Genus *Ennearthron* Mellie										
Ennearthron cornutum (Gyllenhal, 1827)	**g05**	missing	missing	missing	FM877942	missing	missing	missing	FM877865	- n/a -
Genus *Neoennearthron* Miyatake										
Neoennearthron hisamatsui (Miyatake, 1954)	**g64**	missing	missing	missing	FM877943	missing	missing	missing	FM877904	- n/a -
Genus *Rhopalodontus* Mellie										
Rhopalodontus perforatus (Gyllenhal, 1813)	**g03**	missing	missing	missing	FM877952	missing	missing	missing	FM877863	- n/a -
Genus *Sulcacis* Dury										
Sulcacis affinis (Gyllenhal, 1827)	**g04**	missing	missing	missing	FM877947	missing	missing	missing	FM877864	- n/a -
**Family Melandryidae Leach, 1815**										
Genus *Abdera* Stephens										
Abdera quadrifasciata Curtis, 1829	**UPOL ZL0174**	missing	FJ903765	missing	FJ904055	missing	missing	missing	EF209919	- n/a -
Genus *Anisoxya* Mulsant										
Anisoxya fuscula (Illiger, 1798)	**UPOL ZL0162**	missing	FJ903759	missing	FJ904048	missing	FJ903916	missing	EF209915	- n/a -
Genus *Dircaea* Fabricius										
Dircaea sp.	**UPOL ZL0083**	missing	FJ903724	missing	FJ904007	missing	missing	missing	missing	- n/a -
Genus *Enchodes* LeConte										
Enchodes crepusculus Lewis, 1895	**UPOL ZL0124**	missing	FJ903740	missing	FJ904025	missing	FJ903889	missing	EF209895	- n/a -
Genus *Hypulus* Paykull										
Hypulus acutangulus Lewis, 1895	**UPOL ZL0159**	missing	missing	missing	FJ904405	missing	FJ903913	missing	EF209914	- n/a -
Hypulus cingulatus Lewis, 1895	**UPOL ZL0119**	missing	EF490138	missing	EF490166	missing	FJ903884	missing	EF209906	- n/a -
Hypulus quercinus (Quensel, 1790)	**UPOL ZL0158**	missing	FJ903757	missing	FJ904044	missing	FJ903912	missing	EF209913	- n/a -
Genus *Melandrya* Fabricius										
Melandrya barbata (Fabricius, 1792)	**UPOL ZL0156**	missing	FJ903756	missing	FJ904042	missing	FJ903910	missing	EF209897	- n/a -
Melandrya dubia (Schaller, 1783)	**UPOL ZL0155**	missing	FJ903755	missing	FJ904041	missing	FJ903909	missing	EF209899	- n/a -

Melandrya modesta Lewis, 1895	**UPOL ZL0048**	missing	missing	missing	FJ903982	missing	FJ903833	missing	FJ903792	- n/a -
Melandrya pictipennis Lewis, 1895	**UPOL ZL0069**	missing	FJ903716	missing	FJ903998	missing	FJ903850	missing	missing	- n/a -
Melandrya sp.	**UPOL ZL0120**	missing	FJ903739	missing	FJ904024	missing	FJ903885	missing	FJ903797	- n/a -
Genus *Microtonus* LeConte										
Microtonus dimidiatus Marseul, 1876	**UPOL ZL0144**	missing	EF490137	missing	EF490165	missing	FJ903901	missing	EF209896	- n/a -
Microtonus sp.	**UPOL ZL0140**	missing	FJ903748	missing	FJ904033	missing	FJ903898	missing	EF209910	- n/a -
Genus *Mikadonius* Lewis										
Mikadonius gracilis Lewis, 1895	**UPOL ZL0033**	missing	FJ903700	missing	FJ903979	missing	FJ903828	missing	FJ903791	- n/a -
Genus *Orchesia* Latreille										
Orchesia imitans Lewis, 1895	**UPOL ZL0080**	missing	FJ903721	missing	FJ904004	missing	missing	missing	EF209904	- n/a -
Orchesia micans (Panzer, 1795)	**UPOL ZL0157**	missing	missing	missing	FJ904043	missing	FJ903911	missing	EF209912	- n/a -
Orchesia minor Walker, 1837	**UPOL ZL0175**	missing	FJ903766	missing	FJ904056	missing	FJ903923	missing	EF209920	- n/a -
Orchesia undulata Kraatz, 1853	**UPOL ZL0173**	missing	missing	missing	FJ904054	missing	FJ903922	missing	EF209918	- n/a -
Genus *Osphya* Illiger										
Osphya orientalis (Lewis, 1895)	**UPOL ZL0122**	missing	EF490139	missing	EF490167	missing	FJ903887	missing	EF209898	- n/a -
Genus *Phloiotrya* Stephens										
Phloiotrya bellicosa (Lewis, 1895)	**UPOL ZL0012**	missing	FJ903688	missing	FJ903963	missing	FJ903811	missing	EF209900	- n/a -
Phloiotrya flavitarsis (Lewis, 1895)	**UPOL ZL0138**	missing	FJ903746	missing	FJ904007	missing	FJ903896	missing	EF209908	- n/a -
Phloiotrya obscura (Lewis, 1895)	**UPOL ZL0160**	missing	missing	missing	FJ904046	missing	FJ903914	missing	FJ903799	- n/a -
Phloiotrya planiuscula Nomura & Kato, 1959	**UPOL ZL0130**	missing	FJ903741	missing	FJ904026	missing	FJ903890	missing	EF209907	- n/a -
Phloiotrya rufipes (Gyllenhal, 1810)	**UPOL ZL0176**	missing	FJ903767	missing	FJ904057	missing	FJ903924	missing	FJ903800	- n/a -
Genus *Phryganophilus* C.R. Sahlberg										
Phryganophilus ruficollis (Fabricius, 1798)	**UPOL ZL0032**	missing	FJ903699	missing	FJ903978	missing	FJ903827	missing	EF209902	- n/a -
Melandryidae Gen. sp.	**UPOL ZL0081**	missing	FJ903722	missing	FJ904005	missing	FJ903857	missing	FJ903795	- n/a -
**Family Meloidae Gyllenhal, 1810**										
Genus *Epicauta* Dejean										
Epicauta sp.	**UPOL ZL0135**	missing	FJ903744	missing	FJ904029	missing	FJ903894	missing	EF209988	- n/a -
Genus *Horia* Fabricius										
Horia roepkei Betrem, 1929	**UPOL ZL0115**	missing	EF490134	missing	EF490169	missing	FJ903883	missing	EF209984	- n/a -
Genus *Lydomorphus* Fairmaire										
Lydomorphus bifoveiceps (Fairmaire, 1897)	**UPOL ZL0106**	missing	FJ903732	missing	FJ904016	missing	FJ903874	missing	EF209987	- n/a -
Genus *Lytta* Fabricius										
Lytta vesicatoria (Linnaeus, 1758)	**UPOL ZL0005**	missing	EF490140	missing	EF490168	missing	FJ903806	missing	EF209985	- n/a -
Genus *Meloe* Linnaeus										
Meloe decorus Brandt & Erichson, 1832	**UPOL ZL0113**	missing	FJ903737	missing	FJ904022	missing	FJ903881	missing	EF209982	- n/a -
Meloe proscarabaeus Linnaeus, 1758	**UPOL ZL0114**	missing	FJ903738	missing	FJ904023	missing	FJ903882	missing	EF209983	- n/a -
Meloe uralensis Pallas, 1777	**UPOL ZL0112**	missing	FJ903736	missing	FJ904021	missing	FJ903880	missing	EF209981	- n/a -
**Family Mordellidae Latreille, 1802**										
Genus *Glipa* LeConte										

Glipa angustatissima Pic, 1911	**UPOL ZL0137**	missing	FJ903745	missing	FJ904030	missing	FJ903895	missing	EF209927	- n/a -
Glipa ishigakiana Kono, 1932	**UPOL ZL0088**	missing	EF490143	missing	EF490171	missing	FJ903862	missing	EF209921	- n/a -
Genus *Hoshihananomia* Kono										
Hoshihananomia perlata (Sulzer, 1776)	**UPOL ZL0092**	missing	FJ903729	missing	FJ904013	missing	FJ903866	missing	EF209925	- n/a -
Genus *Mordella* Linnaeus										
Mordella brachyura Mulsant, 1856	**UPOL ZL0089**	missing	EF490144	missing	EF490172	missing	FJ903863	missing	EF209922	- n/a -
Genus *Mordellistena* Costa										
Mordellistena brevicauda (Boheman, 1849)	**UPOL ZL0070**	missing	FJ903717	missing	missing	missing	FJ903851	missing	EF209926	- n/a -
Mordellistena neuwaldeggiana (Panzer, 1796)	**UPOL ZL0090**	missing	FJ903727	missing	FJ904010	missing	FJ903864	missing	EF209923	- n/a -
**Family Mycetophagidae Leach, 1815**										
Genus *Litargus* Erichson										
Litargus connexus (Fourcroy, 1785)	**UPOL ZL0151**	missing	FJ903754	missing	FJ904039	missing	FJ903907	missing	EF209883	- n/a -
Litargus sp. 1	**UPOL ZL0145**	missing	EF490145	missing	EF490173	missing	FJ903902	missing	EF209880	- n/a -
Litargus sp. 2	**UPOL ZL0146**	missing	FJ903751	missing	FJ904036	missing	FJ903903	missing	EF209881	- n/a -
Genus *Mycetophagus* Hellwig										
Mycetophagus atomarius (Fabricius, 1787)	**UPOL ZL0150**	missing	FJ903753	missing	FJ904038	missing	FJ903906	missing	EF209882	- n/a -
Mycetophagus quadripustulatus (Linnaeus, 1761)	**UPOL ZL0014**	missing	EF490159	missing	missing	missing	FJ903813	missing	EF209884	- n/a -
**Family Oedemeridae Latreille, 1810**										
Genus *Chrysanthia* Schmidt										
Chrysanthia viridissima (Linnaeus, 1758)	**UPOL ZL0010**	missing	FJ903686	missing	FJ903961	missing	FJ903809	missing	EF209973	- n/a -
Genus *Nacerdes* Dejean										
Nacerdes hilleri (Harold, 1878)	**UPOL ZL0015**	missing	EF490146	missing	EF490174	missing	FJ903814	missing	EF209974	- n/a -
Nacerdes umenoi (Kono, 1937)	**UPOL ZL0028**	missing	FJ903696	missing	FJ903974	missing	FJ903823	missing	FJ903790	- n/a -
Genus *Oedemera* Olivier										
Oedemera femorata (Scopoli, 1763)	**UPOL ZL0056**	missing	FJ903708	missing	FJ903989	missing	FJ903840	missing	EF209977	- n/a -
Oedemera nobilis (Scopoli, 1763)	**multiple ind.**	AF232898	missing	missing	DQ221991	missing	missing	missing	AY748202	- n/a -
Oedemera podagrariae (Linnaeus, 1767)	**UPOL ZL0055**	missing	FJ903707	missing	FJ903988	missing	FJ903839	missing	EF209976	- n/a -
Oedemera virescens (Linnaeus, 1767)	**UPOL ZL0007**	missing	missing	missing	FJ903959	missing	FJ903807	missing	EF209972	- n/a -
Oedemera venosa (Lewis, 1795)	**UPOL ZL0062**	missing	FJ903711	missing	FJ903992	missing	FJ903843	missing	EF209975	- n/a -
**Family Pyrochroidae Latreille, 1806**										
Genus *Pedilus* Fischer von Waldheim										
Pedilus okamotoi (Kono, 1935)	**UPOL ZL0123**	missing	missing	missing	EF490175	missing	FJ903888	missing	EF209996	- n/a -
Genus *Pyrochroa* O.F. Müller										
Pyrochroa coccinea (Linnaeus, 1761)	**UPOL ZL0002**	missing	FJ903683	missing	FJ903956	missing	FJ903803	missing	EF209991	- n/a -
Pyrochroa sp.	**UPOL ZL0009**	missing	FJ903685	missing	FJ903960	missing	FJ903808	missing	EF209992	- n/a -
Pyrochroidae Gen. sp. 1	**UPOL ZL0022**	missing	FJ903691	missing	FJ903968	missing	FJ903817	missing	EF209993	- n/a -
Pyrochroidae Gen. sp. 2	**UPOL ZL0165**	missing	FJ903761	missing	FJ904050	missing	FJ903918	missing	EF209997	- n/a -

Pyrochroidae Gen. sp. 3	**UPOL ZL0035**	missing	FJ903701	missing	FJ903980	missing	FJ903830	missing	EF209994	- n/a -
**Family RIPIPHORIDAE Gemminger, 1870**										
Genus *Macrosiagon* Hentz										
Macrosiagon cyaniveste (Marseul, 1876)	**UPOL ZL0086**	missing	FJ903726	missing	FJ904009	missing	FJ903860	missing	EF209933	- n/a -
Genus *Pelecotoma* Fischer von Waldheim										
Pelecotoma fennica (Paykull, 1799)	**UPOL ZL0101**	missing	EF490147	missing	EF490176	missing	FJ903872	missing	EF209931	- n/a -
Genus *Trigonodera* Dejean										
Trigonodera lokejii (Nomura & Nakane, 1959)	**UPOL ZL0084**	missing	FJ903725	missing	FJ904008	missing	FJ903859	missing	EF209932	- n/a -
Ripiphoridae Gen. sp. 1	**UPOL ZL0100**	missing	FJ903731	missing	missing	missing	FJ903871	missing	EF209930	- n/a -
Ripiphoridae Gen. sp. 2	**UPOL ZL0132**	missing	FJ903742	missing	FJ904027	missing	FJ903892	missing	EF209935	- n/a -
Ripiphoridae Gen. sp. 3	**UPOL ZL0214**	missing	FJ903782	missing	FJ904075	missing	FJ903946	missing	EF209936	- n/a -
**Family SALPINGIDAE Leach, 1815**										
Genus *Aegialites* Mannerheim										
Aegialites raikokensis Zerche, 2004	**UPOL ZL0102**	missing	missing	missing	EF490178	missing	FJ903873	missing	EF210001	- n/a -
Genus *Elacatis* Pascoe										
Elacatis sp.	**UPOL ZL0148**	missing	EF490149	missing	EF490179	missing	FJ903904	missing	EF210002	- n/a -
Genus *Henoticonus* Reitter										
Henoticonus sp. 1	**UPOL 002212**	missing	FJ903783	missing	FJ904076	missing	FJ903947	missing	FJ903801	- n/a -
Henoticonus sp. 2	**UPOL ZL0164**	missing	FJ903760	missing	FJ904049	missing	FJ903917	missing	EF210006	- n/a -
Genus *Lissodema* Curtis										
Lissodema sp.	**UPOL ZL0170**	missing	FJ903763	missing	FJ904052	missing	FJ903920	missing	EF210003	- n/a -
Genus *Salpingus* Illiger										
Salpingus sp.	**UPOL ZL0053**	missing	FJ903705	missing	FJ903986	missing	FJ903837	missing	EF210008	- n/a -
Salpingidae Gen. sp. 1	**UPOL ZL0054**	missing	FJ903706	missing	FJ903987	missing	FJ903838	missing	EF210009	- n/a -
Salpingidae Gen. sp. 2	**UPOL ZL0207**	missing	FJ903781	missing	FJ904074	missing	FJ903944	missing	EF210004	- n/a -
**Family SCRAPTIIDAE Gistel, 1848**										
Genus *Anaspis* Geoffroy										
Anaspis frontalis (Linnaeus, 1758)	**UPOL ZL0200**	missing	FJ903776	missing	FJ904069	missing	FJ903938	missing	EF210031	- n/a -
Anaspis hayashii Chujo & Nakane, 1953	**UPOL ZL0189**	missing	FJ903772	missing	FJ904063	missing	FJ903931	missing	EF210028	- n/a -
Anaspis lurida Stephens, 1832	**UPOL ZL0204**	missing	FJ903778	missing	FJ904071	missing	FJ903941	missing	EF210032	- n/a -
Anaspis pulicaria Costa, 1854	**UPOL ZL0206**	missing	FJ903780	missing	FJ904073	missing	FJ903943	missing	EF210034	- n/a -
Anaspis rufilabris (Gyllenhal, 1827)	**UPOL ZL0047**	missing	FJ903702	missing	FJ903981	missing	FJ903832	missing	EF210025	- n/a -
Anaspis thoracica (Linnaeus, 1758)	**UPOL ZL0199**	missing	FJ903775	missing	FJ904068	missing	FJ903937	missing	EF210030	- n/a -
Anaspis trifasciata Chevrolat, 1860	**UPOL ZL0205**	missing	FJ903779	missing	FJ904072	missing	FJ903942	missing	EF210033	- n/a -
Genus *Pentaria* Mulsant										
Pentaria badia (Rosenhauer, 1847)	**UPOL ZL0091**	missing	FJ903728	missing	FJ904011	missing	FJ903865	missing	EF209924	- n/a -
Genus *Scraptia* Latreille										

Scraptia sp. 1	**UPOL ZL0093**	missing	EF490151	missing	EF490181	missing	FJ903867	missing	EF210026	- n/a -
Scraptia sp. 2	**UPOL ZL0187**	missing	FJ903770	missing	FJ904061	missing	FJ903929	missing	EF210023	- n/a -
Scraptia sp. 3	**UPOL ZL0190**	missing	FJ903773	missing	FJ904064	missing	FJ903932	missing	EF210029	- n/a -
Scraptiidae Gen. sp.	**UPOL ZL0188**	missing	FJ903771	missing	FJ904062	missing	FJ903930	missing	EF210027	- n/a -
**Family Stenotrachelidae Thomson, 1859**										
Genus *Sponidium* Casey										
Sponidium pallens (Motschulsky, 1860)	**UPOL ZL0050**	missing	EF490135	missing	FJ903983	missing	FJ903834	missing	EF209980	- n/a -
**Family Tenebrionidae Latreille, 1802**										
Subfamily Alleculinae Laporte, 1840										
Tribe Alleculini Laporte, 1840										
Genus *Borboresthes* Fairmaire										
Borboresthes sp.	**UPOL ZL0068**	missing	FJ903715	missing	FJ903997	missing	FJ903849	missing	missing	- n/a -
Genus *Gonodera* Mulsant										
Gonodera luperus (Herbst, 1783)	**UPOL ZL0024**	missing	FJ903693	missing	FJ903970	missing	FJ903819	missing	EF209958	- n/a -
Genus *Hymenalia* Mulsant										
Hymenalia sp.	**UPOL ZL0030**	missing	FJ903698	missing	FJ903976	missing	FJ903825	missing	EF209961	- n/a -
Genus *Isomira* Mulsant										
Isomira antennata (Panzer, 1798)	**UPOL ZL0065**	missing	FJ903714	missing	FJ903995	missing	FJ903846	missing	EF209965	- n/a -
Isomira sp.	**UPOL ZL0166**	missing	FJ903762	missing	FJ904051	missing	FJ903919	missing	EF209969	- n/a -
Tribe Cteniopodini Solier, 1835										
Genus *Cteniopus* Solier										
Cteniopus sulphureus (Linnaeus, 1758)	**UPOL ZL0001**	missing	FJ903682	missing	FJ903955	missing	FJ903802	missing	EF209948	- n/a -
Genus *Omophlus* Solier										
Omophlus rugosicollis (Brullé, 1832)	**UPOL ZL0023**	missing	FJ903692	missing	FJ903969	missing	FJ903818	missing	EF209957	- n/a -
Cteniopodini Gen. sp.	**UPOL ZL0064**	missing	FJ903713	missing	missing	missing	FJ903845	missing	EF209964	- n/a -
Subfamily Diaperinae Latreille, 1802										
Tribe Crypticini Brullé, 1832										
Genus *Crypticus* Latreille										
Crypticus gibbulus (Quensel, 1806)	**LSOL.00074**	KJ002795	missing	KJ003076	KJ003228	missing	missing	KJ003376	KJ003572	KJ003717
Crypticus quisquilius pyrenaicus Baudi, 1876	**LSOL.00136**	KJ002796	missing	missing	KJ003229	missing	missing	KJ003377	missing	- n/a -
Crypticus quisquilius quisquilius (Linnaeus, 1761)	**UPOL ZL0066**	missing	missing	missing	FJ903996	missing	FJ903847	missing	EF209966	- n/a -
Tribe Diaperini Latreille, 1802										
Genus *Diaperis* Geoffroy										
Diaperis boleti (Linnaeus, 1758)	**UPOL ZL0003**	missing	FJ903684	missing	FJ903957	missing	FJ903804	missing	EF209945	- n/a -

Diaperis lewisi Bates, 1873	**UPOL ZL0074**	missing	EF490153	missing	EF490183	missing	FJ903852	missing	EF209946	- n/a -
Genus *Gnatocerus* Thunberg										
Gnatocerus cornutus (Fabricius, 1798)	**none**	missing	EU048290	missing	EU048282	EU048306	EU048306	EU048298	missing	- n/a -
Genus *Pentaphyllus* Latreille										
Pentaphyllus chrysomeloides (Rossi, 1792)	**LSOL.00071**	KJ002797	missing	KJ003077	KJ003230	KJ003476	missing	KJ003378	KJ003573	KJ003718
Pentaphyllus testaceus (Hellwig, 1792)	**673329**	missing	missing	missing	missing	missing	missing	missing	EF362954	- n/a -
Genus *Platydema* Laporte de Castelnau & Brullé										
Platydema striatum (Montrouzier, 1860)	**LSOL.01287**	KJ002798	missing	missing	missing	missing	missing	missing	KJ003574	KJ003719
Platydema sp. 1	**LSOL.01279**	KJ002799	KJ002970	missing	missing	KJ003477	missing	missing	KJ003575	KJ003720
Platydema sp. 2	**LSOL.01929**	KJ002800	missing	missing	missing	missing	missing	missing	missing	- n/a -
Tribe Gnathidiini Gebien, 1921										
Genus *Menimus* Sharp										
Menimus crassus Broun, 1880	**LSOL.01716**	KJ002801	KJ002971	KJ003078	KJ003231	KJ003478	missing	missing	KJ003576	KJ003721
Menimus hydrovatinus (Fauvel, 1904)	**LSOL.01113**	KJ002802	KJ002972	KJ003079	KJ003232	KJ003479	missing	missing	KJ003577	KJ003722
Menimus setosellus (Fauvel, 1904)	**LSOL.01219**	KJ002803	KJ002973	KJ003080	missing	KJ003480	missing	missing	KJ003578	KJ003723
Menimus sp. 1	**LSOL.01216**	KJ002805	KJ002976	KJ003083	KJ003233	missing	missing	missing	KJ003580	- n/a -
Menimus sp. 2	**LSOL.01822**	KJ002804	KJ002974	KJ003081	missing	missing	missing	missing	missing	- n/a -
Menimus sp. 3	**LSOL.01866**	missing	KJ002975	KJ003082	missing	missing	missing	missing	KJ003579	KJ003724
Tribe Hyociini Medvedev and Lawrence, 1982										
Genus *Parahyocis* Kaszab										
Parahyocis championi (Fauvel, 1904)	**LSOL.02002**	KJ002806	KJ002977	KJ003084	missing	KJ003481	missing	missing	KJ003581	KJ003725
Tribe Phaleriini Latreille, 1802										
Genus *Halammobia* Semenov										
Halammobia pellucida (Herbst, 1799)	**LSOL.00042**	KJ002807	missing	KJ003085	KJ003234	KJ003482	missing	KJ003379	KJ003582	KJ003726
Genus *Phaleria* Latreille										
Phaleria bimaculata (Linnaeus, 1767)	**LSOL.00046**	KJ002808	missing	missing	KJ003235	missing	missing	missing	missing	- n/a -
Phaleria cadaverina (Fabricius, 1792)	**LSOL.00057**	KJ002809	missing	KJ003086	KJ003236	missing	missing	KJ003380	KJ003583	KJ003727
Phaleria prolixa (Fairmaire, 1869)	**LSOL.00105**	KJ002810	missing	KJ003087	KJ003237	missing	missing	KJ003381	missing	- n/a -
Tribe Scaphidemini Reitter, 1922										
Genus *Spiloscapha* Bates										
Spiloscapha sp.	**LSOL.01667**	KJ002811	KJ002978	missing	missing	missing	missing	missing	missing	KJ003728
Tribe Trachyscelini Blanchard, 1845										
Genus *Trachyscelis* Latreille										
Trachyscelis aphodioides Latreille, 1809	**LSOL.00055**	KJ002812	missing	missing	KJ003238	KJ003483	missing	KJ003382	KJ003584	KJ003729
Trachyscelis ciliaris Champion, 1893	**LSOL.01658**	KJ002813	KJ002979	KJ003088	KJ003239	KJ003484	missing	missing	KJ003585	KJ003730
Subfamily Lagriinae Latreille, 1825										

Tribe ADELIINI Kirby, 1828										
Genus *Adelium* Kirby										
Adelium alpicola Blackburn, 1892	**LSOL.01692**	KJ002814	KJ002980	KJ003089	KJ003240	KJ003485	missing	KJ003383	KJ003586	KJ003731
Adelium pr. *angulare* Pascoe, 1866	**LSOL.01681**	KJ002815	missing	KJ003090	KJ003241	KJ003486	missing	KJ003384	KJ003587	KJ003732
Adelium sp.	**LSOL.01677**	KJ002816	KJ002981	KJ003091	missing	KJ003487	missing	missing	KJ003588	KJ003733
Genus *Acrothymus* Pascoe										
Acrothymus tristis (Montrouzier, 1860)	**LSOL.01935**	KJ002817	KJ002982	KJ003092	KJ003242	missing	missing	missing	KJ003489	KJ003734
Genus *Cardiothorax* Motschulsky										
Cardiothorax howitti (Pascoe, 1869)	**LSOL.01649**	KJ002818	KJ002983	KJ003093	missing	missing	missing	missing	KJ003590	KJ003735
Cardiothorax laticollis Carter, 1911	**LSOL.01679**	KJ002819	KJ002984	KJ003094	KJ003243	missing	missing	missing	KJ003591	KJ003736
Genus *Coripera* Pascoe										
Coripera morleyana Carter, 1905	**LSOL.01624**	KJ002820	KJ002985	KJ003095	KJ003244	missing	missing	missing	KJ003592	- n/a -
Genus *Cymbeba* Pascoe										
Cymbeba annulipes (Fauvel, 1904)	**LSOL.02272**	KJ002821	KJ002986	KJ003096	KJ003245	KJ003488	missing	missing	KJ003593	KJ003737
Cymbeba bavayi Fauvel, 1904	**LSOL.01798**	KJ002822	missing	KJ003097	missing	missing	missing	missing	KJ003594	KJ003738
Cymbeba trapezus (Fauvel, 1904)	**LSOL.02051**	KJ002826	KJ002990	KJ003101	KJ003249	KJ003489	missing	KJ003385	KJ003596	KJ003739
Cymbeba watti Kaszab, 1982	**LSOL.01093**	KJ002827	missing	missing	missing	missing	missing	missing	missing	- n/a -
Cymbeba sp. 1	**LSOL.01346**	KJ002823	missing	missing	KJ003246	missing	missing	missing	missing	- n/a -
Cymbeba sp. 2	**LSOL.01824**	KJ002824	KJ002987	KJ003098	missing	missing	missing	missing	missing	- n/a -
Cymbeba sp. 3	**LSOL.01889**	KJ002825	KJ002988	KJ003099	KJ003247	missing	missing	missing	missing	- n/a -
Cymbeba sp. 4	**LSOL.02050**	missing	KJ002989	KJ003100	KJ003248	missing	missing	missing	KJ003595	- n/a -
Genus *Isopteron* Hope										
Isopteron sp.	**LSOL.01687**	KJ002828	KJ002991	KJ003102	KJ003250	missing	missing	missing	KJ003597	KJ003740
Genus *Neoadelium* Carter										
Neoadelium fauveli	**LSOL.01864**	KJ002829	KJ002992	KJ003103	KJ003251	KJ003490	missing	KJ003386	KJ003598	KJ003741
Neoadelium genitale	**LSOL.01302**	KJ002830	KJ002993	KJ003104	KJ003252	missing	missing	missing	KJ003599	KJ003742
Genus *Nolicima* Matthews										
Nolicima sp.	**LSOL.01684**	KJ002831	KJ002994	KJ003105	KJ003253	KJ003491	missing	missing	KJ003600	KJ003743
Genus *Periatrum* Sharp										
Periatrum helmsi	**LSOL.01765**	KJ002832	KJ002995	KJ003106	KJ003254	KJ003492	missing	KJ003387	KJ003601	KJ003744
Genus *Pheloneis* Pascoe										
Pheloneis amaroides	**LSOL.01732**	KJ002833	KJ002996	KJ003107	KJ003255	missing	missing	KJ003388	KJ003602	- n/a -
Genus *Pseudocilibe* Kaszab										
Pseudocilibe samuelsoni	**LSOL.01795**	KJ002834	KJ002997	KJ003108	missing	missing	missing	KJ003389	KJ003603	KJ003745
Genus *Zeadelium* Watt										
Zeadelium arthurensis	**LSOL.01752**	KJ002835	KJ002998	KJ003109	KJ003256	KJ003493	missing	KJ003390	KJ003604	KJ003746
Zeadelium complicatum	**LSOL.01733**	KJ002836	KJ002999	KJ003110	KJ003257	KJ003494	missing	KJ003391	KJ003605	KJ003747
Zeadelium thoracicum	**LSOL.01759**	KJ002838	KJ003000	KJ003112	KJ003258	KJ003495	missing	missing	KJ003607	KJ003749
Zeadelium sp.	**LSOL.01751**	KJ002837	missing	KJ003111	missing	missing	missing	missing	KJ003606	KJ003748

Tribe Chaerodini Doyen et al. 1990										
Genus *Chaerodes* White										
Chaerodes trachyscelides White, 1846	**LSOL.01701**	KJ002839	KJ003001	missing	KJ003259	KJ003496	missing	KJ003392	KJ003608	KJ003750
Tribe Lagriini Latreille, 1825										
Genus *Adynata* Fahraeus										
Adynata brevicollis (Fahraeus, 1870)	**UPOL ZL0107**	missing	FJ903733	missing	FJ904017	missing	FJ903875	missing	EF209952	- n/a -
Genus *Anisostira* Borchmann										
Anisostira rugipennis Lewis, 1896	**UPOL ZL0013**	missing	FJ903689	missing	FJ903964	missing	FJ903812	missing	EF209954	- n/a -
Genus *Arthromacra* Kirby										
Arthromacra amamiana Nakane, 1963	**UPOL ZL0029**	missing	FJ903697	missing	FJ903975	missing	FJ903824	missing	EF209971	- n/a -
Arthromacra decora (Marseul, 1876)	**UPOL ZL0052**	missing	FJ903704	missing	FJ903985	missing	FJ903836	missing	FJ903793	- n/a -
Genus *Cerogria* Borchmann										
Cerogria bryanti Borchmann, 1925	**UPOL ZL0011**	missing	FJ903687	missing	FJ903962	missing	FJ903810	missing	EF209951	- n/a -
Genus *Lagria* Fabricius										
Lagria hirta (Linnaeus, 1758)	**LSOL.00127**	KJ002840	missing	KJ003113	KJ003260	KJ003497	FJ903805	KJ003393	KJ003609	KJ003751
Genus *Macrolagria* Lewis										
Macrolagria robusticeps (Lewis, 1895)	**UPOL ZL0025**	missing	missing	missing	FJ903971	missing	FJ903820	missing	EF209959	- n/a -
Tribe Lupropini Ardoin, 1958										
Genus *Lorelus* Sharp										
Lorelus armatus (Montrouzier, 1860)	**LSOL.01294**	KJ002841	missing	KJ003114	missing	KJ003498	missing	missing	KJ003610	KJ003752
Lorelus crassicornis Broun, 1880	**LSOL.01709**	KJ002842	KJ003002	KJ003115	KJ003261	KJ003499	missing	KJ003394	KJ003611	KJ003753
Lorelus mareensis Kaszab, 1982	**LSOL.02035**	KJ002843	KJ003003	missing	KJ003262	missing	missing	missing	KJ003612	- n/a -
Lorelus occularis Fauvel, 1904	**LSOL.01134**	missing	KJ003004	KJ003116	KJ003263	KJ003500	missing	missing	KJ003613	KJ003754
Lorelus priscus Sharp, 1876	**LSOL.01697**	KJ002844	KJ003005	KJ003117	KJ003264	KJ003501	missing	KJ003395	KJ003614	KJ003755
Subfamily Phrenapatinae Solier, 1834										
Tribe Penetini Lacordaire, 1859										
Genus *Tagalinus* Kaszab										
Tagalinus kuscheli Kaszab, 1982	**LSOL.02007**	missing	missing	KJ003118	missing	missing	missing	missing	KJ003616	- n/a -
Tagalinus lifuanus (Montrouzier, 1860)	**LSOL.02023**	missing	missing	KJ003119	missing	KJ003502	missing	missing	KJ003617	- n/a -
Tagalinus sp.	**LSOL.02212**	KJ002846	KJ003006	KJ003120	KJ003265	KJ003503	missing	KJ003397	KJ003618	KJ003757
Subfamily Pimeliinae Latreille, 1802										
Tribe Adesmiini Lacordaire, 1859										
Genus *Adesmia* Fischer										
Adesmia boyeri Solier, 1835	**LSOL.00078**	KJ002847	missing	KJ003121	KJ003266	KJ003504	missing	missing	KJ003619	KJ003758
Tribe Akidini Billberg, 1820										

Genus *Akis* Herbst										
Akis bacarozzo (Schrank, 1786)	**LSOL.Ak.3.2**	KJ002848	KJ003007	KJ003122	KJ003267	missing	missing	missing	missing	- n/a -
Akis genei Solier, 1837	**LSOL Ak.5.1**	KJ002849	KJ003008	KJ003123	KJ003268	missing	missing	missing	missing	- n/a -
Akis italica Solier, 1837	**LSOL Ak.6.1**	KJ002850	KJ003009	KJ003124	KJ003269	missing	missing	missing	missing	- n/a -
Akis spinosa (Linnaeus, 1764)	**LSOL Ak.4.1**	KJ002851	KJ003010	KJ003125	KJ003270	missing	missing	missing	missing	- n/a -
Akis tingitana Lucas, 1859	**LSOL Ak.9.1**	KJ002852	KJ003011	KJ003126	KJ003271	missing	missing	missing	missing	- n/a -
Akis trilineata Herbst, 1799	**LSOL Ak.7.1**	KJ002853	KJ003012	missing	KJ003272	missing	missing	missing	missing	- n/a -
Genus *Cyphogenia* Solier										
Cyphogenia chinensis Faldermann, 1835	**none**	missing	AY663866	missing	missing	missing	missing	missing	AY663859	- n/a -
Genus *Morica* Solier										
Morica grossa (Linnaeus, 1767)	**LSOL.00004**	KJ002854	missing	KJ003127	KJ003273	KJ003505	missing	KJ003398	KJ003620	KJ003759
Tribe Asidini Fleming, 1821										
Genus *Asida* Latreille										
Asida carinata carinata Solier, 1836	**LSOL.00007**	KJ002855	missing	KJ003128	KJ003274	missing	missing	KJ003399	KJ003621	KJ003760
Asida carinata lepidoptera Allard, 1869	**LSOL.00012**	KJ002859	missing	KJ003132	KJ003278	KJ003508	missing	KJ003403	KJ003624	KJ003763
Asida consanguinea Allard, 1869	**LSOL.00076**	KJ002856	missing	KJ003129	KJ003275	KJ003506	missing	KJ003400	missing	- n/a -
Asida corsica Laporte de Castelnau, 1833	**LSOL.00009**	KJ002857	missing	KJ003130	KJ003276	missing	missing	KJ003401	KJ003622	KJ003761
Asida jurinei jurinei Solier, 1836	**LSOL.00011**	KJ002858	missing	KJ003131	KJ003277	KJ003507	missing	KJ003402	KJ003623	KJ003762
Asida jurinei pyrenaea Baudi, 1875	**LSOL.00017**	KJ002860	missing	KJ003133	KJ003279	missing	missing	KJ003404	KJ003625	KJ003764
Asida sabulosa (Fuessly, 1775)	**LSOL.00020**	KJ002861	missing	KJ003134	KJ003280	KJ003509	missing	KJ003405	KJ003626	KJ003765
Asida schusteri Reitter, 1917	**LSOL.00022**	KJ002862	missing	KJ003135	KJ003281	missing	missing	KJ003406	KJ003627	KJ003766
Asida sericea (Olivier, 1795)	**LSOL.00024**	KJ002863	missing	KJ003136	KJ003282	missing	missing	KJ003407	missing	- n/a -
Tribe Elenophorini Solier, 1837										
Genus *Leptoderis* Billberg										
Leptoderis collaris (Linnaeus, 1767)	**LSOL.00034**	KJ002864	missing	KJ003137	KJ003283	missing	missing	KJ003408	KJ003628	KJ003767
Genus *Psammetichus* Latreille										
Psammetichus crassicornis Waterhouse, 1844	**LSOL.00137**	KJ002865	KJ003013	KJ003138	KJ003284	missing	missing	missing	KJ003629	KJ003768
Tribe Erodiini Billberg, 1820										
Genus *Erodius* Fabricius										
Erodius emondi Brullé, 1832	**LSOL.00089**	KJ002866	missing	missing	KJ003285	KJ003510	missing	KJ003409	KJ003630	KJ003769
Erodius orientalis Solier, 1834	**multiple ind**	missing	missing	missing	FN544375	missing	missing	missing	JN619059	- n/a -
Tribe Pimeliini Latreille, 1802										
Genus *Pimelia* Fabricius										
Pimelia atlantis atlantis Solier, 1836	**none**	missing	AJ566062	missing	AJ248202	missing	AJ565956	missing	missing	- n/a -
Pimelia atlantis frigioides Koch, 1941	**none**	missing	AJ566059	AJ565989	AJ248199	missing	AJ565953	missing	missing	- n/a -
Pimelia baetica Solier, 1836	**none**	missing	AJ566067	AJ565996	AJ248206	missing	AJ565961	missing	missing	- n/a -
Pimelia boyeri Solier, 1836	**none**	missing	AJ566060	AJ565990	AJ248200	missing	AJ565954	missing	missing	- n/a -

Pimelia canariensis Brullé, 1838	**multiple ind.**	missing	AJ566049	AJ565979	X97209	missing	AJ565943	missing	missing	- n/a -
Pimelia cordata Kraatz, 1865	**none**	missing	AJ566077	AJ566006	AJ248216	missing	AJ565971	missing	missing	- n/a -
Pimelia criba Solier, 1836	**multiple ind.**	missing	AJ566065	AJ565994	X97222	missing	AJ565959	missing	missing	- n/a -
Pimelia echidna Fairmaire, 1875	**none**	missing	AJ566076	AJ566005	AJ248215	missing	AJ565970	missing	missing	- n/a -
Pimelia elevata Sénac, 1887	**none**	missing	AJ566066	AJ565995	AJ248205	missing	AJ565960	missing	missing	- n/a -
Pimelia estevezi Oromi, 1990	**multiple ind.**	missing	AJ566054	AJ565984	X97210	missing	AJ565948	missing	missing	- n/a -
Pimelia fernandezlopezi Machado, 1979	**multiple ind.**	missing	AJ566055	AJ565985	X97211	missing	AJ565949	missing	missing	- n/a -
Pimelia fornicata Herbst, 1799	**none**	missing	AJ566074	AJ566003	AJ248213	missing	AJ565968	missing	missing	- n/a -
Pimelia granulicollis Wollaston, 1864	**multiple ind.**	missing	AJ566053	AJ565983	X97212	missing	AJ565947	missing	missing	- n/a -
Pimelia integra Rosenhauer, 1856	**none**	missing	AJ566069	AJ565998	AJ248208	missing	AJ565963	missing	missing	- n/a -
Pimelia interjecta Solier, 1836	**multiple ind.**	KJ002867	KJ003014	KJ003139	KJ003286	KJ003511	AJ565958	KJ003410	KJ003631	KJ003770
Pimelia laevigata costipennis Wollaston, 1864	**multiple ind.**	missing	AJ566056	AJ565986	X972213	missing	AJ565950	missing	missing	- n/a -
Pimelia laevigata laevigata Brullé, 1838	**multiple ind.**	missing	AJ566057	AJ565987	X972214	missing	AJ565951	missing	missing	- n/a -
Pimelia laevigata validipes Wollaston, 1864	**multiple ind.**	missing	AJ566058	AJ565988	X972215	missing	AJ565952	missing	missing	- n/a -
Pimelia lutaria Brullé, 1838	**none**	missing	AJ566045	AJ565975	X972216	missing	AJ565939	missing	missing	- n/a -
Pimelia maura Solier, 1836	**none**	missing	AJ566063	AJ565992	AJ248203	missing	AJ565957	missing	missing	- n/a -
Pimelia mauritanica bletoni Antoine, 1949	**none**	missing	AJ566061	AJ565991	AJ248201	missing	AJ565955	missing	missing	- n/a -
Pimelia monticola Rosenhauer, 1856	**none**	missing	AJ566071	AJ566000	AJ248210	missing	AJ565965	missing	missing	- n/a -
Pimelia radula ascendens Wollaston, 1864	**multiple ind.**	missing	AJ566048	AJ565978	X97217	missing	AJ565942	missing	missing	- n/a -
Pimelia radula granulata Wollaston, 1864	**multiple ind.**	missing	AJ566046	AJ565976	X97218	missing	AJ565940	missing	missing	- n/a -
Pimelia radula radula Solier, 1836	**none**	missing	AJ566047	AJ565977	AJ248198	missing	AJ565941	missing	missing	- n/a -
Pimelia rotundipennis Kraatz, 1865	**none**	missing	AJ566073	AJ566002	AJ248212	missing	AJ565967	missing	missing	- n/a -
Pimelia rugosa Fabricius, 1792	**none**	missing	AJ566075	AJ566004	AJ248214	missing	AJ565969	missing	missing	- n/a -
Pimelia scabrosa Solier, 1836	**none**	missing	AJ566072	AJ566001	AJ248211	missing	AJ565966	missing	missing	- n/a -
Pimelia sparsa albohumeralis Lindberg, 1950	**multiple ind.**	missing	AJ566051	AJ565981	X97219	missing	AJ565945	missing	missing	- n/a -
Pimelia sparsa serrimargo Wollaston, 1864	**multiple ind.**	missing	AJ566052	AJ565982	X97220	missing	AJ565946	missing	missing	- n/a -
Pimelia sparsa sparsa Brullé, 1838	**multiple ind.**	missing	AJ566050	AJ565980	X97221	missing	AJ565944	missing	missing	- n/a -
Pimelia subglobosa (Pallas, 1781)	**BMNH 829886**	missing	FN392039	missing	FN391481	missing	FN392096	missing	missing	- n/a -
Genus *Mantichorula* Reitter										
Mantichorula semenowi Reitter, 1889	**none**	missing	AY663885	missing	missing	missing	missing	missing	AY663857	- n/a -
Genus *Thriptera* Solier										
Thriptera kraatzi Haag-Rutenberg, 1876	**LSOL.00134**	KJ002868	missing	KJ003140	KJ003287	missing	missing	missing	KJ003632	KJ003771
Tribe TENTYRIINI Eschscholtz, 1831										
Genus *Anatolica* Eschscholtz										
Anatolica potanini Reitter, 1889	**multiple ind.**	missing	AY663868	EU250298	missing	missing	missing	missing	missing	- n/a -
Genus *Dailognatha* Eschscholtz										
Dailognatha hellenica Reitter, 1897	**BMNH 723106**	missing	FN392001	missing	FM876324	missing	FN392058	missing	missing	- n/a -
Dailognatha quadricollis (Brullé, 1832)	**BMNH 749038**	missing	FN392005	missing	FM876453	missing	FN392063	missing	missing	- n/a -
Genus *Mesostena* Eschscholtz										
Mesostena angulata (Fabricius, 1775)	**LSOL.00060**	KJ002869	missing	KJ003141	KJ003288	KJ003512	missing	KJ003411	KJ003633	KJ003772

Genus *Pachychila* Eschscholtz										
Pachychila germari Solier, 1835	**LSOL.00088**	missing	missing	KJ003142	KJ003289	KJ003513	missing	KJ003412	KJ003634	KJ003773
Genus *Tentyria* Latreille										
Tentyria elongata Waltl, 1835	**LSOL.00122**	KJ002870	missing	KJ003143	missing	KJ003514	missing	KJ003413	KJ003635	KJ003774
Tentyria latreillei Solier, 1835	**LSOL.00053**	KJ002871	missing	KJ003144	missing	KJ003515	missing	KJ003414	KJ003636	KJ003775
Tentyria mucronata Steven, 1829	**LSOL.00052**	KJ002872	missing	KJ003145	KJ003290	KJ003516	missing	KJ003415	KJ003637	KJ003776
Tentyria rotundata Brullé, 1832	**multiple ind.**	missing	FN392043	missing	FN544760	missing	FN392099	missing	missing	- n/a -
Tentyria schaumii Kraatz, 1865	**LSOL.00054**	KJ002873	KJ003015	KJ003146	KJ003291	KJ003517	KJ003370	KJ003416	KJ003638	KJ003777
Genus *Tentyrina* Reitter										
Tentyrina palmeri (Crotch, 1872)	**LSOL.00102**	KJ002874	missing	KJ003147	KJ003292	missing	missing	missing	KJ003639	KJ003778
Subfamily Stenochiinae Kirby, 1837										
Tribe Cnodalonini Oken, 1843										
Genus *Bradymerus* Perroud										
Bradymerus lobicollis Gebien, 1920	**LSOL.00129**	KJ002875	missing	missing	missing	missing	missing	missing	missing	- n/a -
Genus *Charioteca* Pascoe										
Charioteca iris (Fauvel, 1867)	**LSOL.01488**	KJ002879	KJ003019	KJ003150	KJ003295	missing	missing	KJ003419	KJ003642	KJ003781
Charioteca sp. 1	**LSOL.01276**	KJ002876	KJ003016	missing	missing	missing	missing	missing	missing	- n/a -
Charioteca sp. 2	**LSOL.02115**	KJ002877	KJ003017	KJ003148	KJ003293	KJ003518	missing	KJ003417	KJ003640	KJ003779
Charioteca sp. 3	**LSOL.01984**	KJ002878	KJ003018	KJ003149	KJ003294	KJ003519	missing	KJ003418	KJ003641	KJ003780
Genus *Chrysopeplus* Gebien										
Chrysopeplus expolitus Broun, 1880	**LSOL.02321**	KJ002880	KJ003020	KJ003151	KJ003296	KJ003520	missing	missing	KJ003643	KJ003782
Chrysopeplus triregius Watt, 1992	**LSOL.02315**	KJ002881	missing	KJ003152	KJ003297	missing	missing	missing	KJ003644	KJ003783
Genus *Episopus* Bates										
Episopus convexus (Montrouzier, 1860)	**LSOL.02114**	KJ002882	KJ003021	missing	KJ003298	KJ003521	missing	KJ003420	KJ003645	KJ003784
Episopus politus Bates, 1873	**LSOL.01593**	KJ002883	KJ003022	KJ003153	KJ003299	missing	missing	KJ003421	KJ003646	- n/a -
Genus *Isopus* Montrouzier										
Isopus championi (Fauvel, 1904)	**LSOL.01428**	KJ002884	KJ003023	KJ003154	KJ003300	missing	missing	KJ003422	KJ003647	- n/a -
Isopus kuscheli Kaszab, 1982	**LSOL.01538**	KJ002885	KJ003024	KJ003155	KJ003301	missing	missing	KJ003423	missing	- n/a -
Isopus latus Kaszab, 1982	**LSOL.02273**	KJ002886	KJ003025	KJ003156	KJ003302	KJ003522	missing	KJ003424	KJ003648	KJ003785
Isopus montanus Kaszab, 1982	**LSOL.01505**	KJ002887	KJ003026	KJ003157	KJ003303	KJ003523	missing	KJ003425	KJ003649	KJ003786
Isopus orientalis Kaszab, 1982	**LSOL.01502**	KJ002888	KJ003027	KJ003158	KJ003304	missing	missing	KJ003426	KJ003650	KJ003787
Isopus paniei Kaszab, 1982	**LSOL.01455**	KJ002889	KJ003028	KJ003159	KJ003305	KJ003524	missing	KJ003427	KJ003651	- n/a -
Genus *Promethis* Pascoe										
Promethis angulata (Erichson, 1842)	**LSOL.01639**	KJ002890	KJ003029	KJ003160	KJ003306	KJ003525	missing	KJ003428	KJ003652	KJ003788
Genus *Pseudandrosus* Kulzer										
Pseudandrosus caeruleus (Fauvel, 1904)	**LSOL.01275**	KJ002891	KJ003030	KJ003161	KJ003307	KJ003526	missing	missing	KJ003653	KJ003789
Genus *Scotoderus* Perroud										
Scotoderus cancellatus (Montrouzier, 1860)	**LSOL.01996**	KJ002892	KJ003031	KJ003162	KJ003308	missing	missing	missing	KJ003654	- n/a -
Genus *Tetragonomenes* Chevrolat										

Tetragonomenes ruficornis (Champion, 1894)	**LSOL.01669**	KJ002893	KJ003032	KJ003163	KJ003309	KJ003527	missing	KJ003429	KJ003655	KJ003790
Genus *Thesilea* Haag-Rutenberg										
Thesilea baladica (Montrouzier, 1860)	**LSOL.01932**	KJ002894	missing	missing	missing	missing	missing	missing	missing	- n/a -
Tribe STENOCHIINI Kirby, 1837										
Genus *Strongylium* Kirby										
Strongylium sp.	**YY-2004**	missing	missing	missing	AY663875	missing	missing	missing	AY663864	- n/a -
Subfamily TENEBRIONINAE Latreille, 1802										
Tribe AMARYGMINI Gistel, 1848										
Genus *Amarygmus* Dalman										
Amarygmus tristis Blackburn, 1893	**LSOL.01706**	KJ002898	KJ003036	KJ003167	KJ003311	KJ003530	missing	KJ003430	KJ003659	KJ003794
Amarygmus sp. 1	**LSOL.01640**	KJ002896	KJ003034	KJ003165	missing	missing	missing	missing	KJ003657	KJ003792
Amarygmus sp. 2	**LSOL.01674**	KJ002897	KJ003035	KJ003166	missing	KJ003529	missing	missing	KJ003658	KJ003793
Amarygmus pr. *tristis*	**LSOL.01725**	KJ002895	KJ003033	KJ003164	KJ003310	KJ003528	missing	missing	KJ003656	KJ003791
Tribe BLAPTINI Leach, 1815										
Genus *Blaps* Fabricius										
Blaps alternans Brullé, 1838	**LSOL.Bl.56.1**	KC160416	missing	KC160330	missing	missing	missing	missing	missing	- n/a -
Blaps emondi Solier, 1848	**LSOL.Bl.62.1**	KJ002899	KJ003037	KJ003168	KJ003312	missing	missing	missing	missing	- n/a -
Blaps gibba Laporte de Castelnau, 1840	**LSOL.Bl.06.2**	KJ002900	KJ003038	missing	KJ003313	missing	KJ003371	missing	missing	- n/a -
Blaps gigas (Linnaeus, 1767)	**LSOL.00029**	KJ002901	KJ003039	KJ003169	KJ003314	KJ003531	missing	KJ003431	KJ003660	KJ003795
Blaps jeannei Ferrer & Soldati, 1999	**LSOL.Bl.53.1**	KJ002902	missing	KJ003170	KJ003315	missing	missing	missing	missing	- n/a -
Blaps kollarii Seidlitz, 1893	**LSOL.00114**	KJ002903	missing	KJ003171	KJ003316	missing	missing	KJ003432	KJ00661	KJ003796
Blaps lethifera Marsham, 1802	**LSOL.Bl.55.1**	KC160415	missing	KC160329	KC160259	missing	missing	missing	missing	- n/a -
Blaps lusitanica Herbst, 1799	**LSOL.Bl.38.2**	KJ002904	missing	KJ003172	KJ003317	missing	KJ003372	missing	missing	- n/a -
Blaps magica Erichson, 1841	**LSOL.Bl.25.1**	missing	missing	missing	missing	missing	KJ003373	missing	missing	- n/a -
Blaps megalatlantica Koch, 1944	**LSOL.Bl.03.1**	KC160352	missing	KC160277	KC160219	missing	missing	missing	missing	- n/a -
Blaps mucronata Latreille, 1804	**LSOL.00030**	KJ002905	missing	KJ003173	KJ003318	missing	missing	missing	KJ003662	KJ003797
Blaps nefraouensis vespertina Koch, 1937	**LSOL.Bl.49.1**	KJ002906	missing	KJ003174	KJ003319	missing	KJ003374	missing	missing	- n/a -
Blaps nitens Laporte de Castelnau, 1840	**LSOL.00031**	KJ002907	missing	KJ003175	KJ003320	KJ003532	missing	KJ003433	KJ003663	- n/a -
Blaps ocreata Allard, 1880	**LSOL.Bl.54.1**	KC160414	missing	KC160328	missing	missing	missing	missing	missing	- n/a -
Blaps peyerimhoffi Koch, 1944	**LSOL.Bl.02.1**	KC160350	KC160428	KC160276	KC160217	missing	missing	missing	missing	- n/a -
Blaps plana Solier, 1848	**LSOL.Bl.25.1**	KC160389	KC160443	KC160304	KC160241	missing	missing	missing	missing	- n/a -
Blaps propheta propheta Reiche, 1861	**LSOL.Bl.07.1**	KC160360	missing	KC160282	KC160227	missing	missing	missing	missing	- n/a -
Blaps tibialis Reiche & Saulcy, 1857	**LSOL.Bl.40.1**	KJ002908	missing	KJ003176	KJ003321	missing	missing	missing	missing	- n/a -
Tribe BOLITOPHAGINI Kirby, 1837										
Genus *Bolitophagus* Illiger										
Bolitophagus corticola Say, 1825	**none**	missing	missing	missing	missing	FJ000417	missing	missing	missing	- n/a -

Bolitophagus reticulatus (Linnaeus, 1767)	**LSOL.00058**	KJ002909	missing	KJ003177	KJ003322	KJ003533	missing	KJ003434	KJ003664	KJ003798
Genus *Eledonoprius* Reitter										
Eledonoprius armatus (Panzer, 1799)	**LSOL.00069**	KJ002910	missing	KJ003178	KJ003323	KJ003534	missing	KJ003435	KJ003665	KJ003799
Tribe HELEINI Fleming, 1821										
Genus *Bassianus* Matthews & Doyen										
Bassianus sydneyanus (Blackburn, 1893)	**LSOL.01653**	KJ002911	KJ003040	KJ003179	KJ003324	KJ003535	missing	missing	KJ003666	KJ003800
Genus *Lepispilus* Westwood										
Lepispilus ocularis Carter, 1932	**LSOL.01676**	KJ002912	KJ003041	KJ003180	KJ003325	KJ003536	missing	KJ003436	KJ003667	KJ003801
Lepispilus sulcicollis (Boisduval, 1835)	**LSOL.01651**	KJ002914	KJ003043	KJ003182	missing	missing	missing	KJ003438	KJ003669	KJ003803
Lepispilus pr. *sulcicollis* (Boisduval, 1835)	**LSOL.01654**	KJ002913	KJ003042	KJ003181	missing	KJ003537	missing	KJ003437	KJ003668	KJ003802
Genus *Meneristes* Pascoe										
Meneristes australis (Boisduval, 1835)	**LSOL.01647**	KJ002915	KJ003044	KJ003183	KJ003326	KJ003538	missing	KJ003439	KJ003670	KJ003804
Genus *Mimopeus* Pascoe										
Mimopeus elongatus (Breme, 1842)	**LSOL.01702**	KJ002916	KJ003045	KJ003184	missing	KJ003539	missing	KJ003440	KJ003671	KJ003805
Tribe HELOPINI Latreille, 1802										
Genus *Accanthopus* Dejean										
Accanthopus velikensis (Piller & Mitterpacher, 1783)	**LSOL.00126**	KJ002917	missing	KC160266	KJ003327	missing	missing	KJ003441	missing	- n/a -
Genus *Nalassus* Mulsant										
Nalassus dryadophilus (Mulsant, 1854)	**LSOL.00118**	KJ002918	missing	KC160268	KJ003328	KJ003540	missing	KJ003442	KJ003672	KJ003806
Nalassus harpaloides (Küster, 1850)	**LSOL.00064**	KJ002919	missing	KC160269	KJ003329	KJ003541	missing	KJ003443	KJ003673	KJ003807
Tribe MELANIMOMINI Seidlitz, 1894										
Genus *Cheirodes* Gené										
Cheirodes pilosus (Tournier, 1868)	**LSOL.00062**	KJ002920	missing	KJ003185	KJ003330	KJ003542	missing	missing	KJ003674	KJ003808
Tribe OPATRINI Brullé, 1832										
Genus *Ammobius* Guerin-Meneville										
Ammobius rufus (Lucas, 1846)	**LSOL.00036**	KJ002921	missing	missing	missing	KJ003543	missing	KJ003444	KJ003675	KJ003809
Genus *Diphyrhynchus* Fairmaire										
Diphyrhynchus sp. 1	**LSOL.01905**	KJ002922	missing	missing	missing	KJ003544	missing	KJ003445	KJ003676	KJ003810
Diphyrhynchus sp. 2	**LSOL.02003**	KJ002923	missing	KJ003189	KJ003331	missing	missing	missing	missing	- n/a -
Genus *Gonocephalum* Solier										
Gonocephalum adpressiforme Kaszab, 1951	**LSOL.02092**	KJ002924	KJ003046	missing	KJ003332	missing	missing	KJ003446	KJ003677	KJ003811
Gonocephalum yelamosi Español &Viñolas, 1983	**LSOL.00092**	KJ002925	missing	KJ003190	KJ003333	missing	missing	missing	missing	- n/a -
Genus *Opatroides* Brullé										
Opatroides punctulatus Brullé, 1832	**LSOL.00040**	KJ002926	missing	missing	KJ003334	missing	missing	KJ003447	KJ003678	- n/a -
Genus *Opatrum* Fabricius										
Opatrum sabulosum (Linnaeus, 1760)	**LSOL.00041**	KJ002927	missing	KJ003191	KJ003335	KJ003545	missing	KJ003448	KJ003679	KJ003812

Tribe PEDININI Eschscholtz, 1829										
Genus *Allophylax* Bedel										
Allophylax picipes (Oliver, 1811)	**LSOL.00065**	KJ002928	missing	KJ003192	missing	KJ003546	missing	KJ003449	KJ003680	KJ003813
Genus *Dendarus* Latreille										
Dendarus coarcticollis (Mulsant, 1854)	**LSOL.00073**	KJ002929	missing	KJ003193	KJ003336	KJ003547	missing	KJ003450	KJ003681	KJ003814
Genus *Heliopates* Dejean										
Heliopates littoralis Español, 1958	**LSOL.00085**	KJ002930	missing	KJ003194	KJ003337	missing	missing	missing	KJ003682	KJ003815
Genus *Micrositus* Mulsant & Rey										
Micrositus semicostatus Mulsant, 1854	**LSOL.00032**	KJ002931	missing	KJ003195	KJ003338	KJ003548	missing	KJ003451	KJ003683	KJ003816
Genus *Phylan* Stephens										
Phylan gibbus (Fabricius, 1775)	**LSOL.00121**	KJ002932	missing	KJ003196	KJ003339	missing	missing	missing	missing	- n/a -
Tribe SCAURINI Billberg, 1820										
Genus *Scaurus* Fabricius										
Scaurus atratus Fabricius, 1775	**LSOL.00081**	KJ002933	missing	KJ003197	missing	KJ003549	missing	KJ003452	KJ003684	KJ003817
Scaurus striatus Fabricius, 1792	**LSOL.00048**	KJ002934	missing	KJ003198	missing	missing	missing	missing	KJ003685	KJ003818
Scaurus uncinus (Forster, 1771)	**LSOL.00049**	KJ002935	missing	KJ003199	missing	KJ003550	missing	KJ003453	KJ003686	KJ003819
Tribe TENEBRIONINI Latreille, 1802										
Genus *Tenebrio* Linnaeus										
Tenebrio molitor Linnaeus, 1758	**multiple ind.**	missing	AJ438153	missing	EU048284	EU048308	EU048308	EU048300	X07801	- n/a -
Tribe TITAENINI Fauvel, 1905										
Genus *Artystona* Bates										
Artystona rugiceps Bates, 1874	**LSOL.01718**	KJ002936	missing	KJ003200	missing	missing	missing	KJ003454	KJ003687	- n/a -
Artystona sp. 1	**LSOL.01746**	KJ002937	KJ003047	KJ003201	KJ003340	missing	missing	KJ003455	KJ003688	- n/a -
Artystona sp. 2	**LSOL.01768**	KJ002938	KJ003048	KJ003202	KJ003341	missing	missing	KJ003456	KJ003689	- n/a -
Genus *Callismilax* Bates										
Callismilax aenea (Montrouzier, 1860)	**LSOL.01281**	KJ002939	KJ003049	KJ003203	KJ003342	KJ003551	missing	KJ003457	KJ003690	KJ003820
Callismilax auripennis Fauvel, 1905	**LSOL.01604**	KJ002940	KJ003050	KJ003204	KJ003343	missing	missing	KJ003458	KJ003691	KJ003821
Callismilax bavayi Fauvel, 1905	**LSOL.01432**	KJ002941	KJ003051	KJ003205	KJ003344	KJ003552	missing	KJ003459	KJ003692	- n/a -
Callismilax deplanchei (Fauvel, 1868)	**LSOL.01224**	KJ002942	KJ003052	KJ003206	KJ003345	KJ003553	missing	KJ003460	KJ003693	- n/a -
Callismilax gloriosa Fauvel, 1905	**LSOL.02252**	KJ002943	KJ003053	KJ003207	KJ003346	KJ003554	missing	KJ003461	KJ003694	KJ003822
Callismilax plicicollis Fauvel, 1905	**LSOL.01546**	KJ002944	KJ003054	KJ003208	KJ003347	KJ003555	missing	KJ003462	KJ003695	KJ003823
Callismilax sarasini Kaszab, 1982	**LSOL.01289**	KJ002946	KJ003056	KJ003210	KJ003349	KJ003556	missing	KJ003464	KJ003697	- n/a -
Callismilax sulcipennis Fauvel, 1905	**LSOL.01237**	KJ002947	KJ003057	KJ003211	KJ003350	KJ003557	missing	KJ003465	KJ003698	- n/a -
Callismilax variolosa Fauvel, 1905	**LSOL.01515**	KJ002948	KJ003058	KJ003212	KJ003351	KJ003558	missing	KJ003466	KJ003699	KJ003825
Callismilax pr. *ruficornis* Bates, 1874	**LSOL.01500**	KJ002945	KJ003055	KJ003029	KJ003348	missing	missing	KJ003463	KJ003696	KJ003824
Genus *Cerodolus* Sharp										
Cerodolus chrysomeloides Sharp, 1886	**LSOL.01762**	KJ002949	KJ003059	KJ003213	KJ003352	KJ003559	missing	KJ003467	KJ003700	- n/a -

Tribe TOXICINI Lacordaire, 1859										
Genus *Calymmus* Montrouzier										
Calymmus berardi (Montrouzier, 1860)	**LSOL.01796**	KJ002950	missing	KJ003214	missing	missing	missing	missing	missing	- n/a -
Tribe TRIBOLIINI Gistel, 1848										
Genus *Tribolium* MacLeay										
Tribolium castaneum (Herbst, 1797)	**multiple ind.**	AJ312413	KJ003060	KJ003215	KJ003353	KJ003560	KJ003375	KJ003468	KJ003701	KJ003826
Tribolium confusum Jacquelin du Val, 1861	**multiple ind.**	missing	EU048288	missing	FJ743725	EU048304	EU048304	EU048296	missing	- n/a -
Tribe ULOMINI Blanchard, 1845										
Genus *Achthosus* Pascoe										
Achthosus westwoodi Pascoe, 1863	**LSOL.01625**	KJ002952	KJ003061	KJ003216	KJ003354	KJ003561	missing	KJ003469	KJ003702	- n/a -
Genus *Uloma* Dejean										
Uloma apicipennis (Fauvel, 1904)	**LSOL.02248**	KJ002953	KJ003062	KJ003217	missing	KJ003562	missing	missing	KJ003703	- n/a -
Uloma cavicollis Blair, 1940	**LSOL.00996**	KJ002955	KJ003064	KJ003219	KJ003356	KJ003564	missing	KJ003471	KJ003705	KJ003829
Uloma damoiseaui Kaszab, 1982	**LSOL.01048**	KJ002956	missing	missing	KJ003357	missing	missing	missing	missing	- n/a -
Uloma marginatoides Kaszab, 1982	**LSOL.01646**	KJ002960	KJ003067	missing	KJ003361	missing	missing	missing	KJ003708	- n/a -
Uloma microcephala (Fauvel, 1904)	**LSOL.01067**	KJ002961	missing	missing	KJ003362	missing	missing	missing	missing	- n/a -
Uloma miriceps (Fauvel, 1904)	**LSOL.01349**	KJ002962	KJ003068	KJ003222	KJ003363	KJ003567	missing	missing	KJ003709	KJ003832
Uloma opacipennis (Fauvel, 1904)	**LSOL.02250**	KJ002963	KJ003069	KJ003223	KJ003364	KJ003568	missing	KJ003473	KJ003710	KJ003833
Uloma opacoides Kaszab, 1982	**LSOL.01644**	KJ002964	KJ003070	KJ003224	KJ003365	missing	missing	missing	KJ003711	KJ003834
Uloma punctata (Fauvel, 1904)	**LSOL.01268**	KJ002965	KJ003071	KJ003225	KJ003366	KJ003569	missing	KJ003474	KJ003712	KJ003835
Uloma queenslandica Kaszab, 1982	**LSOL.01643**	KJ002966	KJ003072	KJ003226	KJ003367	missing	missing	missing	KJ003713	KJ003836
Uloma rufa (Piller & Mitterpacher, 1783)	**LSOL.U.rufa**	KJ002967	KJ003073	missing	missing	missing	missing	missing	KJ003714	- n/a -
Uloma tenebrioides (White, 1846)	**LSOL.01695**	KJ002969	KJ003075	missing	KJ003369	KJ003571	missing	missing	KJ003704	- n/a -
Uloma sp. 1	**LSOL.01339**	KJ002954	KJ003063	KJ003218	KJ003355	KJ003563	missing	KJ003470	KJ003716	KJ003828
Uloma sp. 2	**LSOL.01337**	KJ002957	KJ003065	KJ003220	KJ003358	KJ003565	missing	KJ003472	KJ003706	KJ003830
Uloma sp. 3	**LSOL.01031**	KJ002958	missing	missing	KJ003359	missing	missing	missing	missing	- n/a -
Uloma sp. 4	**LSOL.01617**	KJ002959	KJ003066	KJ003221	KJ003360	KJ003566	missing	missing	KJ003707	KJ003831
Uloma sp. 5	**LSOL.01673**	KJ002968	KJ003074	KJ003227	KJ003368	KJ003570	missing	missing	KJ003715	KJ003837
INCERTAE SEDIS										
Genus *Aphtora* Bates										
Aphtora rufipes Bates, 1872	**LSOL.01720**	KJ002845	missing	missing	missing	missing	missing	KJ003396	KJ003615	KJ003756
**Family TETRATOMIDAE Billberg, 1820**										
Genus *Hallomenus* Panzer										
Hallomenus binotatus (Quensel, 1790)	**UPOL ZL0171**	missing	FJ903764	missing	FJ904053	missing	FJ903921	missing	EF209917	- n/a -
Genus *Holostrophus* xxxx										
Holostrophus orientalis Lewis, 1795	**UPOL ZL0082**	missing	FJ903723	missing	FJ904006	missing	FJ903859	missing	EF209905	- n/a -

Genus *Mycetoma* Dejean										
Mycetoma suturale (Panzer, 1797)	**UPOL ZL0078**	missing	FJ903719	missing	FJ904002	missing	FJ903855	missing	EF209903	- n/a -
Mycetoma sp.	**UPOL ZL0139**	missing	FJ903747	missing	FJ904032	missing	FJ903897	missing	EF209909	- n/a -
Genus *Penthe* Newman										
Penthe japana Marseul, 1876	**UPOL ZL0026**	missing	FJ903694	missing	FJ903972	missing	FJ903821	missing	FJ903789	- n/a -
Penthe sp.	**UPOL ZL0133**	missing	FJ903743	missing	FJ904028	missing	FJ903893	missing	EF209891	- n/a -
Genus *Synstrophus* Seidlitz										
Synstrophus macrophtalmus (Reitter, 1887)	**UPOL ZL0031**	missing	missing	missing	FJ903977	missing	FJ903826	missing	EF209901	- n/a -
**Family TRICTENOTOMIDAE Blanchard, 1845**										
Genus *Trictenotoma* Gray										
Trictenotoma sp.	**UPOL ZL0208**	missing	EF490155	missing	EF490185	missing	FJ903945	missing	EF209990	- n/a -
**Family ZOPHERIDAE Solier, 1834**										
Genus *Aulonium* Erichson										
Aulonium trisulcum (Fourcroy, 1785)	**UPOL 002279**	missing	FJ903787	missing	FJ904080	missing	FJ903951	missing	EF209944	- n/a -
Genus *Bitoma* Herbst										
Bitoma siccana Pascoe, 1863	**UPOL 002219**	missing	FJ903786	missing	FJ904079	missing	FJ903950	missing	EF209943	- n/a -
Genus *Colydium* Fabricius										
Colydium elongatum (Fabricius, 1787)	**UPOL ZL0195**	missing	EF490160	missing	EF490189	missing	FJ903934	missing	missing	- n/a -
Genus *Endophloeus* Dejean										
Endophloeus serratus Sharp, 1885	**UPOL ZL0143**	missing	FJ903750	missing	FJ903035	missing	FJ903900	missing	EF209939	- n/a -
Genus *Gempylodes* Pascoe										
Gempylodes lewisii Sharp, 1885	**UPOL ZL0067**	missing	EF490156	missing	EF490186	missing	FJ903848	missing	EF209938	- n/a -
Genus *Synchita* Hellwig										
Synchita humeralis (Fabricius, 1792)	**UPOL ZL0178**	missing	FJ903768	missing	FJ904058	missing	FJ903925	missing	EF209940	- n/a -

* For 18S, separate accession numbers a provided in case several non-overlapping fragments were sequenced
