# Supplementary material for: Cretaceous environmental changes led to high extinction rates in a hyperdiverse beetle family
Source: BMC Evol Biol. 2014 Oct 21;14:220. doi: 10.1186/s12862-014-0220-1 (PMC4210489; doi:10.1186/s12862-014-0220-1)
Supplement: Additional file 4: Table S4. — Number of genera per tribe. Sampled tribes are highlighted using bold characters. [file 12862_2014_220_MOESM4_ESM.pdf]

Additional Table S4

Number of genera per tribe. Sampled tribes are highlighted using bold characters.

Subfamily	Tribe	number of genera
Zolodininae	n/a	2
Lagriinae	***Adeliini***	***46***
	Belopini	6
	Chaerodini	***2***
	Cossyphini	2
	Goniaderini	21
	Laenini	9
	Lagriini	***130***
	Lupropini	***12***
	Pycnocerini	18
Nilioninae	n/a	1
Phrenapatinae	Archaeoglenini	1
	Penetini	***24***
	Phrenapatini	2
Pimeliinae	Adelostomini	24
	Adesmiini	***14***
	Akidini	***5***
	Anepsiini	5
	Asidini	***45***
	Boromorphini	1
	Branchini	3
	Caenocrypticini	6
	Ceratanisini	3
	Cnemeplatiini	8
	Cnemodinini	1
	Coniontini	4
	Cossyphodini	8
	Cryptochilini	13
	Cryptoglossini	3
	Edrotini	49
	Elenophorini	***3***
	Epitragini	29
	Erodiini	***28***
	Evasiosomini	4
	Falsomycterini	2
	Idisiini	1
	Klewariini	1

	Kuhitangiini	1
	Lachnogyini	4
	Leptodini	2
	Nycteliini	9
	Nyctoporini	1
	Phrynocarenini	1
	Physogasterini	4
	Pimeliini	***67***
	Praociini	9
	Sepidiini	30
	Stenosini	28
	Tentyriini	***90***
	Thinobatini	2
	Trilobocarini	5
	Vacronini	4
	Zophosini	17
Tenebrioninae	Acropteronini	2
	Alphitobiini	4
	Amarygmini	***58***
	Amphidorini	6
	Apocryphini	4
	Blaptini	***24***
	Bolitophagini	***18***
	Centronopini	3
	Cerenopini	2
	Dissonomini	2
	Eulabini	3
	Falsocossyphini	3
	Heleini	***39***
	Helopini	***41***
	Helopinini	15
	Melanimonini	***3***
	Opatrini	***100***
	Palorini	11
	Pedinini	***74***
	Platyscelidini	11
	Praeugenini	1
	Rhysopaussini	12
	Scaurini	***4***
	Scotobiini	5
	Tenebrionini	***9***
	Titaeini	***9***
	Toxicini	***18***
	Triboliini	***10***
	Ulomini	***18***

Alleculinae	***Alleculini***	***96***
	**Cteniopodini**	**24**
Diaperinae	***Crypticini***	***14***
	Diaperini	***40***
	Ectychini	2
	**Gnathidiini**	**24**
	Hyociini	***6***
	Hypophlaeini	5
	Leiochrinini	10
	Myrmechixenini	1
	Phaleriini	***8***
	Scaphidemini	***5***
	Trachyscelini	***3***
Stenochiinae	***Cnodalonini***	***326***
	Stenochiini	***46***
	Talanini	1
