# Supplementary material for: Cretaceous environmental changes led to high extinction rates in a hyperdiverse beetle family
Source: BMC Evol Biol. 2014 Oct 21;14:220. doi: 10.1186/s12862-014-0220-1 (PMC4210489; doi:10.1186/s12862-014-0220-1)
Supplement: Additional file 5: — Additional information on fossil taxa and justifications on ages of geological formations and amber deposits. [file 12862_2014_220_MOESM5_ESM.pdf]

Additional file S1. Additional information on fossil taxa and justifications on ages of geological formations and amber deposits.

- †*Elaterophanes vetustus* (Brodie 1845) /
  Elateroidea, Elateridae

It is a compression fossil represented by two specimens. Its type locality is Apperley (Gloucestershire), which is in a Hettangian terrestrial horizon in the United Kingdom. This fossil was described in Brodie [1] and was further recombined into *Elaterophanes vetustus* in Whalley [2]". For this fossil, we used the upper bound (199.0 Myr) of the Hettangian Stage (201.0 - 199.0 Ma; [3]) to set a minimum age for the root node. This fossil was clearly assigned to the family Elateridae by Whalley [2], who also suggested that it belongs to the tribe Hypnomorphini based on the shape of the femoral plates on the hind coxae and of the closure of the prosternal suture.

- †*Anhuistoma hyla* Lin 1985 /
  Cleroidea, Trogossitidae

Anhuistoma hyla was described by Lin [4]. Its type specimen is 2p43-H26-243, an exoskeleton (missing head), and it is an impression. Its type locality is Pengzhuang village, Hanshan County, which is in a Bajocian/Bathonian terrestrial sandstone/mudstone in the Hanshan Formation of China. For this fossil, we used the upper bound (166.0 Myr) of the Bathonian Stage (168.0 - 166.0 Ma; [3]) to set a minimum age for the crown/stem of Cleroidea. The assignation of *Anhuistoma hyla* to Cleroidea (and more specifically to the family Trogossitidae) was confirmed in the review of Schmied et al. [5].

- †*Eumolpites jurassicus* Martynov 1926 /
  Chrysomeloidea, *familia incerta*

Eumolpites jurassicus was described by Martynov [6]. It is an impression. Its type locality is Karatau (PIN collection 2452), which is in a Callovian/Oxfordian lacustrine siltstone in the Karabastau Formation of Kazakhstan. The geologic age of the Formation is still disputed, with estimates ranging from the Callovian to Oxfordian/Kimmeridgian Stages [7]. To avoid any potential biases we chose to use as a minimum age constraint the youngest age estimate (Kimmeridgian Stage) for this geological formation, which is based on paleoenvironmental [8] and floral assemblage [9] analyses. The same conservative choice was made in the study of Hunt et al. [10] and in several paleontological studies (e.g. [11,12]). As a result, for this fossil, we used the upper age (152.0 Myr) of the Kimmeridgian Stage (157.0 - 152.0 Ma; [3]) to set a minimum age for the crown/stem of Chrysomeloidea. According to Martynov, *Eumolpites jurassicus* is related to extant members of the subfamily Eumolpinae [6]. However, this precise assignment is possibly doubtful [13], in a way similar to those of other fossil chrysomelids from the same geological formation [13,14], which present cerambycid-like feature such as long antennae. Here we only consider this fossil as the earliest representative of the chrysomeloid lineage. By doing so, we also follow the views of Santiago-Blay [13] that considers that older reports of chrysomelid beetles are questionable.

- †*Belonotaris punctatissimus* Arnoldi 1977 /
  Curculionoidea, Nemonychidae

Belonotaris punctatissimus was described by Arnoldi [15]. Its type specimen is PIN 2452/422, an exoskeleton, and it is a compression fossil. Its type locality is Karatau, which is in a lacustrine siltstone in the Karabastau Formation of Kazakhstan". For this fossil, we used the upper age (152.0 Myr) of the Kimmeridgian Stage (157.0 - 152.0 Ma; [3]) to set a minimum age for the crown/stem of Curculionoidea. The placement of this fossil in Curculionoidea (and more specifically to the family

Nemonychidae) was confirmed in the recent review on Mesozoic Curculionoidea by Legalov [16]. Numerous fossils of Curculionoidea are known from the same geological formation [16-18].

- †*Wuhua jurassica* Wang and Zhang 2011 / Tenebrionoidea, *familia incerta*

Wuhua jurassica was described by Wang and Zang [19]. Its type specimen is NIGP 149548, an exoskeleton (nearly complete beetle), and it is a compression fossil. Its type locality is Daohugou, which is in a Callovian/Oxfordian lacustrine - extensive shale in the Jiulongshan Formation of China. It is the type species of *Wuhua*". As underlined by Wang and Zang [19], its tarsal formula 5-5-4 (heteromerous tarsi) undoubtedly places it in Tenebrionoidea. However, because of it is unusual combination of morphological characters it is not possible to place it in any existing family [19]. For this fossil, we used the upper age (157.0 Myr) of the Oxfordian Stage (164.0 - 157.0 Ma;[3]) to set a minimum age for the crown/stem of Tenebrionoidea.

- Undescribed Pyrochroidae in Kirejtshuk et al. (2010b) / Tenebrionoidea, Pyrochroidae

Unequivocal and diverse undescribed fossils from the family Pyrochroidae are represented in the Liaoning (Yixian) Formation [20]. The age of the Yixian Formation formerly was a contentious issue, with estimates ranging from the Late Jurassic to the Early Cretaceous [21,22]. This formation is constituted of lacustrine sediments intercalated with volcanoclastics [20]. Radiometric analyses [23,24] using ^{40}Ar/^{39}Ar provided estimates of 124.6 ± 0.1 Myr and 125.0. ± 0.18 Myr from three different tuff layers (in the 'Jianshangou beds'), which are in agreement with a ^{235}U/^{207}Pb zircon date of 125.2 ± 0.9 Myr for the same beds. An older age (^{40}Ar/^{39}Ar dating of 139.4 ± 0.19 Myr) was also inferred when analysing the basalt capping in the lowermost 'Lujiatun beds', thus suggesting a Hauterivian age for the base of the Yixian Formation [25]. Here we used the boundary between the Barremian and the Aptian Stages to set a minimum age of 126.0 Myr for the crown/stem of the Pyrochroidae.

- †*Praemordella martynovi* Scegoleva-Barovskaja 1929 / Tenebrionoidea, Mordellidae

Praemordella martynovi is considered as the earliest known occurrence of mordellids [26]. It is a nearly complete impression fossil with a distinctive mordellid wedge-shaped body and a terminal distinctive pygidium [26]. Its type locality is Karatau, which is in lacustrine siltstone within the Karabastau Formation of Kazakhstan". For this fossil, we used the upper age (152.0 Myr) of the Kimmeridgian Stage (157.0 - 152.0 Ma; [3]) to set a minimum age for the crown/stem of Mordellidae. The same fossil calibration was used in the study of Hunt et al. [10].

- †*Camelomorpha longicervix* Kirejtshuk, Azar and Telnov 2008 / Tenebrionoidea, Anthicidae

Camelomorpha longicervix was described by Kirejtshuk et al. [27]. Its type specimen is MNHN Azar 846, an exoskeleton, and it is an inclusion in amber. Its type locality is Mdeyrij-Hammana, Casa Baabda (Azar collection), which is an Aptian terrestrial amber in Lebanon. It is the type species of *Camelomorpha*". Though the ages of Lebanese amber may range from the Late Jurassic to the Albian Stage, the fossiliferous outcrops are approximately of the same age and are mainly late Barremian to early Aptian Stages [28-29]. For this site, we specified a minimum age of 120.0 Myr (see also [30]) that is slightly more conservative than the estimates of 125.5 - 122.5 Ma provided by the Paleobiology Database. This value was used to set a minimum age for the crown/stem

of Anthicidae. Because of the excellent state of preservation of the specimen, its description is extremely complete and detailed [31] and its assignment is secure.

- Undescribed Aderidae in Kirejtshuk and Azar (2008) and Kirejtshuk et al. (2009) / Tenebrionoidea, Aderidae

Unequivocal fossils from the family Aderidae are well represented in the Lower Cretaceous Lebanese amber[27]. Most of the material comes from the Mdeyrij-Hammana deposit [31]; therefore we used the value that was defined previously (120.0 Myr) to set a minimum age for the crown/stem of Aderidae.

- †*Jurallecula grossa* Medvedev 1969 / Tenebrionoidea, Tenebrionidae

From the Paleobiology Database (paleodb.org): "*Jurallecula grossa* was named by Medvedev [32]. Its type specimen is PIN 2239/1064, an exoskeleton (complete beetle), and it is an impression. Its type locality is the Mikhailovka locality (PIN collection 2239), which is in the large siltstone in the Karabastau Formation of Kazakhstan. It is the type species of *Jurallecula*". For this fossil, we used the upper age (152.0 Myr) of the Kimmeridgian Stage (157.0 - 152.0 Ma; [32]) to set a minimum age for the crown/stem of Tenebrionidae. Based on the connation of the basal abdominal ventrites and heteromerous tarsi, this fossil can be clearly assigned to the family Tenebrionidae [33]. Though this fossil was tentatively assigned to the subfamily Alleculinae by Medvedev [32], it is not possible to place it in any existing alleculine tribes [34]. The presence of pectinate claws also only partially point toward Alleculinae, as several alleculine species do not possess this character [33]. In addition, the presence of pectinate claws is a potentially homoplasious character, as they are present in several other tenebrionoid groups, including the enigmatic *Wuhua jurassica*. Therefore we cannot exclude the hypothesis that this fossil is a representative of an extinct lineage of tenebrionid beetles, with a unique combination of characters. In a conservative way we only assigned it to the crown/stem of Tenebrionidae.

- Undescribed Alleculinae in Kirejtshuk et al. (2010b) / Tenebrionoidea, Tenebrionidae

Unequivocal and diverse undescribed fossils from the subfamily Alleculinae are well represented from the Yixian Formation of Liaoning Province, northeastern China [20]. All these fossils originate from the Yixian Formation [20]; therefore we used the value that was defined previously for this Formation (upper age of the Barremian Stage, 131.0 - 126.0 Ma; [3]) to set a minimum age of 126 Myr for the crown/stem of Alleculinae.

- †*Bolitophagus* sp. / †*Isomira avula* Seidlitz 1898 / †*Pentaphyllus cioides* Kirejtshuk et al. 2008 / †*Tribolium* sp. / †*Uloma* sp. / Tenebrionoidea, Tenebrionidae

All these fossils are well-preserved specimens that have been found in Eocene Baltic amber [34-36]. Both stratigraphic [37,38]; and K-Ar radiometric studies [39] correlate and indicate that Baltic amber is of middle Lutetian Age, about 44.0 Ma [40]. A recent review made by Wolfe et al. [41] also supports this minimum age for the Baltic amber. Therefore we used this value to a minimum age of 44.0 Myr for the crown/stem of all these genera.

- †*Platydema antiquorum* Wickham 1912 / Tenebrionoidea, Tenebrionidae

Platydema antiquorum was described by Wickham [42]. It is a compression fossil. Its type locality is Florissant, Station 17, which is in a lacustrine shale assigned to a Chadronian land-mammal Age in the Florissant formation of Colorado. This species is one of the two *Platydema* species that have been described from the latest Eocene Florissant Formation of Colorado [42,43]. Based on stratigraphic [44] and K-Ar radiometric studies [45], the age of this Formation is estimated to be Priabonian (37.8 - 33.9 Ma; [3]). Therefore we use the upper bound of this Stage to set a minimum age of 33.9 Myr for the crown/stem of this genus. In addition to their general shape, the assignment of these fossils to the genus *Platydema* is supported by the diaperine shape of the palpus (with a terminal subtriangular joint) and by the distinct scutellar striae.

- †*Lorelus angulatus* Doyen and Poinar 1994 /
  Tenebrionoidea, Tenebrionidae

This species is one of the four *Lorelus* species that have been described in the study of Doyen and Poinar [46]. All these fossils are well-preserved specimens that have been found in likely early Miocene Dominican amber [34,46]. While age estimates based on foraminifera counts range from 15.0 to 20.0 Myr [47], older estimates based on coccoliths range between 30.0 to 45.0 Myr [48]. In a conservative way we used the upper boundary of 15.0 Myr to set a minimum age for the crown/stem of the genus *Lorelus*. Such a conservative choice limits the risk of overestimation of node ages and was made in previous studies relying on Dominican amber fossil calibrations (e.g. [49]).

- †*Gonocephalum pristinum* Heyden and Heyden 1866 /
  Tenebrionoidea, Tenebrionidae

This fossil species was found in the Late Miocene locality of Öhningen along the German-Swiss border [34]. It is a compression fossil. In a conservative way we used the upper boundary of the Miocene 5.3 Myr to set a minimum age for the crown/stem of the genus *Gonocephalum*.

References

1. Brodie PB: *A history of the fossil insects in the Secondary rocks of England.* London; 1845.
2. Whalley PES: The systematics and palaeogeography of the Lower Jurassic insects of Dorset, England. *Bull Brit Mus Nat Hist (Geol.)* 1985, **39:**107-189.
3. Gradstein FM, Ogg JG, Schmitz M, Ogg G: *The geologic time scale 2012*. Boston: Elsevier; 2012.
4. Lin QB. Insect fossils from the Hanshan formation at Hanshan County, Anhui Province. *Acta Paleontol Sinica* 1985, **24:**300-315.
5. Schmied H, Wappler T, Kolibac J: A new bark-gnawing beetle (Coleoptera, Trogossitidae) from the middle Eocene of Europe with a checklist of fossil Trogossitidae. *Zootaxa* 2009, **1993:**17-26.
6. Martynov AV: To the knowledge of fossil insects from Jurassic beds in Turkestan 5. On some interesting Coleoptera. *Ezhegodnik Russkogo Paleontologicheskogo Obshevtva* 1926, **5**:1-39.
7. Zhang J, Li H: New taxa of snipe flies (Diptera: Brachycera: Rhagionidae) in the Daohugou biota, China. *Paleontol J* 2012, **46:**157-163.
8. Polyanskii BV, Doludenko MP: On sedimentogenesis in the Upper Jurassic carbonate flysch beds of the Kara-Tau mountain range (Southern Kazakhstan). *Litol Polezn Iskop* 1978, **3:**78-88.
9. Kirichkova AI, Doludenko MP: New data on phytostratigraphy of the Jurassic deposits of Kazakhstan. *Stratigr Geol Korrel* 1996, **4:**35-52.
10. Hunt T, Bergsten J, Levkaničova Z, Papadopoulou A, St. John O, Wild R, Hammond PM, Ahrens D, Balke M, Caterino MS, Gómez-Zurita J, Ribera I, Barraclough TG, Bocakova M, Bocak L, Vogler AP. A comprehensive phylogeny of beetles reveals the evolutionary origins of a superradiation. *Science* 2007, **318:**1913-1916.

11. Rasnitsyn AP, Zhang H: Composition and age of the Daohugou hymenopteran (Insecta, Hymenoptera = Vespida) assemblage from Inner Mongolia, China. *Paleontol* 2004, **47:**1507-1517.
12. Mostovski MB: 2008. Contributions to the study of fossil snipe flies (Diptera: Rhagionidae): the genus *Protorhagio. Paleontol J* 2008, **42:**75-80.
13. Santiago-Blay JA: **Paleontology of leaf beetles**. In *Novel aspects of the biology of Chrysomelidae.* Edited by Jolivet PH, Cox ML, Petitpierre E. The Netherlands: Kluwer academic publishers; 1994:1-68.
14. Reid C: **A cladistic analysis of subfamilial relationships in the Chrysomelidae *sensu lato* (Chrysomeloidea)**. In *Biology, phylogeny, and classification of Coleoptera: papers celebrating the 80th birthday of Roy A. Crowson. Vol. II.* Edited by Pakaluk J, Slipinski SA. Warszawa: Muzeum I Instytut Zoologii PAN; 1995:559-631.
15. Arnoldi LV: Rhynchophora, in Mezozoiskie zhestkokryiye. *Trudy Paleontol Inst* 1977, **161:**142-176.
16. Zimmerman EC: *Australian Weevils (Coleoptera: Curculionoidea). I: Anthribidae to Attelabidae: the primitive weevils*. Csiro Publishing; 1994.
17. Legalov AA: Checklist of Mesozoic Curculionoidea (Coleoptera) with a description of new taxa. *Baltic J Coleopt* 2010, **10:**71-101.
18. Legalov AA: Fossil history of Mesozoic weevils (Coleoptera: Curculionoidea). *Insect Sci* 2012, **19:**683-698.
19. Wang B, Zhang H: The oldest Tenebrionoidea (Coleoptera) from the Middle Jurassic of China. *J Paleontol* 2011, **85:**266-270.
20. Kirejtshuk AG, Ponomarenko AG, Prokin AA, Chang H, Nikolajev GV, Ren D: Current knowledge of Mesozoic Coleoptera from Daohugou and Liaoning (Northeast China). *Acta Geol Sinica* 2010, **84:**783-792.
21. Zhou Z, Barrett PM, Hilton J: An exceptionally preserved lower Cretaceous ecosystem. *Nature* 2003, **421:**807-814.
22. Wang W, Zhang L, Zheng S, Ren D, Zheng Y, Ding Q, Zhang H, Li Z, Yang F: The age of the Yixian stage at the boundary of Jurassic-Cretaceous - the establishment and study of stratotypes of the Yixian stage. *Geol Rev* 2005, **51:**234-242.
23. Swisher CC III, Wang Y, Wang X, Xu X, Wang Y: Cretaceous age for the feathered dinosaurs of Liaoning, China. *Nature* 1999, **400:**58-61.
24. Swisher CC III, Wang X, Zhou Z, Wang Y, Jin F, Zhang J, Xu X, Zhang F, Wang Y: Further support for a Cretaceous age for the feathered-dinosaur beds of Liaoning, China: new $^{40}Ar/^{39}Ar$ dating of the Yixian and Tuchengzi formations. *Chin Sci Bull* 2002, **47:**135-138.
25. Wang S, Hu H, Li P, Wang Y: Further discussion on the geologic age of Sihetun vertebrate assemblage in western Liaoning, China: evidence from Ar-Ar dating. *Act Petrolog Sinica* 2001, **17:**663-668.
26. Liu M, Lu W, Ren D: A new fossil mordellid (Coleoptera: Tenebrionoidea: Mordellidae) from the Yixian formation of western Liaoning Province, China. *Zootaxa* 2007, **1415:**49-56.
27. Kirejtshuk AG, Nel A: New beetles of the suborder Polyphaga from the Lowermost Eocene French amber (Insecta: Coleoptera). *Ann Soc Entomol Fr (NS)* 2008, **44:**419-442.
28. Azar D, Nel A, Gèze R: Use of Lebanese amber inclusions in paleoenvironmental reconstruction, dating and paleobiogeography. *Acta Zool Cracoviensia* 2003, **46:**393-398.
29. Kirejtshuk AG, Azar D, Beaver RA, Mandelshtam MY, Nel A: The most ancient bark beetle known: a new tribe, genus and species from Lebanese amber (Coleoptera, Curculionidae, Scolytinae). *Syst Entomol* 2009, **34:**101-112.

30. Poinar GO: *Life in amber*. Stanford: Stanford University Press; 1992.
31. Kirejtshuk AG, Azar D: New taxa of beetles (Insecta, Coleoptera) from Lebanese amber with evolutionary and systematic comments. *Alavesia* 2008, **2:**15-46.
32. Medvedev LN: New Mesozoic Coleoptera (Cucujoidea) of Asia. *Paleontol J* 1969, **3:**108-113.
33. Matthews EG, Lawrence JF, Bouchard P, Steiner WE, Ślipiński SA: **11.14. Tenebrionidae Latreille, 1802**. In *Handbook of Zoology. A Natural History of the Phyla of the Animal Kingdom. Volume IV - Arthropoda: Insecta. Part 38. Coleoptera, Beetles. Volume 2: Systematics (Part 2).* Edited by Leschen RAB, Beutel RG, Lawrence JF. Berlin: Walter de Gruyter; 2010:574-659.
34. Kirejtshuk AG, Merkl O, Kernegger F: A new species of the genus *Pentaphyllus* Dejean, 1821 (Coleoptera: Tenebrionidae, Diaperinae) from the Baltic amber and checklist of the fossil Tenebrionidae. *Zoosyst Ross* 2008, **17:**131-137.
35. Klebs R: Über Bernstein Einschlüsse im allgemeinen und die Coleopteren meiner Bersteinsammlung. *Schr Phys-ökon Ges Konigsberg* 1910, **51:**217-242.
36. Larsson SG: Baltic amber - a palaeobiological study. *Entomonograph* 1978, **1:**1-192.
37. Kosmowska-Ceranowicz B, Müller C: Lithology and calcareous nannoplankton in amberbearing Tertiary sediments from boreholes Chłapowo (Northern Poland*). Bull Pol Acad Sci* 1985, **33:**119-128.
38. Kosmowska-Ceranowicz B: Charakterystyka mineralogiczno-petrograficzna bursztynonośnych osadów Eocenu w okolicach Chłapowo oraz osadów Paleogenu Północnej *Polski Biul Inst Geol* 1987, **356:**29-50.
39. Ritzkowski S: K-Ar-Altersbestimmungen der bernsteinführenden Sedimente des Samlandes (Paläogen, Bezirk Kaliningrad). *Metalla Bochum* 1997, **66:**19-23.
40. Engel MS: A monograph of the Baltic amber bees and evolution of the Apoidea (Hymenoptera). *Bull Am Mus Nat Hist* 2001, **259:**1-192.
41. Wolfe AP, Tappert R, Muehlenbachs K, Boudreau M, McKellar RC, Basinger JF, Garrett A: A new proposal concerning the botanical origin of Baltic amber. *Proc R Soc B* 2009, **276**:3403-3412.
42. Wickham HF: A report of some recent collections of fossil Coleoptera from the Miocene shales of Florissant. *Bull. Lab Nat Hist State Univ Iowa* 1912, **6:**3-38.
43. Wickham HF: Fossil Coleoptera from Florissant in the United States National Museum. *Proc US Natl Mus* 1913, **45:**283-303.
44. Epis RC, Chapin CE: *Stratigraphic nomenclature of the Thirty-nine Mile volcanic field, central Colorado.* Washington: US Geol Surv Bull; 1974.
45. Evanoff E, McIntosh WC, Murphey PC: **Stratigraphic summary and $^{40}Ar/^{39}Ar$ geochronology of the Florissant formation, Colorado**. In *Fossil flora and stratigraphy of the Florissant formation*. Edited by Evanoff E, Gregory-Wodzicki KM, Johnson KR. Colorado: Proceedings of the Denver Museum of Nature and Science, ser. 4. no. 1., Colorado, 2001; 1-16.
46. Doyen JT, Poinar GO Jr: Tenebrionidae from Dominican amber (Coleoptera). *Entomol Scand* 1994, **25:**27-51.
47. Iturralde-Vincent MA, MacPhee RDE: Age and palaeogeographic origin of Dominican amber. *Science* 1996, **138:**1850-1852.
48. Schlee D: Das Bernstein-Kabinett. *Stutt Beitrag Natur Ser C* 1990, **138:**1-100.
49. Ramirez SR, Gravendeel B., Singer RB, Marshall CR, Pierce NE: Dating the origin of the Orchidaceae from a fossil orchid with its pollinator. *Nature* 2007, **448:**1042-1045.
