# Supplementary material for: Cretaceous environmental changes led to high extinction rates in a hyperdiverse beetle family
Source: BMC Evol Biol. 2014 Oct 21;14:220. doi: 10.1186/s12862-014-0220-1 (PMC4210489; doi:10.1186/s12862-014-0220-1)
Supplement: Additional file 6: Table S5. — Comparison of age estimates (under either a BD or a Yule model of speciation) between crown calibration and stem calibration scenarios. For all calibration schemes the median ages and 95% HPD are reported. Bold italic fonts are used to highlight age estimates that are contradicted by known fossil evidences (for the stem calibration scenario). [file 12862_2014_220_MOESM6_ESM.pdf]

Additional Table S5

Comparison of age estimates (under either a BD or a Yule model of speciation) between crown calibration and stem calibration scenarios. For all calibration schemes the median ages and 95% HPD are reported. Bold italic fonts are used to highlight age estimates that are contradicted by known fossil evidences (for the stem calibration scenario).

Node	Clade	Model	Crown calibration scenario Median	Crown calibration scenario 95% HPD	Stem calibration scenario Median	Stem calibration scenario 95% HPD
1	Buprestoidea (Buprestidae)	BD	131.96	95.58 - 159.96	136.40	106.44 - 186.00
		Yule	154.40	118.88 - 176.46	146.70	100.69 - 186.97
2	Byrrhoidea (Byrrhidae)	BD	130.75	57.81 - 234.13	116.41	84.75 - 182.00
		Yule	124.13	71.31 - 186.65	105.55	42.84 - 163.08
3	Cleroidea	BD	217.88	185.73 - 235.21	213.61	189.36 - 227.31
		Yule	219.66	191.95 - 236.60	206.69	186.03 - 225.39
4	Chrysomeloidea	BD	156.30	152.04 - 177.51	***141.69***	129.78 - 157.23
		Yule	155.71	152.00 - 168.20	***139.95***	116.20 - 153.65
5	Curculionoidea	BD	164.18	152.03 - 186.74	***143.42***	129.15 - 159.33
		Yule	164.71	152.01 - 177.19	***139.33***	120.81 - 157.06
6	Tenebrionoidea	BD	218.46	210.27 - 228.34	207.88	200.95 - 215.31
		Yule	220.89	210.68 - 229.83	200.49	186.67 - 210.81
7	Aderidae	BD	128.49	120.00 - 142.65	***96.42***	49.25 - 128.24
		Yule	130.72	120.02 - 142.83	***101.52***	76.74 - 126.92
8	Anthicidae	BD	138.33	120.01 - 162.72	***111.73***	90.54 - 136.45
		Yule	136.28	120.00 - 158.92	***103.05***	75.19 - 122.58
9	Ciidae	BD	144.13	101.99 - 182.42	127.47	78.79 - 155.85
		Yule	138.99	93.72 - 182.24	136.10	103.05 - 160.27
10	Meloidae	BD	122.49	108.72 - 136.86	115.19	100.29 - 128.79
		Yule	127.77	112.40 - 147.30	102.34	85.72 - 117.17
11	Mordellidae	BD	152.60	152.03 - 160.35	***108.95***	97.39 - 119.35
		Yule	152.74	152.00 - 157.72	***115.70***	106.77 - 125.31
12	Mycetophagidae	BD	107.80	90.40 - 129.19	96.80	78.80 - 115.41
		Yule	104.30	84.94 - 139.08	97.61	81.29 - 116.14
13	Oedemeridae	BD	116.97	95.37 - 146.98	122.88	108.55 - 139.09
		Yule	137.42	101.22 - 175.36	105.43	78.31 - 123.02
14	Pyrochroidae	BD	127.29	126.00 - 135.38	***68.80***	53.05 - 89.66
		Yule	127.60	126.00 - 135.56	***64.45***	50.88 - 82.67
15	Tenebrionidae	BD	178.04	168.40 - 188.71	167.58	155.84 - 177.08
		Yule	180.05	169.56 - 191.62	165.86	157.47 - 178.15
16	Zopheridae	BD	145.85	122.15 - 175.71	141.81	122.50 - 156.66
		Yule	144.65	120.95 - 164.16	131.98	115.26 - 156.10
17	Alleculinae	BD	126.82	126.00 - 130.86	***114.36***	96.74 - 124.92
		Yule	126.83	126.00 - 130.81	***112.61***	90.63 - 125.61
18	Lagriinae	BD	148.03	138.98 - 154.64	142.21	133.76 - 150.93
		Yule	147.10	132.14 - 149.53	141.37	134.06 - 148.76
19	Pimeliinae / Pimeliini	BD	73.42	59.88 - 97.34	71.13	55.65 - 84.99
		Yule	72.44	61.63 - 84.12	65.58	55.53 - 79.46
20	Pimeliinae / other	BD	146.45	117.91 - 168.19	123.25	104.67 - 142.89
		Yule	144.29	116.69 - 171.77	128.35	106.01 - 144.98

21 Stenochiinae	BD	97.85	85.25 - 108.91	88.53	77.17 - 98.85
	Yule	100.22	88.76 - 109.90	91.15	74.98 - 103.09
22 Adeliini	BD	91.33	80.72 - 105.69	90.81	78.08 - 105.47
	Yule	95.13	82.69 - 113.95	78.62	64.62 - 92.12
23 Akiidini	BD	80.97	59.60 - 101.78	76.35	53.07 - 91.25
	Yule	82.49	52.03 - 107.92	71.11	53.88 - 89.11
24 Asidini	BD	62.80	47.94 - 74.39	56.10	39.12 - 72.81
	Yule	55.66	42.62 - 79.75	53.15	37.94 - 63.24
25 Blaptini	BD	68.26	53.99 - 79.22	62.90	42.71 - 74.11
	Yule	64.61	50.34 - 75.20	57.95	46.25 - 68.84
26 Heleini	BD	94.46	81.94 - 104.39	88.78	81.39 - 95.08
	Yule	94.53	87.38 - 107.20	83.21	73.71 - 91.19
27 Lagriini	BD	116.13	102.87 - 128.11	110.68	99.27 - 126.23
	Yule	119.08	112.07 - 128.01	110.22	101.07 - 122.58
28 Pedinini	BD	99.93	77.40 - 117.74	97.09	81.46 - 110.66
	Yule	102.64	81.84 - 115.80	89.91	76.75 - 102.22
29 Phaleriini	BD	70.64	52.11 - 89.13	73.83	54.82 - 89.60
	Yule	78.42	55.97 - 94.31	60.98	47.08 - 79.64
30 Tentyriini	BD	55.05	45.09 - 67.74	57.68	46.19 - 65.02
	Yule	59.33	37.83 - 63.89	48.85	38.48 - 60.53
31 Titaeini	BD	58.99	49.29 - 67.79	53.56	43.92 - 59.48
	Yule	59.51	49.07 - 69.47	53.04	45.36 - 60.94
32 Ulomini	BD	39.40	34.66 - 43.14	33.77	28.14 - 39.00
	Yule	40.97	35.58 - 46.27	36.02	29.98 - 41.84
33 Canarian *Pimelia*	BD	22.57	19.48 - 24.00	22.71	19.86 - 24.00
	Yule	22.85	20.01 - 24.00	21.92	18.86 - 24.00
