# Supplementary material for: Cretaceous environmental changes led to high extinction rates in a hyperdiverse beetle family
Source: BMC Evol Biol. 2014 Oct 21;14:220. doi: 10.1186/s12862-014-0220-1 (PMC4210489; doi:10.1186/s12862-014-0220-1)
Supplement: Additional file 7: Table S6. — Bayes factor scores (2 ln BF) from Comparisons of alternative calibration procedures of BRC analyses. [file 12862_2014_220_MOESM7_ESM.pdf]

Additional Table S6

Bayes factor scores (2 ln B_F) from comparisons of alternative calibration procedures of BRC analyses.

Calibration procedures	estimated mean -lnL	vs. BD Stem	vs. BD Crown	vs. Yule Stem	vs. Yule Crown
BD Stem	-219453.58	-	90.74	-7.78	13.98
BD Crown	-219498.95	-90.74	-	-98.52	-76.76
Yule Stem	-219449.69	7.78	98.52	-	21.76
Yule Crown	-219460.57	-13.98	76.76	-14.92	-

Though the stem calibration procedures are significantly more supported by the B_F comparisons, the fossil record does not support their age estimates. Out of the two crown calibration procedures, the BD crown is recovered as the best-fit model (B_F, difference of 76.76, according to the criterion 2 ln B_F > 10 (Kass & Raftery 1995).
