# Supplementary material for: Cretaceous environmental changes led to high extinction rates in a hyperdiverse beetle family
Source: BMC Evol Biol. 2014 Oct 21;14:220. doi: 10.1186/s12862-014-0220-1 (PMC4210489; doi:10.1186/s12862-014-0220-1)
Supplement: Additional file 8: Figure S2. — Timetree corresponding to the results of the best--fit calibration procedure implemented with BEAST (‘Yule crown’). Horizontal bars on nodes correspond to the 95% higher posterior probabilities (HPD) of age estimates. [file 12862_2014_220_MOESM8_ESM.pdf]

Additional figure S2

Timetree corresponding to the results of the best-fit calibration procedure implemented with BEAST (‘Yule crown’). Horizontal bars on nodes correspond to the 95% higher posterior probabilities (HPD) of age estimates.

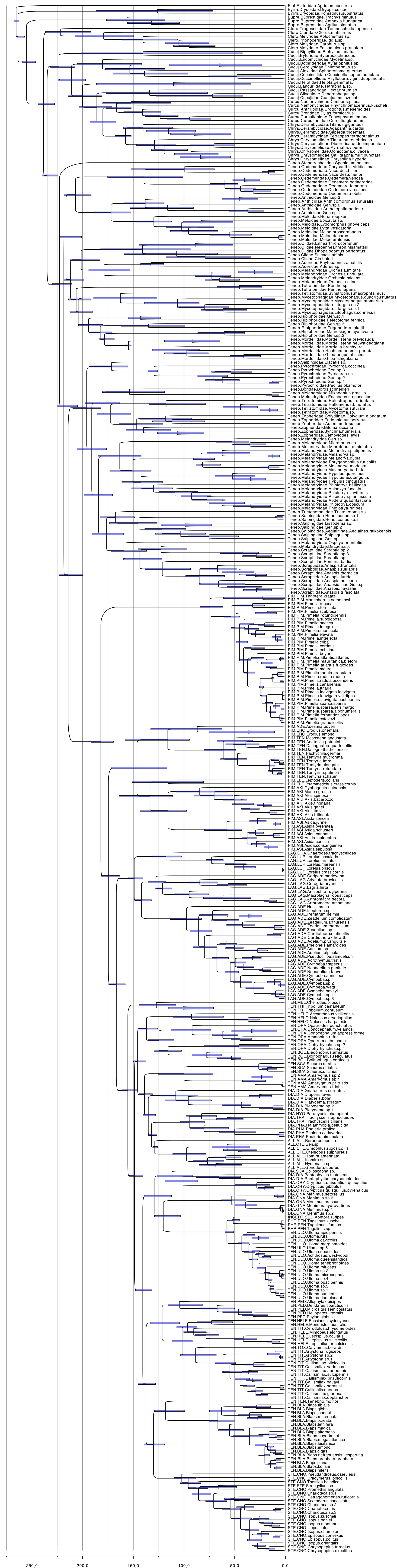
250,0
200,0
150,0
100,0
50,0
0,0
