# Supplementary material for: Cretaceous environmental changes led to high extinction rates in a hyperdiverse beetle family
Source: BMC Evol Biol. 2014 Oct 21;14:220. doi: 10.1186/s12862-014-0220-1 (PMC4210489; doi:10.1186/s12862-014-0220-1)
Supplement: Additional file 9: Figure S3. — Timetree corresponding to the results of the ‘BD crown’ calibration procedure implemented with BEAST.Horizontal bars on Nodes correspond to the 95% higher posterior probabilities (HPD) of age estimates. [file 12862_2014_220_MOESM9_ESM.pdf]

Additional figure S3

Timetree corresponding to the results of the ‘BD crown’ calibration procedure implemented with BEAST. Horizontal bars on nodes correspond to the 95% higher posterior probabilities (HPD) of age estimates.

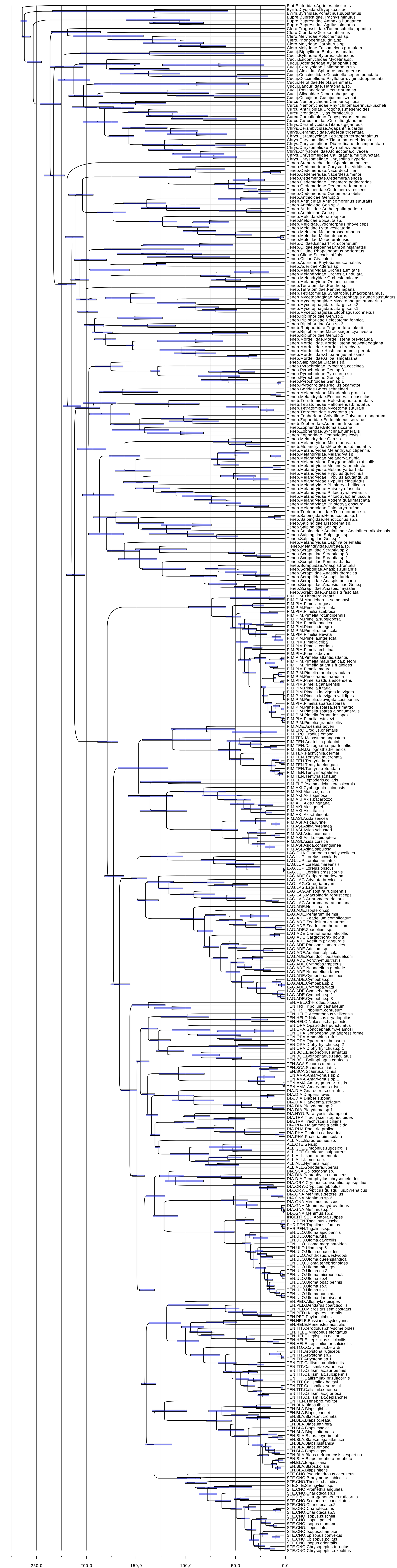
Elat.Elateridae.Agriotes.obscurus
Byrrh.Dryopidae.Dryops.costae
Byrrh.Byrrhidae.Pomatinus.substriatus
Bupre.Buprestidae.Trachys.minutus
Bupre.Buprestidae.Anthaxia.hungarica
Bupre.Buprestidae.Agrilus.sinuatus
Clero.Trogossitidae.Temnoscheila.japonica
Clero.Cleridae.Clerus.mutillarius
Clero.Melyridae.Aplocnemus.sp.
Clero.Prionoceridae.Idgia.sp.
Clero.Melyridae.Carphurus.sp.
Clero.Melyridae.Falsomelyris.granulata
Cucuj.Biphyllidae.Biphyllus.lunatus
Cucuj.Byturidae.Byturus.ochraceus
Cucuj.Endomychidae.Mycetina.sp.
Cucuj.Bothrideridae.Xylariophilus.sp.
Cucuj.Cerolynidae.Philothermus.sp.
Cucuj.Alexiidae.Sphaerosoma.quercus
Cucuj.Coccinellidae.Coccinella.septempunctata
Cucuj.Coccinellidae.Psyllobora.vigintiduopunctata
Cucuj.Helotidae.Helota.gemmata.
Cucuj.Languriidae.Tetraphala.sp.
Cucuj.Passandridae.Hectarthrum.sp.
Cucuj.Silvanidae.Dendrophagus.sp.
Cucuj.Cucujidae.Cucujus.mniszechi
Curcu.Nemonychidae.Cimberis.pilosa
Curcu.Nemonychidae.Rhynchitomacerinus.kuscheli
Curcu.Anthribidae.Urodontus.mesemoides
Curcu.Brentidae.Cylas.formicarius
Curcu.Curculionidae.Tanysphyrus.lemnae
Curcu.Curculionidaa.Curculio.glandium
Chrys.Cerambycidae.Titanus.giganteus
Chrys.Cerambycidae.Agapanthia.cardui
Chrys.Cerambycidae.Saperda.tridentata
Chrys.Cerambycidae.Tetraopes.tetraopthalmus
Chrys.Chrysomelidae.Timarcha.tenebricosa
Chrys.Chrysomelidae.Diabrotica.undecimpunctata
Chrys.Chrysomelidae.Pyrrhalta.viburni
Chrys.Chrysomelidae.Gonioctena.olivacea
Chrys.Chrysomelidae.Calligrapha.multipunctata
Chrys.Chrysomelidae.Chrysolina.hyperici
Teneb.Stenotrachelidae.Sponidium.pallens
Teneb.Oedemeridae.Chrysanthia.viridissima
Teneb.Oedemeridae.Nacerdes.hilleri
Teneb.Oedemeridae.Nacerdes.umenoi
Teneb.Oedemeridae.Oedemera.venosa
Teneb.Oedemeridae.Oedemera.podagrariae
Teneb.Oedemeridae.Oedemera.femorata
Teneb.Oedemeridae.Oedemera.virescens
Teneb.Oedemeridae.Oedemera.nobilis
Teneb.Anthicidae.Gen.sp.3
Teneb.Anthicidae.Anthicomorphus.suturalis
Teneb.Anthicidae.Gen.sp.2
Teneb.Anthicidae.Anthelephila.pedestris
Teneb.Anthicidae.Gen.sp.1
Teneb.Meloidae.Horia.roepkei
Teneb.Meloidae.Epicauta.sp.
Teneb.Meloidae.Lydomorphus.bifoveiceps
Teneb.Meloidae.Lytta.vesicatoria
Teneb.Meloidae.Meloe.proscarabaeus
Teneb.Meloidae.Meloe.decorus
Teneb.Meloidae.Meloe.uralensis
Teneb.Ciidae.Ennearthron.cornutum
Teneb.Ciidae.Neoennearthron.hisamatsui
Teneb.Ciidae.Rhopalodontus.perforatus
Teneb.Ciidae.Sulcacis.affinis
Teneb.Ciidae.Cis.boleti
Teneb.Aderidae.Phytobaenus.amabilis
Teneb.Aderidae.Aderus.sp.
Teneb.Melandryidae.Orchesia.imitans
Teneb.Melandryidae.Orchesia.undulata
Teneb.Melandryidae.Orchesia.micans
Teneb.Melandryidae.Orchesia.minor
Teneb.Tetratomidae.Penthe.sp.
Teneb.Tetratomidae.Penthe.japana
Teneb.Tetratomidae.Synstrophus.macrophtalmus.
Teneb.Mycetophagidae.Mycetophagus.quadripustulatus
Teneb.Mycetophagidae.Mycetophagus.atomarius
Teneb.Mycetophagidae.Litargus.sp.2
Teneb.Mycetophagidae.Litargus.sp.1
Teneb.Mycetophagidae.Litophagus.connexus
Teneb.Ripiphoridae.Gen.sp.1
Teneb.Ripiphoridae.Pelecotoma.fennica
Teneb.Ripiphoridae.Gen.sp.3
Teneb.Ripiphoridae.Trigonodera.lokejii
Teneb.Ripiphoridae.Macrosiagon.cyaniveste
Teneb.Ripiphoridae.Gen.sp.2
Teneb.Mordellidae.Mordellistena.brevicauda
Teneb.Mordellidae.Mordellistena.neuwaldeggiana
Teneb.Mordellidae.Mordella.brachyura
Teneb.Mordellidae.Hoshihananomia.perlata
Teneb.Mordellidae.Glipa.angustatissima
Teneb.Mordellidae.Glipa.ishigakiana
Teneb.Salpingidae.Elacatis.sp.
Teneb.Pyrochroidae.Pyrochroa.coccinea
Teneb.Pyrochroidae.Gen.sp.3
Teneb.Pyrochroidae.Pyrochroa.sp.
Teneb.Pyrochroidae.Gen.sp.2
Teneb.Pyrochroidae.Gen.sp.1
Teneb.Pyrochroidae.Pedilus.okamotoi
Teneb.Boridae.Boros.schneideri
Teneb.Melandryidae.Mikadonius.gracilis
Teneb.Melandryidae.Enchodes.crepusculus
Teneb.Tetratomidae.Holostrophus.orientalis
Teneb.Tetratomidae.Hallomenus.binotatus
Teneb.Tetratomidae.Mycetoma.suturale
Teneb.Tetratomidae.Mycetoma.sp.
Teneb.Zopheridae.Colydiinae.Colydium.elongatum
Teneb.Zopheridae.Endophloeus.serratus
Teneb.Zopheridae.Aulonium.trisulcum
Teneb.Zopheridae.Bitoma.siccana
Teneb.Zopheridae.Synchita.humeralis
Teneb.Zopheridae.Gempylodes.lewisii
Teneb.Melandryidae.Gen.sp.
Teneb.Melandryidae.Microtonus.sp.
Teneb.Melandryidae.Microtonus.dimidiatus
Teneb.Melandryidae.Melandrya.pictipennis
Teneb.Melandryidae.Melandrya.sp.
Teneb.Melandryidae.Melandrya.dubia
Teneb.Melandryidae.Phryganophilus.ruficollis
Teneb.Melandryidae.Melandrya.modesta
Teneb.Melandryidae.Melandrya.barbata
Teneb.Melandryidae.Hypulus.quercinus
Teneb.Melandryidae.Hypulus.acutangulus
Teneb.Melandryidae.Hypulus.cingulatus
Teneb.Melandryidae.Phloiotrya.bellicosa
Teneb.Melandryidae.Anisoxya.fuscula
Teneb.Melandryidae.Phloiotrya.flavitarsis
Teneb.Melandryidae.Phloiotrya.planiuscula
Teneb.Melandryidae.Abdera.quadrifasciata
Teneb.Melandryidae.Phloiotrya.obscura
Teneb.Melandryidae.Phloiotrya.rufipes
Teneb.Trictenotomidae.Trictenotoma.sp.
Teneb.Salpingidae.Henoticonus.sp.1
Teneb.Salpingidae.Henoticonus.sp.2
Teneb.Salpingidae.Lissodema.sp.
Teneb.Salpingidae.Gen.sp.2
Teneb.Salpingidae.Aegialitinae.Aegialites.raikokensis
Teneb.Salpingidae.Salpingus.sp.
Teneb.Salpingidae.Gen.sp.1
Teneb.Melandryidae.Osphya.orientalis
Teneb.Melandryidae.Dircaea.sp.
Teneb.Scraptiidae.Scraptia.sp.2
Teneb.Scraptiidae.Scraptia.sp.3
Teneb.Scraptiidae.Scraptia.sp.1
Teneb.Scraptiidae.Pentaria.badia
Teneb.Scraptiidae.Anaspis.frontalis
Teneb.Scraptiidae.Anaspis.rufilabris
Teneb.Scraptiidae.Anaspis.thoracica
Teneb.Scraptiidae.Anaspis.lurida
Teneb.Scraptiidae.Anaspis.pulicaria
Teneb.Scraptiidae.Anapsidiinae.Gen.sp.
Teneb.Scraptiidae.Anaspis.hayashii
Teneb.Scraptiidae.Anaspis.trifasciata
PIM.PIM.Thriptera.kraatzi
PIM.PIM.Mantichorula.semenowi
PIM.PIM.Pimelia.rugosa
PIM.PIM.Pimelia.fornicata
PIM.PIM.Pimelia.scabrosa
PIM.PIM.Pimelia.rotundipennis
PIM.PIM.Pimelia.subglobosa
PIM.PIM.Pimelia.baetica
PIM.PIM.Pimelia.integra
PIM.PIM.Pimelia.monticola
PIM.PIM.Pimelia.elevata
PIM.PIM.Pimelia.interjecta
PIM.PIM.Pimelia.criba
PIM.PIM.Pimelia.cordata
PIM.PIM.Pimelia.echidna
PIM.PIM.Pimelia.boyeri
PIM.PIM.Pimelia.atlantis.atlantis
PIM.PIM.Pimelia.mauritanica.bletoni
PIM.PIM.Pimelia.atlantis.frigioides
PIM.PIM.Pimelia.maura
PIM.PIM.Pimelia.radula.granulata
PIM.PIM.Pimelia.radula.radula
PIM.PIM.Pimelia.radula.ascendens
PIM.PIM.Pimelia.canariensis
PIM.PIM.Pimelia.lutaria
PIM.PIM.Pimelia.laevigata.laevigata
PIM.PIM.Pimelia.laevigata.validipes
PIM.PIM.Pimelia.laevigata.costipennis
PIM.PIM.Pimelia.sparsa.sparsa
PIM.PIM.Pimelia.sparsa.serrimargo
PIM.PIM.Pimelia.sparsa.albohumeralis
PIM.PIM.Pimelia.fernandezlopezi
PIM.PIM.Pimelia.estevezi
PIM.PIM.Pimelia.granulicollis
PIM.ADE.Adesmia.boyeri
PIM.ERO.Erodius.orientalis
PIM.ERO.Erodius.emondi
PIM.TEN.Mesostena.angustata
PIM.TEN.Anatolica.potanini
PIM.TEN.Dailognatha.quadricollis
PIM.TEN.Dailognatha.hellenica
PIM.TEN.Pachychila.germari
PIM.TEN.Tentyria.mucronata
PIM.TEN.Tentyria.latreilli
PIM.TEN.Tentyria.elongata
PIM.TEN.Tentyria.rotundata
PIM.TEN.Tentyrina.palmeri
PIM.TEN.Tentyria.schaumii
PIM.ELE.Leptoderis.collaris
PIM.ELE.Psammetichus.crassicornis
PIM.AKI.Cyphogenia.chinensis
PIM.AKI.Morica.grossa
PIM.AKI.Akis.spinosa
PIM.AKI.Akis.bacarozzo
PIM.AKI.Akis.tingitana
PIM.AKI.Akis.genei
PIM.AKI.Akis.italica
PIM.AKI.Akis.trilineata
PIM.ASI.Asida.sericea
PIM.ASI.Asida.jurinei
PIM.ASI.Asida.pyrenaea
PIM.ASI.Asida.schusteri
PIM.ASI.Asida.carinata
PIM.ASI.Asida.lepidoptera
PIM.ASI.Asida.corsica
PIM.ASI.Asida.consanguinea
PIM.ASI.Asida.sabulosa
LAG.CHA.Chaerodes.trachyscelides
LAG.LUP.Lorelus.occularis
LAG.LUP.Lorelus.armatus
LAG.LUP.Lorelus.mareensis
LAG.LUP.Lorelus.priscus
LAG.LUP.Lorelus.crassicornis
LAG.ADE.Coripera.morleyana
LAG.LAG.Adynata.brevicollis
LAG.LAG.Cerogria.bryanti
LAG.LAG.Lagria.hirta
LAG.LAG.Anisostira.rugipennis
LAG.LAG.Macrolagria.robusticeps
LAG.LAG.Arthromacra.decora
LAG.LAG.Arthromacra.amamiana
LAG.ADE.Nolicima.sp.
LAG.ADE.Isopteron.sp.
LAG.ADE.Periatrum.helmsi
LAG.ADE.Zeadelium.complicatum
LAG.ADE.Zeadelium.arthurensis
LAG.ADE.Zeadelium.thoracicum
LAG.ADE.Zeadelium.sp.
LAG.ADE.Cardiothorax.laticollis
LAG.ADE.Cardiothorax.howitti
LAG.ADE.Adelium.pr.angurale
LAG.ADE.Pheloneis.amaroides
LAG.ADE.Adelium.sp.
LAG.ADE.Adelium.alpicola
LAG.ADE.Pseudocilibe.samuelsoni
LAG.ADE.Acrothymus.tristis
LAG.ADE.Cymbeba.trapezus
LAG.ADE.Neoadelium.genitale
LAG.ADE.Neoadelium.fauveli
LAG.ADE.Cymbeba.annulipes
LAG.ADE.Cymbeba.sp.4
LAG.ADE.Cymbeba.sp.2
LAG.ADE.Cymbeba.watti
LAG.ADE.Cymbeba.bavayi
LAG.ADE.Cymbeba.sp.1
LAG.ADE.Cymbeba.sp.3
TEN.MEL.Cheirodes.pilosus
TEN.TRI.Tribolium.castaneum
TEN.TRI.Tribolium.confusum
TEN.HELO.Accanthopus.velikensis
TEN.HELO.Nalassus.dryadophilus
TEN.HELO.Nalassus.harpaloides
TEN.OPA.Opatroides.punctulatus
TEN.OPA.Gonocephalum.yelamosi
TEN.OPA.Gonocephalum.adpressiforme
TEN.OPA.Ammobius.rufus
TEN.OPA.Opatrum.sabulosum
TEN.OPA.Diphyrhynchus.sp.2
TEN.OPA.Diphyrhynchus.sp.1
TEN.BOL.Eledonoprius.armatus
TEN.BOL.Bolitophagus.reticulatus
TEN.BOL.Bolitophagus.corticola
TEN.SCA.Scaurus.atratus
TEN.SCA.Scaurus.striatus
TEN.SCA.Scaurus.uncinus
TEN.AMA.Amarygmus.sp.2
TEN.AMA.Amarygmus.sp.1
TEN.AMA.Amarygmus.pr.tristis
TEN.AMA.Amarygmus.tristis
DIA.DIA.Gnatocerus.cornutus
DIA.DIA.Diaperis.lewisi
DIA.DIA.Diaperis.boleti
DIA.DIA.Platydema.striatum
DIA.DIA.Platydema.sp.2
DIA.DIA.Platydema.sp.1
DIA.HYO.Parahyocis.championi
DIA.TRA.Trachyscelis.aphodioides
DIA.TRA.Trachyscelis.ciliaris
DIA.PHA.Halammobia.pellucida
DIA.PHA.Phaleria.prolixa
DIA.PHA.Phaleria.cadaverina
DIA.PHA.Phaleria.bimaculata
ALL.ALL.Borboresthes.sp.
ALL.CTE.Gen.sp.
ALL.CTE.Omophlus.rugosicollis
ALL.CTE.Cteniopus.sulphureus
ALL.ALL.Isomira.antennata
ALL.ALL.Isomira.sp.
ALL.ALL.Hymenalia.sp.
ALL.ALL.Gonodera.luperus
DIA.SCA.Spiloscapha.sp.
DIA.DIA.Pentaphyllus.testaceus
DIA.DIA.Pentaphyllus.chrysomeloides
DIA.CRY.Crypticus.quisquilius.quisquilius
DIA.CRY.Crypticus.gibbulus
DIA.CRY.Crypticus.quisquilius.pyrenaicus
DIA.GNA.Menimus.setosellus
DIA.GNA.Menimus.sp.3
DIA.GNA.Menimus.crassus
DIA.GNA.Menimus.hydrovatinus
DIA.GNA.Menimus.sp.1
DIA.GNA.Menimus.sp.2
INCERT.SED.Aphtora.rufipes
PHR.PEN.Tagalinus.kuscheli
PHR.PEN.Tagalinus.lifuanus
PHR.PEN.Tagalinus.sp.
TEN.ULO.Uloma.apicipennis
TEN.ULO.Uloma.rufa
TEN.ULO.Uloma.cavicollis
TEN.ULO.Uloma.marginatoides
TEN.ULO.Uloma.sp.5
TEN.ULO.Uloma.opacoides
TEN.ULO.Achthosus.westwoodi
TEN.ULO.Uloma.queenslandica
TEN.ULO.Uloma.tenebrionoides
TEN.ULO.Uloma.miriceps
TEN.ULO.Uloma.sp.2
TEN.ULO.Uloma.microcephala
TEN.ULO.Uloma.sp.4
TEN.ULO.Uloma.opacipennis
TEN.ULO.Uloma.sp.3
TEN.ULO.Uloma.sp.1
TEN.ULO.Uloma.punctata
TEN.ULO.Uloma.damoiseaui
TEN.PED.Allophylax.picipes
TEN.PED.Dendarus.coarcticollis
TEN.PED.Micrositus.semicostatus
TEN.PED.Heliopates.littoralis
TEN.PED.Phylan.gibbus
TEN.HELE.Bassianus.sydneyanus
TEN.HELE.Meneristes.australis
TEN.TIT.Cerodolus.chrysomeloides
TEN.HELE.Mimopeus.elongatus
TEN.HELE.Lepispilus.ocularis
TEN.HELE.Lepispilus.sulcicollis
TEN.HELE.Lepispilus.pr.sulcicollis
TEN.TOX.Calymmus.berardi
TEN.TIT.Artystona.rugiceps
TEN.TIT.Artystona.sp.2
TEN.TIT.Artystona.sp.1
TEN.TIT.Callismilax.plicicollis
TEN.TIT.Callismilax.variolosa
TEN.TIT.Callismilax.auripennis
TEN.TIT.Callismilax.sulcipennis
TEN.TIT.Callismilax.pr.ruficornis
TEN.TIT.Callismilax.bavayi
TEN.TIT.Callismilax.sarasini
TEN.TIT.Callismilax.aenea
TEN.TIT.Callismilax.gloriosa
TEN.TIT.Callismilax.deplanchei
TEN.TEN.Tenebrio.molitor
TEN.BLA.Blaps.tibialis
TEN.BLA.Blaps.gibba
TEN.BLA.Blaps.jeannei
TEN.BLA.Blaps.mucronata
TEN.BLA.Blaps.ocreata.
TEN.BLA.Blaps.lethifera
TEN.BLA.Blaps.magica
TEN.BLA.Blaps.alternans
TEN.BLA.Blaps.peyerimhoffi
TEN.BLA.Blaps.megalatlantica
TEN.BLA.Blaps.lusitanica
TEN.BLA.Blaps.emondi.
TEN.BLA.Blaps.gigas
TEN.BLA.Blaps.nefraouensis.vespertina
TEN.BLA.Blaps.propheta.propheta
TEN.BLA.Blaps.plana
TEN.BLA.Blaps.kollarii
TEN.BLA.Blaps.nitens
STE.CNO.Pseudandrosus.caeruleus
STE.CNO.Bradymerus.lobicollis
STE.CNO.Thesilea.baladica
STE.STE.Strongylium.sp.
STE.CNO.Promethis.angulata
STE.CNO.Charioteca.sp.1
STE.CNO.Tetragonomenes.ruficornis
STE.CNO.Scotoderus.cancellatus
STE.CNO.Charioteca.sp.2
STE.CNO.Charioteca.iris
STE.CNO.Charioteca.sp.3
STE.CNO.Isopus.kuscheli
STE.CNO.Isopus.paniei
STE.CNO.Isopus.montanus
STE.CNO.Isopus.latus
STE.CNO.Isopus.championi
STE.CNO.Episopus.convexus
STE.CNO.Episopus.politus
STE.CNO.Isopus.orientalis
STE.CNO.Chrysopeplus.triregius
STE.CNO.Chrysopeplus.expolitus
250,0
200,0
150,0
100,0
50,0
0,0
