# Supplementary material for: Cretaceous environmental changes led to high extinction rates in a hyperdiverse beetle family
Source: BMC Evol Biol. 2014 Oct 21;14:220. doi: 10.1186/s12862-014-0220-1 (PMC4210489; doi:10.1186/s12862-014-0220-1)
Supplement: Additional file 10: Table S7. — Biology/habitat of each of the 98 sampled tenebrionid genera. [file 12862_2014_220_MOESM10_ESM.pdf]

Additional Table S7

Biology/habitat of each of the 98 sampled tenebrionid genera.

Genus	Systematics	Biology/Habitat	Coded as
Borboresthes	Alleculinae, Alleculini	Open grasslands, temperate forests	0 (other)
Gonodera	Alleculinae, Alleculini	Open grasslands, temperate forests	0 (other)
Hymenalia	Alleculinae, Alleculini	Open grasslands, temperate forests	0 (other)
Isomira	Alleculinae, Alleculini	Open grasslands, temperate forests	0 (other)
Cteniopus	Alleculinae, Cteniopodini	Open grasslands, temperate forests	0 (other)
Omophlus	Alleculinae, Cteniopodini	Open grasslands, temperate forests	0 (other)
Crypticus	Diaperinae, Crypticini	Arid/semi-arid zones	1 (arid/semi-arid)
Diaperis	Diaperinae, Diaperini	Temperate forests	0 (other)
Gnatocerus	Diaperinae, Diaperini	Temperate forests	0 (other)
Pentaphyllus	Diaperinae, Diaperini	Temperate forests	0 (other)
Platydema	Diaperinae, Diaperini	Temperate/tropical rainforests, temperate forests	0 (other)
Menimus	Diaperinae, Gnathidiini	Temperate/tropical rainforests	0 (other)
Parahyocis	Diaperinae, Hyociini	Costal sand areas	1 (arid/semi-arid)
Halammobia	Diaperinae, Phaleriini	Costal sand areas	1 (arid/semi-arid)
Phaleria	Diaperinae, Phaleriini	Costal sand areas	1 (arid/semi-arid)
Spiloscapha	Diaperinae, Scaphidemini	Temperate/tropical rainforests	0 (other)
Trachyscelis	Diaperinae, Trachyscelini	Costal sand areas	1 (arid/semi-arid)
Adelium	Lagriinae, Adeliini	Temperate/tropical rainforests	0 (other)
Arcothymus	Lagriinae, Adeliini	Temperate/tropical rainforests	0 (other)
Cardiothorax	Lagriinae, Adeliini	Temperate/tropical rainforests	0 (other)
Coripera	Lagriinae, Adeliini	Temperate/tropical rainforests	0 (other)
Cymbeba	Lagriinae, Adeliini	Temperate/tropical rainforests	0 (other)
Isopteron	Lagriinae, Adeliini	Arid/semi-arid zones	1 (arid/semi-arid)
Neoadelium	Lagriinae, Adeliini	Temperate/tropical rainforests	0 (other)
Nolicima	Lagriinae, Adeliini	Temperate/tropical rainforests	0 (other)
Periatrum	Lagriinae, Adeliini	Temperate/tropical rainforests	0 (other)
Pheloneis	Lagriinae, Adeliini	Temperate/tropical rainforests	0 (other)
Pseudocilibe	Lagriinae, Adeliini	Temperate/tropical rainforests	0 (other)
Zeadelium	Lagriinae, Adeliini	Temperate/tropical rainforests	0 (other)
Chaerodes	Lagriinae, Chaerodini	Costal sand areas	1 (arid/semi-arid)
Adynata	Lagriinae, Lagriini	Temperate forests	0 (other)
Anisostira	Lagriinae, Lagriini	Temperate forests	0 (other)
Arthromacra	Lagriinae, Lagriini	Temperate forests	0 (other)
Cerogria	Lagriinae, Lagriini	Temperate forests	0 (other)
Lagria	Lagriinae, Lagriini	Temperate forests	0 (other)
Macrolagria	Lagriinae, Lagriini	Temperate forests	0 (other)
Lorelus	Lagriinae, Lupropini	Temperate/tropical rainforests	0 (other)
Tagalinus	Phrenapatinae, Penetini	Temperate/tropical rainforests	0 (other)
Adesmia	Pimeliinae, Adesmiini	Arid/semi-arid zones	1 (arid/semi-arid)
Akis	Pimeliinae, Akidini	Arid/semi-arid zones	1 (arid/semi-arid)

Cyphogenia	Pimeliinae, Akidini	Arid/semi-arid zones	1 (arid/semi-arid)
Morica	Pimeliinae, Akidini	Arid/semi-arid zones	1 (arid/semi-arid)
Asida	Pimeliinae, Asidini	Costal sand areas, arid/semi-arid zones	1 (arid/semi-arid)
Leptoderis	Pimeliinae, Elenophorini	Costal sand areas, arid/semi-arid zones	1 (arid/semi-arid)
Psammetichus	Pimeliinae, Elenophorini	Arid/semi-arid zones	1 (arid/semi-arid)
Erodius	Pimeliinae, Erodiini	Costal sand areas, arid/semi-arid zones	1 (arid/semi-arid)
Mantichorula	Pimeliinae, Pimeliini	Arid/semi-arid zones	1 (arid/semi-arid)
Pimelia	Pimeliinae, Pimeliini	Costal sand areas, arid/semi-arid zones	1 (arid/semi-arid)
Thriptera	Pimeliinae, Pimeliini	Arid/semi-arid zones	1 (arid/semi-arid)
Anatolica	Pimeliinae, Tentyriini	Arid/semi-arid zones	1 (arid/semi-arid)
Dailognatha	Pimeliinae, Tentyriini	Costal sand areas, arid/semi-arid zones	1 (arid/semi-arid)
Mesostena	Pimeliinae, Tentyriini	Arid/semi-arid zones	1 (arid/semi-arid)
Pachychila	Pimeliinae, Tentyriini	Costal sand areas, arid/semi-arid zones	1 (arid/semi-arid)
Tentyria	Pimeliinae, Tentyriini	Costal sand areas, arid/semi-arid zones	1 (arid/semi-arid)
Tentyrina	Pimeliinae, Tentyriini	Costal sand areas, arid/semi-arid zones	1 (arid/semi-arid)
Bradymerus	Stenochiinae, Cnodalonini	Temperate/tropical rainforests	0 (other)
Charioteca	Stenochiinae, Cnodalonini	Temperate/tropical rainforests	0 (other)
Chlorocamma	Stenochiinae, Cnodalonini	Temperate/tropical rainforests	0 (other)
Chrysopeplus	Stenochiinae, Cnodalonini	Temperate/tropical rainforests	0 (other)
Episopus	Stenochiinae, Cnodalonini	Temperate/tropical rainforests	0 (other)
Isopus	Stenochiinae, Cnodalonini	Temperate/tropical rainforests	0 (other)
Promethis	Stenochiinae, Cnodalonini	Temperate/tropical rainforests	0 (other)
Pseudandrosus	Stenochiinae, Cnodalonini	Temperate/tropical rainforests	0 (other)
Scotoderus	Stenochiinae, Cnodalonini	Temperate/tropical rainforests	0 (other)
Tetragonomenes	Stenochiinae, Cnodalonini	Temperate/tropical rainforests	0 (other)
Thesilea	Stenochiinae, Cnodalonini	Temperate/tropical rainforests	0 (other)
Strongylium	Stenochiinae, Stenochiini	Temperate/tropical rainforests, temperate forests	0 (other)
Amarygmus	Tenebrioninae, Amarygmini	Temperate/tropical rainforests	0 (other)
Blaps	Tenebrioninae, Blaptini	Costal sand areas, arid/semi-arid zones	1 (arid/semi-arid)
Bolitophagus	Tenebrioninae, Bolitophagini	Temperate forests	0 (other)
Eledonoprius	Tenebrioninae, Bolitophagini	Temperate forests	0 (other)
Bassianus	Tenebrioninae, Heleini	Temperate/tropical rainforests	0 (other)
Lepispilus	Tenebrioninae, Heleini	Temperate/tropical rainforests	0 (other)
Meneristes	Tenebrioninae, Heleini	Sclerophyll forests	0 (other)
Mimopeus	Tenebrioninae, Heleini	Temperate/tropical rainforests	0 (other)
Accanthopus	Tenebrioninae, Helopini	Temperate forests	0 (other)
Nalassus	Tenebrioninae, Helopini	Temperate forests	0 (other)
Cheirodes	Tenebrioninae, Melanimonini	Costal sand areas, arid/semi-arid zones	1 (arid/semi-arid)
Ammobius	Tenebrioninae, Opatrini	Costal sand areas	1 (arid/semi-arid)
Diphyrhynchus	Tenebrioninae, Opatrini	Costal sand areas	1 (arid/semi-arid)
Gonocephalum	Tenebrioninae, Opatrini	Arid/semi-arid zones	1 (arid/semi-arid)
Opatroides	Tenebrioninae, Opatrini	Costal sand areas, arid/semi-	1 (arid/semi-arid)

		arid zones	
Opatrum	Tenebrioninae, Opatrini	Costal sand areas, arid/semi-arid zones	1 (arid/semi-arid)
Allophylax	Tenebrioninae, Pedinini	Costal sand areas, arid/semi-arid zones	1 (arid/semi-arid)
Dendarus	Tenebrioninae, Pedinini	Costal sand areas, arid/semi-arid zones	1 (arid/semi-arid)
Heliopates	Tenebrioninae, Pedinini	Costal sand areas	1 (arid/semi-arid)
Micrositus	Tenebrioninae, Pedinini	Costal sand areas, arid/semi-arid zones	1 (arid/semi-arid)
Phylan	Tenebrioninae, Pedinini	Costal sand areas, arid/semi-arid zones	1 (arid/semi-arid)
Scaurus	Tenebrionini, Scaurini	Costal sand areas, arid/semi-arid zones	1 (arid/semi-arid)
Tenebrio	Tenebrioninae, Tenebrionini	Sclerophyll/temperate forests	0 (other)
Artystona	Tenebrioninae, Titaeini	Temperate/tropical rainforests	0 (other)
Callismilax	Tenebrioninae, Titaeini	Temperate/tropical rainforests	0 (other)
Cerodolus	Tenebrioninae, Titaeini	Temperate/tropical rainforests	0 (other)
Tribolium	Tenebrioninae, Triboliini	Temperate forests	0 (other)
Calymmus	Tenebrioninae, Toxicini	Temperate/tropical rainforests	0 (other)
Achthosus	Tenebrioninae, Ulomini	Temperate/tropical rainforests	0 (other)
Uloma	Tenebrioninae, Ulomini	Temperate/tropical rainforests, temperate forests	0 (other)
Aphtora	*Incertae sedis*	Temperate/tropical rainforests	0 (other)
